# Selective imaging and cancer cell death *via* pH switchable near-infrared fluorescence and photothermal effects†
†Electronic supplementary information (ESI) available: Synthesis, characterization, experimental details and other figures and spectra. See DOI: 10.1039/c6sc00221h


**DOI:** 10.1039/c6sc00221h

**Published:** 2016-05-26

**Authors:** Jingye Zhang, Zining Liu, Peng Lian, Jun Qian, Xinwei Li, Lu Wang, Wei Fu, Liang Chen, Xunbin Wei, Cong Li

**Affiliations:** a Key Laboratory of Smart Drug Delivery , Ministry of Education , School of Pharmacy , Fudan University , Shanghai 201203 , China . Email: congli@fudan.edu.cn; b State Key Laboratory of Oncogenes and Related Genes , Shanghai Cancer Institute , School of Biomedical Engineering , Shanghai Jiao Tong University , 1954 Huashan Road , Shanghai , 200030 , China . Email: xwei01@sjtu.edu.cn; c Department of Chemistry , National University of Singapore , Singapore 117543 , Singapore; d Department of Neurosurgery , Huashan Hospital , Fudan University , Shanghai 200040 , China

## Abstract

Accurately locating and eradicating sporadically distributed cancer cells whilst minimizing damage to adjacent normal tissues is vital in image-guided tumor ablation. In this work, we developed four heptamethine cyanine based theranostic probes, **IR1–4**, that demonstrated unique pH switchable near-infrared (NIR) fluorescence and photothermal efficiency. While their fluorescence quantum yields increased up to 1020-fold upon acidification from pH 7.4 to 2.4, their photothermal efficiencies decreased up to 7.1-fold concomitantly. Theoretical calculations showed that protonation of the probes in an acidic environment increased the orbital energy gaps and reduced the intramolecular charge transfer efficiency, resulting in the conversion of absorbed light energy to NIR fluorescence instead of hyperthermia. Substitutions at the terminal indole of the probes fine-tuned their p*K*a_fluo_ values to a narrow physiological pH range of 4.0–5.3. **IR2**, with a p*K*a_fluo_ of 4.6, not only specifically illuminated cancer cells by sensing their more acidic lysosomal lumen, but also selectively ablated cancer cells *via* its maximized photothermal effects in the alkaline mitochondrial matrix. As far as we are aware, these probes not only offer the highest physiological acidity triggered NIR fluorescence enhancement as small molecules, but are also the first to specifically visualize and eradicate cancer cells by sensing their altered pH values in cellular organelles. Considering that a disordered pH in organelle lumen is a common characteristic of cancer cells, these theranostic probes hold the promise to be applied in image-guided tumor ablation over a wide range of tumor subtypes.

## Abstract 2

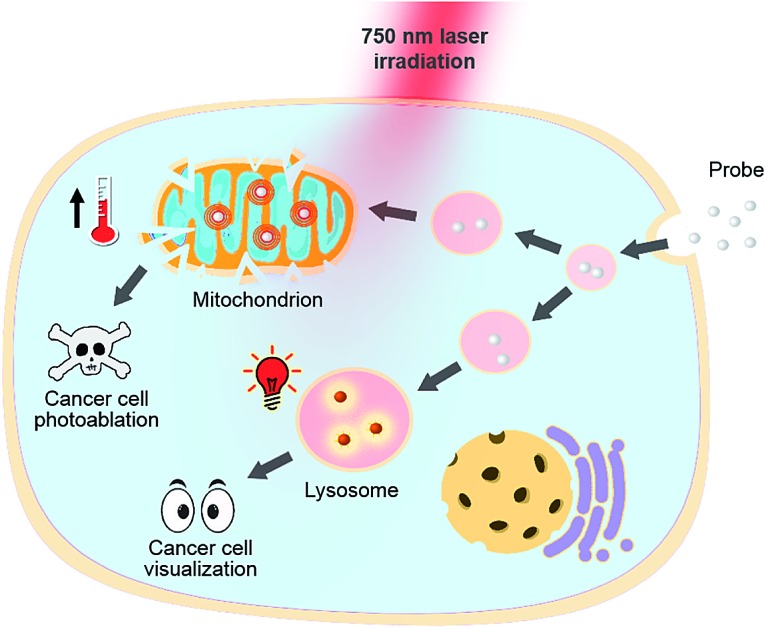
A theranostic probe is designed that specifically illuminates and photoablates cancer cells by sensing pH changes in the lysosomes and mitochondria.

## 1. Introduction

Cytoreductive surgery is the primary treatment for most solid tumors. Due to the invasive growth of cancer cells, however, clinicians encounter great challenges in identifying and completely removing the sporadically distributed tumor tissues. Residual neoplastic tissues post-surgical treatment usually lead to tumor recurrence and result in a poor prognosis. Intra-operative imaging shows great potential in guiding tumor resection by distinguishing neoplastic tissues with an improved target to background (T/B) ratio.^1^,^2^ Removing specific tumor foci amidst unresectable tissues such as major arteries, non-renewable nerves and intricate areas of the brain, however, is extremely difficult using conventional surgical instruments. The rapid development of image-guided tumor ablation^3^ has made it possible to precisely locate and eliminate tumor foci simultaneously with minimized damage to adjacent normal tissues. Theranostic probes for accurately locating and removing tumor foci intra-operatively are therefore invaluable for improving the surgical prognosis.

Of the multiple imaging modalities used for image-guided therapy, optical imaging shows advantages including high sensitivity, rapid acquisition rate, convenience in manipulation and affordable running costs. Due to the minimized absorption and autofluorescence from endogenous molecules in the near-infrared (NIR) wavelength window (650–900 nm), NIR fluorescence can detect tumor foci at millimeter depth with low photocytotoxicity.^4^ Additionally, the absorbed NIR photons can lead to local hyperthermia and kill cancer cells with heat vulnerability.^5^ Visualizing and eliminating cancer cells simultaneously therefore depends on the availability of NIR theranostic probes. The FDA approved NIR fluorescent probe indocyanine green (ICG) not only successfully guided excisions of hepatocellular carcinomas and colorectal hepatic metastases in patients,^6^ but also efficiently photoablated tumors after NIR light irradiation.^7^ To accurately assess cytoreductive tumor surgery, Ntziachristos *et al.* showed the first proof-of-principle experiment in excising ovarian tumors in patients using the guidance of a folate receptor targeted fluorescence probe.^8^ The broad applicability of the receptor targeting strategy, however, is limited because these receptors are only expressed in a small percentage of patients. For example, less than 25% of breast cancer patients over-express Her2/neu,^9^ which is the most widely used biomarker for breast tumor targeted treatment. Furthermore, non-specific accumulation of the probes with an “always on” signal in normal tissues attenuates the T/B ratio. As a result, NIR theranostic probes that can localize tumors with wider applicability and improved T/B ratios are urgently needed.

Acidic tumor extracellular fluid (pH 6.2–6.9) is a hallmark of solid tumors, regardless of their genotypes or phenotypes.^10^ pH responsive NIR fluorescence probes have shown applicability in visualizing multiple types of tumors.^11^–^13^ Besides the pH discrepancy in extracellular fluids, the lysosomal pH (pH_lys_) in cancer cells (pH_lys_ 3.8–4.7) also shows a higher acidity than that in normal cells (pH_lys_ 4.5–6.0).^14^ This results in accelerated invasion and metastasis of cancer cells by maximizing the enzymatic activity of the exocytosed proteases that actively degrade the extracellular matrix.^15^ Compared to probes that are triggered in the extracellular fluid, probes activated inside lysosomes could offer higher T/B ratios because the background noise from fluorophores that have diffused into normal tissues can be minimized. Even though numerous lysosomal targeted theranostic probes have been developed,^16^ their therapeutic efficiency is less satisfactory due to the up-regulated self-restoration of the lysosomal membrane in cancer cells,^17^ which prevents lysosomal membrane permeability associated apoptosis.^18^ As another important organelle in eukaryotic cells, mitochondria play crucial roles in regulating energy production and triggering programmed cell death (PCD) by releasing pro-apoptotic proteins from mitochondria into the cytosol.^19^ Considering the high sensitivity of mitochondria to heat shock,^20^ disrupting the mitochondrial membrane *via* photothermal effects is a promising approach for eradicating cancer cells. For example, mitochondria targeted carbon nanotubes^21^ and gold nanoparticles^22^ were shown to kill cancer cells efficiently *via* NIR light induced hyperthermia. Additionally, due to the continuous proton pumping process across the mitochondrial inner membrane during oxidative phosphorylation (OXPHOS), the mitochondrial matrix demonstrates a unique alkaline environment (pH_mito_ 7.5–8.2).^23^ There is promise therefore in selectively visualizing and eradicating cancer cells by sensing the altered pH_lys_ and pH_mito_.

**Scheme 1:**
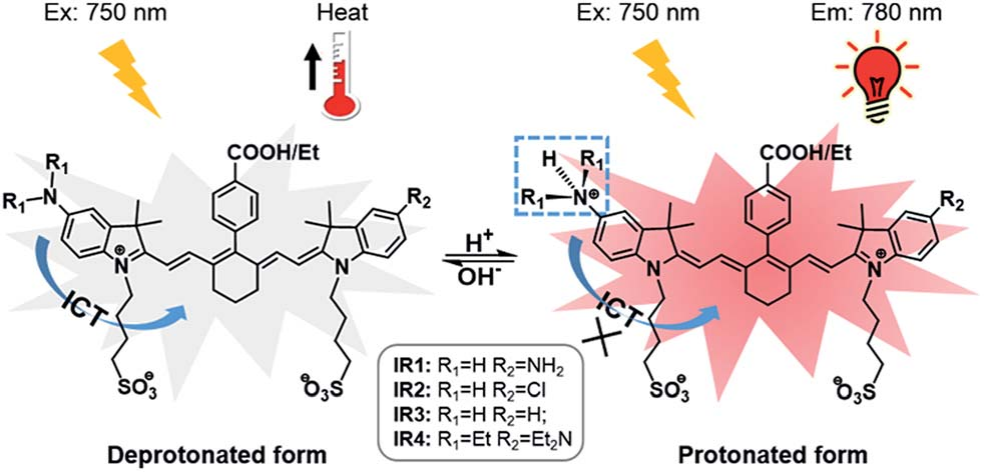
Structures of theranostic probes **IR1–4** and the proposed mechanism of pH switchable NIR fluorescence and photothermal effects.

**Fig. 1:**
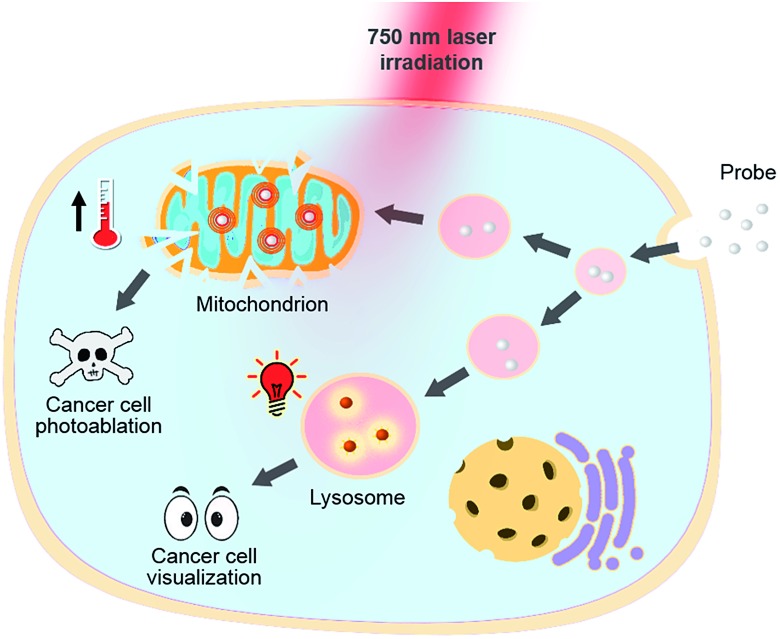
pH responsive theranostic probe specifically imaging and photoablating cancer cells with activated NIR fluorescence in acidic lysosomal lumen and maximized photothermal effects in an alkaline mitochondrial matrix.

Heptamethine cyanines (Hcyanines) are widely used in developing NIR theranostic probes because of their high extinction coefficients (*ε*) and fluorescence quantum yields (*Φ*), good biocompatibility and photothermal/photodynamic effects.^24^ The first pH responsive Hcyanine probe to be reported sensed physiological acidity *via* a photo-induced electron transfer (PET) mechanism.^25^ Only a moderate fluorescence enhancement (2.5–3.5 times) was achieved, however, which was explained by the reduced energy gap between the highest occupied molecular orbital (HOMO) and lowest unoccupied molecular orbital (LUMO) in the NIR region.^26^ We previously developed a Hcyanine probe based on self-aggregation induced quenching effects that offered a 15-fold fluorescence enhancement in physiological acidity.^12^,^27^ Recently, a hemicyanine derivative displayed a greater than 30-fold signal enhancement *via* an acidity triggered intramolecular spirocyclization mechanism.^28^ These probes, however, are not suitable for sensing pH_lys_ due to the difficulty in fine tuning their p*K*a values. Intramolecular charge transfer (ICT) is a mechanism by which electrons are transferred from the electron donor (D) to acceptor (A) within the fluorophore.^29^ Displaying rapid and reversible responses to external stimuli,^30^ many ICT fluorescence probes have been developed to detect solvent polarity,^31^ metal ions^32^ and gasotransmitters.^33^ However, very few of them are reported to sense physiological acidity in the NIR region.^34^ In this work, we developed four Hcyanine based theranostic probes (**IR1–4**) that not only showed unprecedented pH switchable NIR fluorescence and photothermal effects (Scheme 1), but also possessed p*K*a_fluo_ values in the pH_lys_ range by adjusting the ICT efficiency. While acidification from pH 7.4 to 4.0 led to a maximal 213-fold fluorescence quantum yield enhancement, the photothermal efficiencies decreased as low as 5.6-fold. As far as we are aware, **IR1–4** demonstrate the highest physiological acidity triggered NIR fluorescence enhancement as small molecular probes. Of these probes, **IR2**, with satisfactory photostability, demonstrated intracellular uptake in both the lysosomes and mitochondria of multiple human cancer and normal cell lines (Fig. 1). Due to its optimized p*K*a_fluo_ (4.6), NIR fluorescence activation of **IR2** in the lysosomes of cancer cells only, *i.e.* not in normal cells, was observed. Importantly, the combined use of **IR2** and 750 nm laser irradiation led to cancer cell death with percentages of 58.7–74.8%. In contrast, only 19.8–32.9% viability loss was determined for the normal cells. Apart from instant cell death after the combined treatment, the remaining living cells continued to undergo apoptosis that was induced by mitochondrial membrane disruption. Overall, these pH responsive theranostic probes showed great potential in image-guided tumor ablation by precisely positioning and destroying tumor foci intra-operatively.

## 2. Results and discussion

### 2.1. Design, synthesis and characterization of NIR theranostic probes

Cyanine dyes are composed of two terminal heterocyclic units linked by a polymethine bridge. Hcyanine derivative IR783 was chosen as a NIR fluorophore prototype to develop the theranostic probes because of its good biocompatibility, optimized emission wavelength and intracellular delivery into lysosomes and mitochondria *via* organic anion transporting peptide (OATP).^35^ A *p*-benzoic acid group was substituted on the meso position of IR783 *via* a C–C bond to increase its photostability and quantum yield.^36^ To achieve a pH response, an amine moiety with electron donating capability was incorporated at the 5-position on one terminal indole ring. At neutral pH, electron transfer from the amine to the Hcyanine will fully quench the fluorescence through non-radiative decay. In acidic environments, protonation of the amine will block the ICT process and the fluorescence will be regenerated. In order to trigger NIR fluorescence, specifically in the lysosomal lumen of cancer cells, selected functionalities with differing electron withdrawing capabilities were substituted on the other terminal indole. In this way, the p*K*a of the probes could be fine-tuned by adjusting the electron density on the Hcyanine scaffold. The structures of Hcyanine based theranostic probes **IR1–4** are illustrated in Scheme S1.† All the probes were fully characterized by ^1^H NMR spectroscopy, ^13^C NMR spectroscopy, high resolution mass spectrometry (HRMS), and high performance liquid chromatography (HPLC) (spectra in ESI†).

### 2.2. pH dependent optical and photothermal properties

#### 2.2.1. pH dependent absorption

**Fig. 2:**
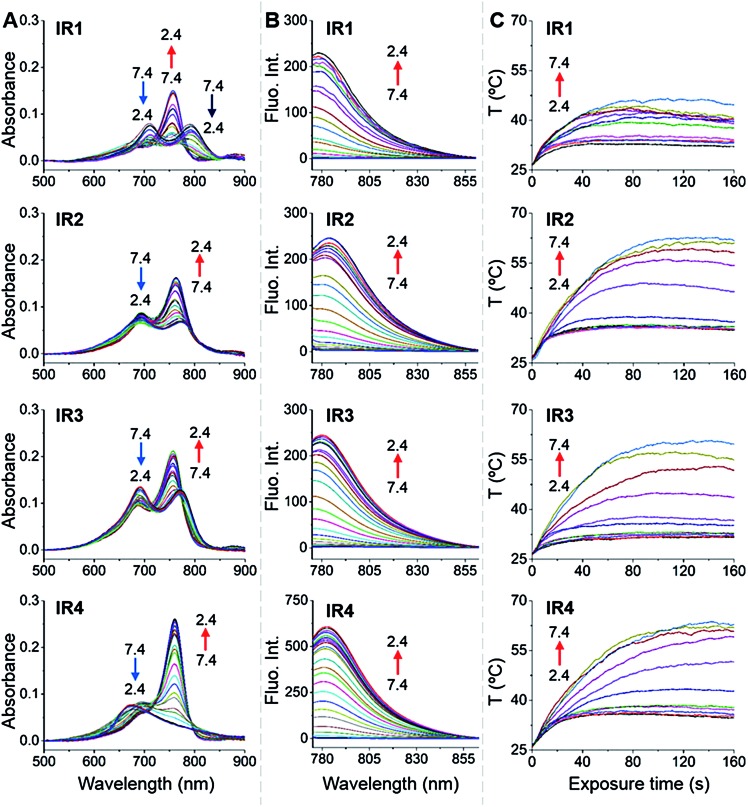
**IR1–4** showing pH dependent photophysical properties and photothermal efficiencies. (A) pH dependent absorption spectra of **IR1–4** (1.0 μM). (B) pH dependent emission spectra of **IR1–4** (1.0 μM, *λ*_ex_ = 750 nm). The pH changed at intervals of 0.2 units. (C) pH dependent temperature increase profiles of **IR1–4** (10 μM) as a function of laser irradiation time (750 nm, 6.0 W cm^–2^). The pH changed at intervals of 0.5 units.

In general, all the theranostic probes displayed absorption (600–850 nm) in the NIR wavelength range (Fig. 2A). At neutral pH, **IR1–3** possess two absorption peaks that were attributed to fluorophore monomers (longer wavelength) and aggregates (lower wavelength).^37^ Substitution of functional groups at the secondary heterocycle altered the absorbance wavelength of both the monomers (*λ*_mon_) and aggregates (*λ*_agg_). Compared to the unsubstituted analogue **IR3**, *λ*_mon_ of **IR1**, modified with a primary amine, red-shifted by 21 nm, which could be explained by the extension of the conjugated chain *via* the formation of p–π conjugation. A slight bathochromic shift (3.0 nm) was also observed in **IR2** upon chlorine substitution. Acidification led to obvious hypsochromic shifts of *λ*_mon_ with values of 37, 13 and 15 nm for **IR1**, **IR2** and **IR3** respectively. Protonation of the amine with concomitant breakdown of the p–π conjugation led to the hypsochromic shifts of absorption.^38^ Acidification also led to the increase of the monomers' absorbance (*A*_mon_) and the decrease of the aggregates' absorbance (*A*_agg_), which indicates conversion from aggregates to monomers.^39^ For example, the *A*_mon_/*A*_agg_ ratio of **IR2** increased from 0.85 (pH 7.4) to 1.64 (pH 2.4). Interestingly, while no obvious *A*_mon_ was detected at neutral pH, **IR4** displayed a broad absorbance that was attributed to H-type aggregates (multiple aggregates).^37^ This H-band diminished with the appearance of a D-band (dimeric aggregates) and an M-band (monomers)^37^ in acidic environments. The *N*-alkylated amines and benzylcarboxyl ester at the *meso*-position remarkably increased the lipophilicity of **IR4** and facilitated its formation of aggregates. This aggregation was also evident *via* broad and unresolved peaks in the ^1^H NMR spectrum.^40^

#### 2.2.2. pH switchable NIR fluorescence and photothermal effects

**Fig. 3:**
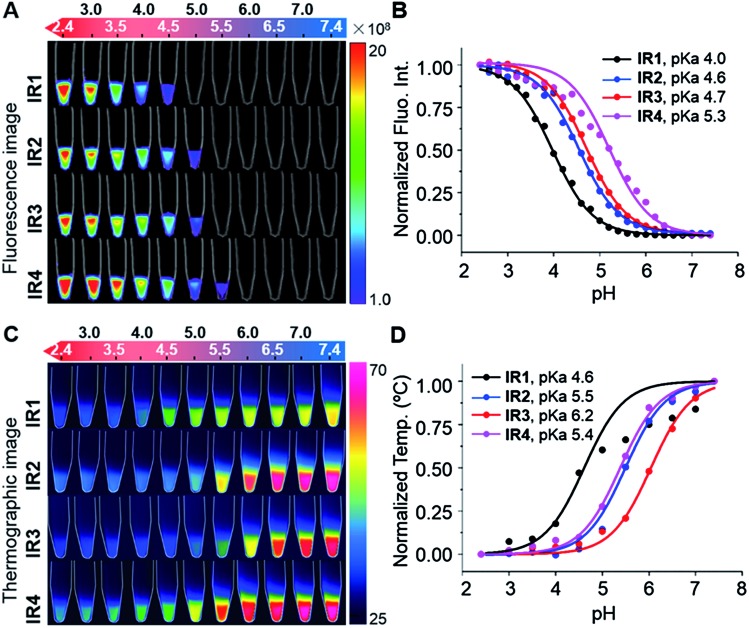
**IR1–4** showing pH switchable NIR fluorescence and photothermal effects. (A) NIR fluorescence image of the probes (1.0 μM) as a function of pH. Bar units: p s^–1^ cm^–2^ Sr^–1^. (B) pH dependent NIR fluorescence intensities normalized to their maximal values measured at pH 2.4. (C) Thermographic image of the probes (10 μM) after 750 nm laser irradiation (6.0 W cm^–2^) for 160 s. Bar units: °C. (D) pH dependent temperature increments (Δ*T*) normalized to their maximal values measured at pH 7.4.

As shown in Fig. 2B, all the probes were quenched at neutral pH. Gradual acidification resulted in a significant fluorescence enhancement with a peak centered at 778–784 nm. While the quantum yields (*Φ*) of **IR1–4** remained below 0.1% at pH 7.4, these values increased to 9.2%, 4.6%, 6.1% and 10.2% respectively at pH 2.4 (Table 1). As far as we are aware, these probes offer the highest acidity triggered NIR fluorescence intensity enhancement as small molecules.^41^,^42^ All the theranostic probes demonstrated pH dependent photothermal effects upon 750 nm laser irradiation (Fig. 2C). In contrast to the acidity-correlated increase in NIR fluorescence intensity, the photothermal efficiencies of the probes decreased with acidification. At pH 7.4, the temperature increments (Δ*T*) of **IR1–4** (10 μM) were measured to be 21, 37, 35 and 38 °C respectively after 750 nm laser irradiation (6.0 W cm^–2^). Δ*T*, however, was determined to be less than 10 °C for all the probes at pH 2.4. The photothermal stabilities (750 nm, 6.0 W cm^–2^) of **IR1–4** (10 μM) were also studied at pH 7.4 and pH 4.0 for up to 10 min. Notably, all the probes except **IR1** showed sufficient photophysical stability and Δ*T* were maintained at their plateau values for at least 4 min under continuous laser irradiation (Fig. S1†). The probes' photothermal efficacies also showed a dependence on the laser power and concentration (Fig. S2†). For instance, when the laser power was increased 5-fold, from 1.2 W cm^–2^ to 6.0 W cm^–2^, Δ*T* of **IR2** increased 26-fold correspondingly from 1.5 °C to 39 °C at pH 7.4. Meanwhile, a 20-fold change in concentration from 1.0 μM to 20 μM led to a 55-fold Δ*T* enhancement. When analogous experiments were performed at pH 4.0, Δ*T* barely exceeded 10 °C. The conversion of the absorbed energy to either NIR fluorescence or photothermia can be explained by the pH dependent ICT efficiency. Since the probes contain both an electron donor (bridgehead amine) and acceptor (Hcyanine), a charge separation is obtained within the fluorophore. The fully quenched signal under neutral pH can be ascribed to the formation of a non-fluorescent ICT state^43^ in which the stabilized charge transfer excited state becomes closer to the triplet excited state as well as the ground states.^44^ This therefore increases the rates of intersystem crossing and internal/external conversion *via* non-radiative decay, resulting in local hyperthermia.^45^ In contrast, the protonation of the bridgehead amine under acidic environments delocalizes the conjugated system and disrupts the charge transfer. A higher energy gap through relaxation of the locally excited (LE) state (without charge separation) to the ground state was formed.^43^ Therefore, the probes display a fluorescence response under acidic environments as the energy is released mainly through radiative decay as opposed to non-radiative relaxation. Additionally, self-aggregation of the cyanine dyes in aqueous solution was thoroughly investigated.^37^ Two exciton states arise in the case of face to face stacked dimer aggregates and subsequent rapid internal conversion of the excited state into the lower energy exciton state quenches the fluorescence due to the decreasing radiative transition probability from the exciton state to the ground state.^46^ At acidic pH, the electrostatic repulsion induced by the protonated terminal amines inhibits the self-quenching and hence retriggers the NIR fluorescence.^39^ The pH switchable NIR fluorescence and photothermal effects enable the theranostic probes to be activated in the acidic lysosomal lumen and the alkaline mitochondrial matrix simultaneously. NIR fluorescence images of the probes (1.0 μM) in buffered solutions with the pH decreasing from 7.4 to 2.4 show their progressive fluorescence enhancement (Fig. 3A). A plot of fluorescence intensity *versus* pH gave the acid dissociation constants (p*K*a_fluo_) of **IR1–4** as 4.0, 4.6, 4.7 and 5.3 respectively (Fig. 3B). Fig. 3C shows the thermographic maps of **IR1–4** at selected pH values after 750 nm laser irradiation. Plotting the pH dependent temperature increments gave the thermal energy transition points (p*K*_Δ*T*_) of **IR1–4** to be 4.6, 5.5, 6.2 and 5.4 respectively (Fig. 3D). Substitution of functional groups on the terminal indole was hypothesised to alter the electron density on the Hcyanine scaffold and hence changed the pH transition point of the bridgehead amine. In this way, it is possible to obtain a probe with a fine-tuned p*K*a_fluo_ to specifically signal pH_lys_ in cancer cells. On account of its optimized p*K*a value, sufficient photostability, satisfactory pH responsive NIR fluorescence and photothermal efficiency, **IR2** was chosen as a theranostic probe to specifically visualize and kill cancer cells *in vitro*.

**Table 1:** Photophysical parameters of theranostic probes **IR1–4**

Probe	*λ*_mono_ ^*a*^ (nm)	*λ*_agg_ ^*b*^ (nm)	*λ*_em_ ^*c*^ (nm)	p*K*a_fluo_	*Φ* ^*d*^ (%)	p*K*a_Δ*T*_
**IR1**	792^*e*^	712^*e*^	778	3.99	0.03^*e*^	4.58
	755^*f*^	689^*f*^			9.19^*f*^	
**IR2**	774^*e*^	691^*e*^	784	4.56	0.05^*e*^	5.51
	761^*f*^	694^*f*^			4.63^*f*^	
**IR3**	771^*e*^	691^*e*^	780	4.71	0.05^*e*^	6.16
	756^*f*^	688^*f*^			6.07^*f*^	
**IR4**	N.D.^*g*^	677^*e*^	783	5.28	0.01^*e*^	5.39
	760^*f*^	696^*f*^			10.20^*f*^	

^*a*^Maximal absorption wavelength of monomers.

^*b*^Maximal absorption wavelength of aggregates.

^*c*^Maximal emission wavelength.

^*d*^Quantum yield of the probes correlated to ICG (Q.Y._ICG_ = 0.12 in DMSO).

^*e*^Parameters of deprotonated form measured at pH 7.4.

^*f*^Parameters of protonated form measured at pH 2.4.

^*g*^Not detected.

#### 2.2.3. Reversibility and specificity of the pH dependent NIR fluorescence

The fluorescence responses of **IR1–4** were tested at pH 4.0 to evaluate the stability of the cyanine derivatives in acidic environments.^47^ Continuous exposure to a 750 nm laser (6.0 W cm^–2^) over one hour was applied to the probes. While both **IR2** and **IR3** displayed stable emission with less than 10% reduction in intensity, **IR1** and **IR4** were reduced by over 20%, which could be ascribed to intramolecular charge imbalance due to amine protonation at low pH (ref. 48) (Fig. S3A†). Being able to report on reversible physiological pH variations is crucial to dynamically monitoring therapeutic responses. As shown in Fig. S3B,† all the probes demonstrated full reversible fluorescence by switching the pH between 7.4 and 4.0 over a number of cycles. Notably, while above 90% fluorescence maxima of **IR2** and **IR3** were maintained after five cyclic pH switches, this value was below 80% for **IR1** and **IR4**. The above studies indicated that an increased photostability was observed for Hcyanine derivatives with unsymmetrical structures. Potential interferences from endogenous cations (K^+^, Ca^2+^, Mg^2+^, Zn^2+^, Co^2+^, Fe^3+^ and Fe^2+^) and amino acids (cysteine, proline, glycine and histidine) (200 mM) on the fluorescence response were also studied (Fig. S3C†). Only minor fluorescence changes (≤10%) were observed at pH 4.0 in the presence of all the above interfering substances except for Fe^3+^ (which gave a 35% signal reduction in the case of **IR1**). The Fe^3+^ ion may oxidize the unsaturated bonds in the Hcyanine and hence reduce its fluorescence. Considering the intracellular Fe^3+^ concentration (50–100 μM)^49^ is far below the tested concentration, its disruption to the probe signal should be minimized. The above data indicates that the pH dependent fluorescence responses of the probes are unlikely to be affected by endogenous molecules.

### 2.3. Theoretical calculations of the acidity triggered photophysical properties

**Fig. 4:**
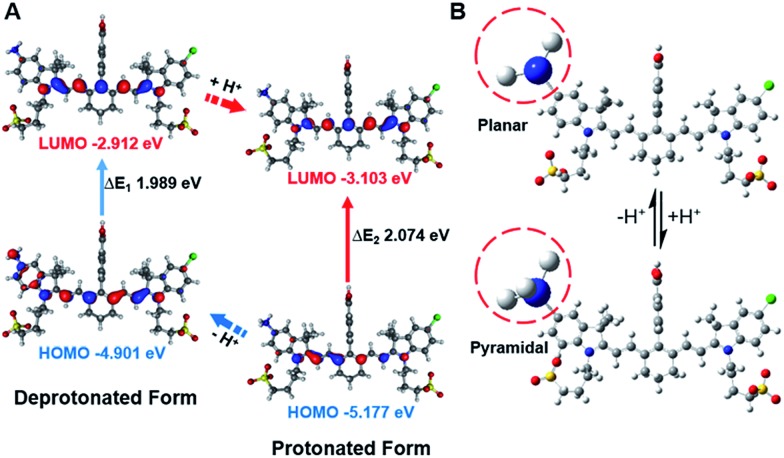
Frontier molecular orbital plots (A) and geometries (B) of **IR2** in deprotonated and protonated forms. The corresponding HOMO and LUMO energy levels and HOMO–LUMO energy gaps are indicated.

In order to study the mechanism of photophysical changes upon acidification, quantum chemistry calculations were carried out using Gaussian 09.^33^ The conformations of **IR1–4** in their deprotonated and protonated states were optimized by means of density functional theory (DFT) methods with the B3LYP/6-311G basis set levels prior to the population analysis of the frontier molecular orbitals (FMOs) of these compounds. In the deprotonated form, the HOMO localized both on the Hcyanine scaffold and bridgehead amine, whereas the LUMO only spread over the polymethine chain, indicating a significant charge transfer from the terminal amine to the polymethine upon excitation^44^ (Fig. 4A and S4†). In contrast, in the protonated form, electrons in the HOMO of the conjugated polymethine chain exhibited a large overlap with those in the LUMO. A more detailed conformational change of the bridgehead amine is highlighted in the optimized three-dimensional molecular structures in Fig. 4B. While it was demonstrated that a planar conformation (ICT state) existed in neutral environments, transformation into a pyramidal geometry (LE state) was observed after its quaternarization in acidic conditions.^50^ The transfer from the ICT to the LE state is often accompanied with an increased energy gap.^43^ The calculated energy gaps between the HOMO and LUMO increase by 0.109, 0.085, 0.108 and 0.211 eV (Δ*E*_gap_ = Δ*E*_1_ – Δ*E*_2_) for the protonation of **IR1–4** respectively. The increase in the energy gaps further explains the hypsochromic shift of absorption in acidic environments. Overall, the FMO analysis confirmed that **IR1–4** underwent ICT at neutral pH whereas this process was inhibited upon protonation of the amine moiety in acidic environments.

### 2.4. Theranostic probe for specifically imaging and killing cancer cells

#### 2.4.1. **IR2** showing low cytotoxicity to both cancer and normal cells

The cytotoxicity of **IR2** towards human hepatocellular carcinoma cell line HepG2, human adenocarcinoma cell line HeLa, normal human hepatic cell line HL-7702, human umbilical vein endothelium cell line HUVEC, and human embryonic kidney cell line HEK-293 was determined using a Cell Counting Kit-8 (CCK-8) assay (Fig. S5†). The relative viabilities of all five cell lines were above 80% even with a high probe concentration of 50 μM, which indicates a minimized acute cytotoxicity for this probe at the concentration used for imaging and therapeutic purposes.

#### 2.4.2. **IR2** showing time dependent cellular uptake

The intracellular fluorescence intensities of **IR2** in HepG2 cells were quantified at 0.5, 4, 12, and 24 h post-incubation (Fig. S6†). The confocal fluorescence images indicated that the overall **IR2** uptake increased proportionally within the first 12 h of incubation. Only a slight fluorescence enhancement was recorded when the incubation time was extended to 24 h. Therefore, 12 h was chosen as the incubation time for the subsequent *in vitro* microscopic experiments.

#### 2.4.3. **IR2** specifically illuminating cancer cells by signalling their acidic pH_lys_

**Fig. 5:**
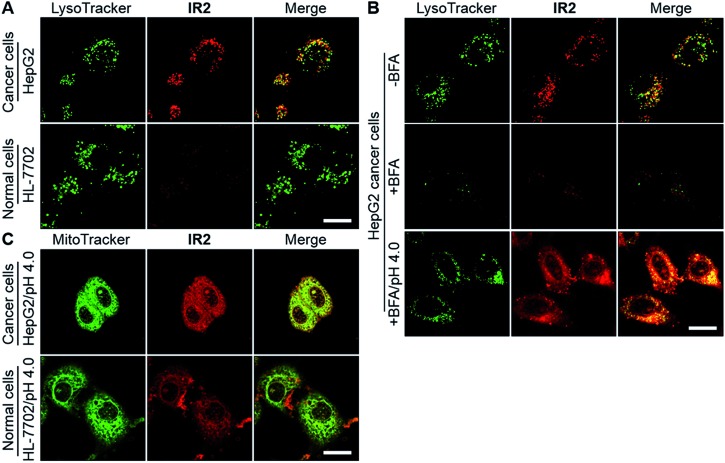
**IR2** specifically images cancer cells by sensing their acidic lysosomal lumen. (A) Confocal fluorescence microscopic images of live hepatic HepG2 cancer cells and hepatic HL-7702 normal cells at 12 h post incubation of **IR2** (20 μM). The fluorescence of **IR2** and the lysosomal marker are displayed in red and green respectively. (B) Fluorescence microscopic images of HepG2 cells after the treatment of **IR2** (20 μM) for 12 h in the absence or presence of bafilomycin A1 (BFA) (100 nM) or in the presence of BFA plus cytoplasmic acidification (pH 4.0). (C) Fluorescent microscopic images of HepG2 and HL-7702 cells at 12 h post incubation of **IR2** (20 μM) followed by suspension in acidic buffer (pH 4.0). The fluorescence of **IR2** and the mitochondrial marker are displayed in red and green respectively. Bar: 20 μm.

Confocal fluorescence microscopic imaging showed intracellular uptake of **IR2** as vesicular structures in both types of cancer cells. In contrast, very faint fluorescence signals were observed in normal cell lines including HL-7702, HUVEC, and HEK-293 (Fig. 5A and S7A†). For example, semi-quantitative studies demonstrated that the average intra-lysosomal signals of the HepG2 and HeLa cells were 2.8 and 2.7 times higher than that of HL-7702 cells (Fig. S7B†). Mander's coefficients of co-localization for fluorescence of **IR2** with respect to a lysosomal marker (LysoTracker Green DND-26) were determined to be 0.93 for HepG2, 0.82 for HeLa, 0.96 for HL-7702, 0.75 for HUVEC, and 0.84 for HEK-293 cells, which indicated the lysosomal delivery of the probe (Fig. S7C†). To verify the activation of **IR2** in the acidic lysosomal lumen, HepG2 cells were treated with bafilomycin A1 (BFA), a specific inhibitor of vacuolar-type H^+^-ATPase (V-ATPase) that maintains lysosomal acidity by actively pumping in protons.^53^ The lysosomal fluorescence reduced remarkably in the presence of BFA (100 nM, 2.0 h), which could be interpreted as the neutralization of lysosomal lumen (Fig. 5B). Notably, the intra-lysosomal fluorescence recovered partially when the BFA treated cells were cytoplasmically acidified by suspending them in a buffer solution at pH 4.0 for 10 min.^25^ The above studies not only demonstrated the delivery of **IR2** into lysosomes of both cancer and normal cells, but also verified that **IR2** specifically illuminated cancer cells by sensing their acidic pH_lys_.

#### 2.4.4. **IR2** showing higher mitochondrial uptake in cancer cells than normal cells

Even though the fluorescence signal of **IR2** remained “silent” in the mitochondria of all cell types (Mander's coefficients < 0.25) (Fig. S7D†), it was enhanced remarkably after suspension in an acidic buffer (pH 4.0) for 10 min (Fig. 5C and S7E†). Mander's coefficients of co-localization (the fraction of mitochondrial tracker, MitoTracker Green FM, overlapping with **IR2**) were determined to be 0.99 for HepG2, 0.88 for HeLa, 0.79 for HL-7702, 0.93 for HUVEC and 0.97 for HEK-293 cells after cytoplasmic acidification (Fig. S7F†). The above studies verified the mitochondrial delivery of this probe. Importantly, the fluorescence intensities of **IR2** in both HepG2 and HeLa cells after cytoplasmic acidification were 2.2- and 2.5-fold greater than that of HL-7702 cells, which implied the higher mitochondrial uptake of this probe in cancer cells than in normal cells. Additionally, the low uptake of **IR2** in normal cell lines was further confirmed in HUVEC and HEK-293 cells (Fig. S7G†). The efficient uptake of Hcyanine derivatives in cancer cells could be explained by the over-expressed OATPs that actively mediate the intracellular delivery of the heptamethine cyanine derivatives.^13^,^35^

#### 2.4.5. **IR2** selectively killing cancer cells *via* mitochondrial mediated cell death

**Fig. 6:**
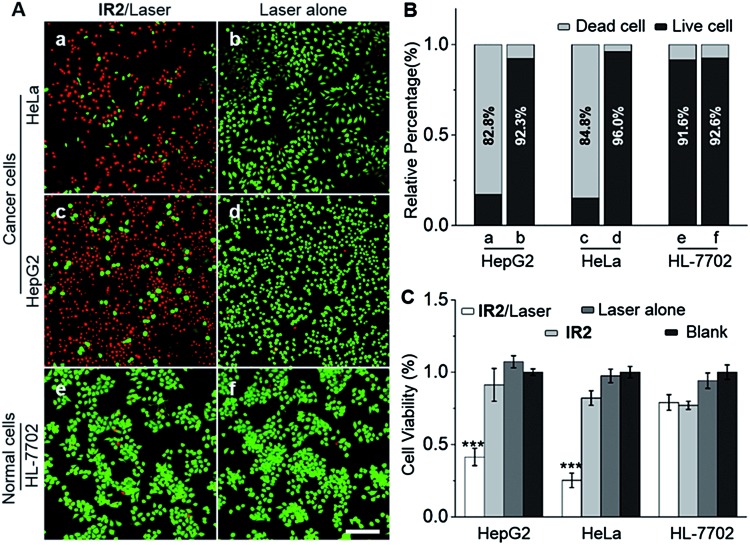
**IR2**/laser treatment selectively photoablates cancer cells. (A) Fluorescence microscopic images of HepG2, HeLa and HL-7702 cells stained with a calcein-AM/PI double staining kit. Cells were treated with either **IR2** (20 μM, 12 h)/laser (750 nm, 6.0 W cm^–2^, 10 min) or laser alone. Viable cells stained by calcein-AM are displayed in green and dead cells stained by PI are indicated in red. Bar: 200 μm. (B) The live and dead cell percentages quantified from double stained fluorescent images, *n* = 12 microscopic fields from three independent tests. (C) Cells were treated with **IR2** (20 μM, 12 h)/laser (750 nm, 6.0 W cm^–2^, 10 min), **IR2** alone, laser alone or blank. Relative cell viabilities were measured by CCK8 assay at 24 h post treatment. The bars are shown as mean ± standard deviation (SD), *n* = 5 wells for each group. ***P < 0.001 *vs.* blank.

**Fig. 7:**
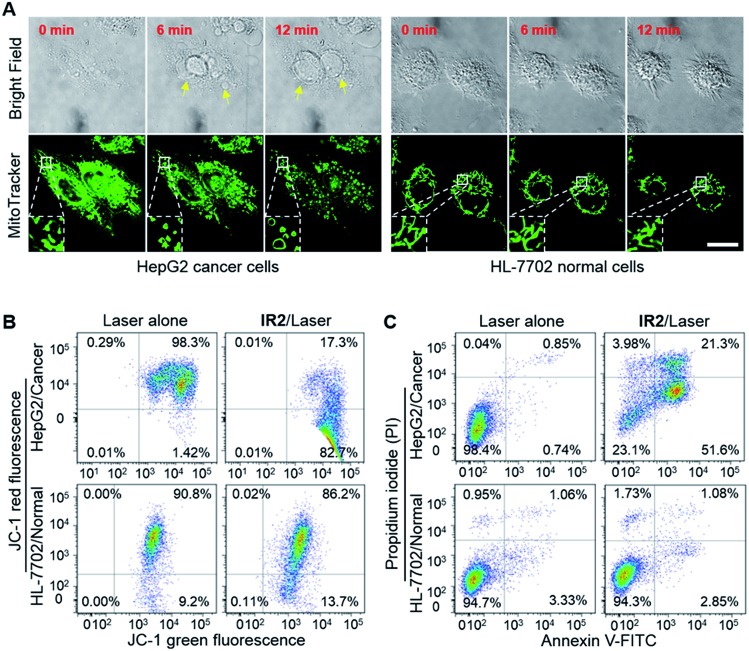
**IR2**/laser treatment disrupts mitochondrial membrane and initiates cancer cell death. (A) Time lapse bright field and fluorescence images of live HepG2 and HL-7702 cells upon laser irradiation (633 nm, 5 mW) after **IR2** incubation for 12 h. The fluorescence of the mitochondrial markers are displayed in green. Arrows point to membrane blebbing. Insets: enlarged view of mitochondrial morphology variations. Bar: 20 μm. (B) Flow cytometric analysis of JC-1 dye stained HepG2 and HL-7702 cells after **IR2** (20 μM, 12 h)/laser irradiation (750 nm, 6.0 W cm^–2^, 10 min) treatment or laser alone. Changes in the red/green fluorescence ratio indicate mitochondrial membrane disruption. (C) Flow cytometric analysis of annexin V/PI double stained cells after **IR2**/laser treatment. Percentages of live (PI–/annexin V–), early apoptotic (PI–/annexin V+), late apoptotic (PI+/annexin V+) and necrotic (PI+/annexin V–) cells are indicated in each quadrant.

HepG2 and HeLa cells were employed as models for cancer cells whilst HL-7702 cells were used as a model for normal cells to study the photocytotoxicity of **IR2***in vitro*. The combined treatment of **IR2** (20 μM, 12 h incubation) and 750 nm laser irradiation (6.0 W cm^–2^, 10 min) rapidly changed the morphology of both HepG2 and HeLa cancer cells from a stereo spindle shape into a plane shrunken shape (Fig. S8†). In contrast, HL-7702 normal cells maintained their original polygonal shape after the same treatment. The selective phototoxicity of **IR2** to cancer cells was evaluated by calcein-AM/propidium iodide (PI) double staining that simultaneously identified viable cells stained by calcein-AM in green and dead cells stained by PI in red (Fig. 6A). While 82.8% HepG2 cells and 84.8% HeLa cells were dead after the above combined treatment, this percentage was measured to be as low as 9.4% for HL-7702 cells (Fig. 6B). The CCK8 assay further verified the selective phototoxicity of **IR2** for the cancer cells. While the viabilities of HepG2 and HeLa cells remained as low as 41.3% and 25.2% respectively after the combined treatment, the viability remained above 80.0% for HL-7702 cells (Fig. 6C). Similarly, much higher survival rates (around 70%) were detected in other normal cell lines including HUVEC and HEK-293 (Fig. S7H†). Compared to the calcein-AM/PI double staining results, the relatively higher survival rates of the cancer cells determined by the CCK8 assay are explained by the existence of late apoptotic cells that could be identified by CCK8 but not by calcein-AM staining. To confirm that photothermal rather than photodynamic effects were the dominant mechanism for cancer cell death, the reactive oxygen species (ROS) levels generated by the **IR2**/laser irradiation were evaluated both in the buffer solutions and within the cells (Fig. S9†). The **IR2**/laser treatment could generate, to a certain extent, ROS at neutral pH. However, it should not be the dominant factor leading to cell death *in vitro*. To elucidate the mechanism of **IR2** induced selective cancer cell death *via* hyperthermia, fluorescence microscopic studies were conducted in live cells. Even though continuous laser irradiation (633 nm, 5 mW, 12 min) under a confocal microscope led to a morphological shrinkage of both cancer and normal cells, only cancer cells suffered a loss of membrane integrity accompanied by extensive and irreversible membrane blebbing, a generally accepted sign of the onset of apoptosis^55^ (Fig. 7A and S10A†). Interestingly, while the strip-like structure of the mitochondria in normal cells remained intact after laser exposure, the mitochondria in both types of cancer cells changed significantly with the formation of discernible vascular-swollen structures (Fig. 7A and S10A†). Similar mitochondrial morphology variations were also observed in reported works which relate to mitochondrial cristae remodelling and mitochondrial outer membrane permeabilization.^56^,^57^ Membrane blebbing and swelling of mitochondria are the common morphological effects induced by hyperthermia,^52^ which further supports our hypothesis that hyperthermia is the predominant factor initiating cell death. To elucidate whether **IR2** photoablated cancer cells by disrupting their mitochondria, the remaining living cells after the combined **IR2**/laser treatment were stained by JC-1 dye. JC-1 dye is commonly used to determine mitochondrial membrane potential, a key parameter representing mitochondrial integrity. Due to the electrostatic attraction, specific accumulation of JC-1 in mitochondria leads to the fluorescence emission shifting from green (monomers) to red (J-aggregates).^58^ The increase of the green/red fluorescence intensity ratio indicates mitochondrial depolarization and disruption. After the combined treatment of **IR2**/laser, the JC-1 fluorescence ratio of HepG2 and HeLa cells increased from 0.01 to 4.78 and 0.21 respectively (Fig. 7B and S10B†). In contrast, the ratio for HL-7702 cells barely increased. The above studies indicate selective mitochondrial membrane disruption in cancer cells after **IR2**/laser treatment. The passage of ions and small molecules through the mitochondrial membrane results in decoupling of the respiratory chain and cytoplasmic leakage of pro-apoptotic proteins such as cytochrome c, which initiates “point of no return” apoptotic or necrotic cascades.^59^ The cell death pathways were further studied by flow cytometry after staining with an annexin V-FITC/PI kit. While annexin V labels apoptotic cells by binding phospholipid phosphatidylserine that is translocated on the membrane surface, PI stains the cellular DNA in dead/late necrotic cells where the membrane has been destroyed.^58^Fig. 7C and S10C† show the percentages of viable (annexin V–, PI–), early apoptotic (annexin V+, PI–), late apoptotic (annexin V+, PI+) and necrotic (annexin V–, PI+) cells after the treatment of **IR2**/laser or laser irradiation alone. The combined treatment induced cancer cell apoptosis, which was evidenced by the high population percentages of early apoptotic cells (51.6% for HepG2 and 84.5% for HeLa) and late apoptotic cells (21.3% for HepG2 and 8.34% for HeLa). Remarkably, the apoptotic populations of the normal HL-7702 cells were barely increased after the above treatment. Compared to the normal cells, a higher probe uptake in mitochondria^35^ and sensitivity to heat shock^51^ may jointly lead to the specificity of **IR2**/laser irradiation in killing cancer cells. Additionally, the high photocytotoxicity of **IR2** to both hepatocellular hepG2 cancer cells and adenocarcinoma HeLa cells implies the potential applicability of this theranostic probe to eliminate tumors by sensing their abnormal intra-organellar pHs.

#### 2.4.6. Outlook for **IR2** on *in vivo* imaging and targeted ablation of cancer cells

Hcyanine derivatives are widely reported for tumor imaging and treatment *in vivo*. For example, ICG has been utilized clinically in visualizing hepatocellular carcinomas and colorectal hepatic metastases with high sensitivity.^6^ Additionally, Hcyanine derivatives such as IR-780, IR-783 and IR-808 also showed preferential uptake in cancer cells *via* over-expressed OATPs under *in vivo* conditions.^35^,^54^,^60^ Therefore, **IR2** holds promise for imaging and ablating tumor foci *in vivo* without conjugation to any receptor targeting ligands. Furthermore, **IR2** showed minimized cytotoxicity in both cultured human cancer and normal cells at photothermally active doses, which is especially important for its clinical translation. Given the presumably short circulation lifetime of **IR2***in vivo*, the rapid clearance of this probe and its exclusive activation in the lysosomes of cancer cells, **IR2** will visualize the tumor foci with the “bright stars in black sky” effect. Ongoing *in vivo* studies of **IR2** will be reported in our subsequent works.

## 3. Conclusion

Overall, we developed four Hcyanine based theranostic probes showing pH switchable NIR fluorescence and photothermal efficiency under physiological environments. By fine-tuning the p*K*a values of these probes, **IR2**, with a p*K*a_fluo_ value of 4.6, specifically visualized and eradicated multiple types of cancer cells by maximizing its NIR fluorescence intensity in acidic lysosomal lumen and its photothermal effects in the alkaline mitochondrial matrix. Considering that a disordered organellar pH is a common characteristic of cancer cells regardless of their genotypes or phenotypes, these theranostic probes hold promise in assisting image-guided tumor photoablation by completely eradicating tumor foci and improving prognosis.

## Supplementary Material

Supplementary information
