# Supplementary material for: Selective imaging and cancer cell death via pH switchable near-infrared fluorescence and photothermal effects
Source: Chem Sci. 2016 May 26;7(9):5995–6005. doi: 10.1039/c6sc00221h (PMC6022192; doi:10.1039/c6sc00221h)
Supplement: Supplementary file 1 [file SC-007-C6SC00221H-s001.pdf]

Electronic Supplementary Material (ESI) for Chemical Science.
This journal is © The Royal Society of Chemistry 2016

# Selective imaging and cancer cell death via pH switchable near-infrared fluorescence and photothermal effects

Jingye Zhang[a], Zining Liu[a], Peng Lian[a], Jun Qian[a], Xinwei Li[a], Lu Wang[c], Wei Fu[a], Liang Chen[d], Xunbin Wei[b,*], and Cong Li[a,*]

[a]Key Laboratory of Smart Drug Delivery, Ministry of Education, School of Pharmacy, Fudan University, Shanghai 201203, China

[b]State Key Laboratory of Oncogenes and Related Genes, Shanghai Cancer Institute, School of Biomedical Engineering, Shanghai Jiao Tong University, 1954 Huashan Road, Shanghai 200030, China

[c]Department of Chemistry, National University of Singapore, Singapore 117543, Singapore

[d]Department of Neurosurgery, Huashan Hospital, Fudan University, Shanghai 200040, China.

# Supporting Information

## <u>Table of content</u>


1. Supplemental Figures and Tables S3–11

2. General Materials and Instruments S12

3. Synthesis and Characterization S13–18

4. Photospectroscopic Studies S19–21

5. Microscopic and Flow Cytometric Studies S22–24

6. Supplemental Spectra S25–44

7. Reference S45

## 1. Supplemental Figures and Tables

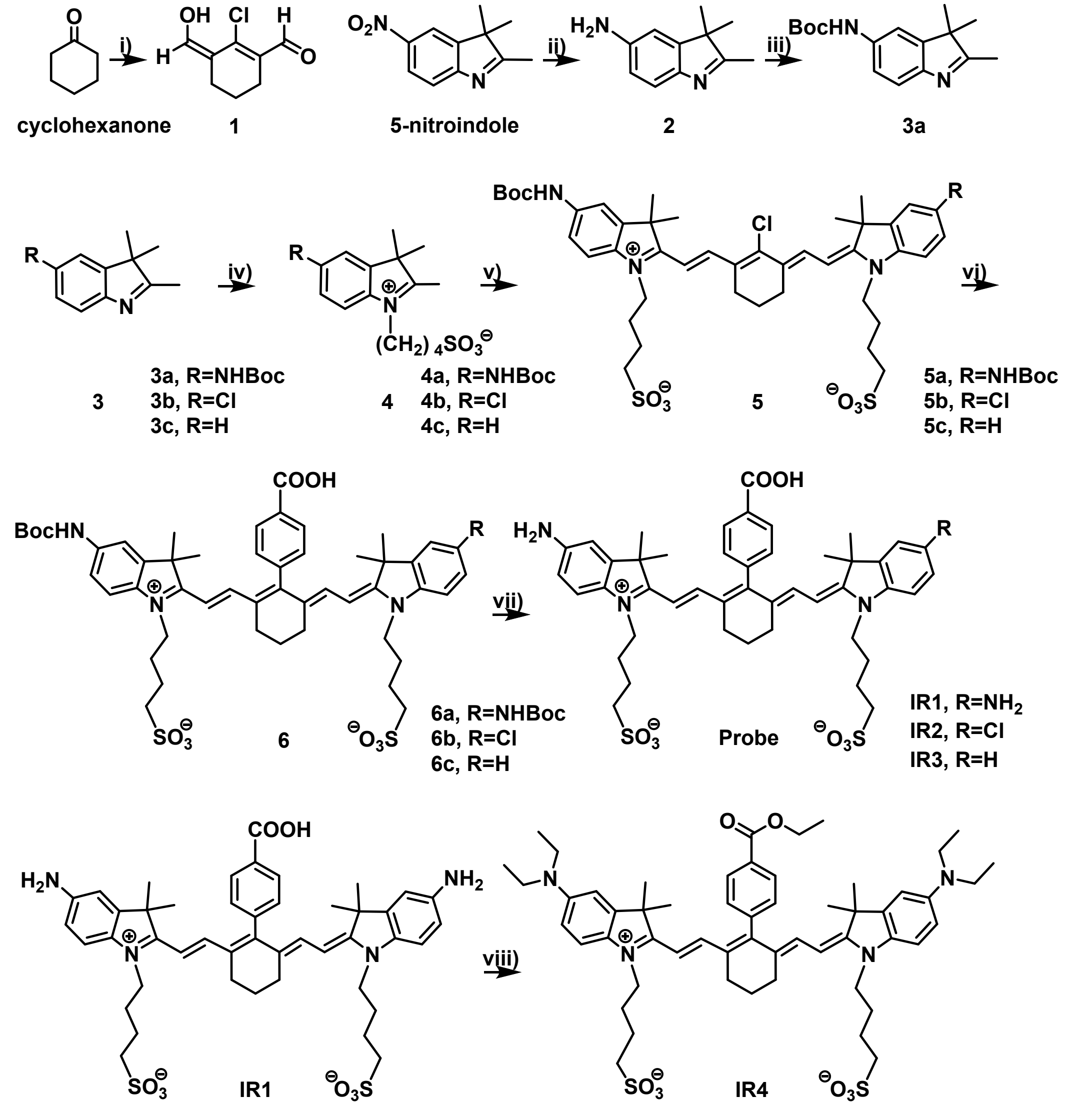


**Scheme S1.** Synthesis of pH responsive NIR theranostic probes **IR1**–**4**. (i) $DMF/POCl_3/CH_2Cl_2$; (ii) $SnCl_2/HCl$; (iii) $(Boc)_2O/NaHCO_3$/Dioxane/$H_2O$; (iv) 1,4-butane sultone, 1,2-dichlorobenzene; (v) **1**,**3a**, $(Ac)_2O$; (vi) 4-carboxyphenylboronic acid, $Pd(pph_3)_4/H_2O$; (vii) $CH_2Cl_2$/TFA; (viii) $CH_3CH_2Br$/DIPEA/DMF.

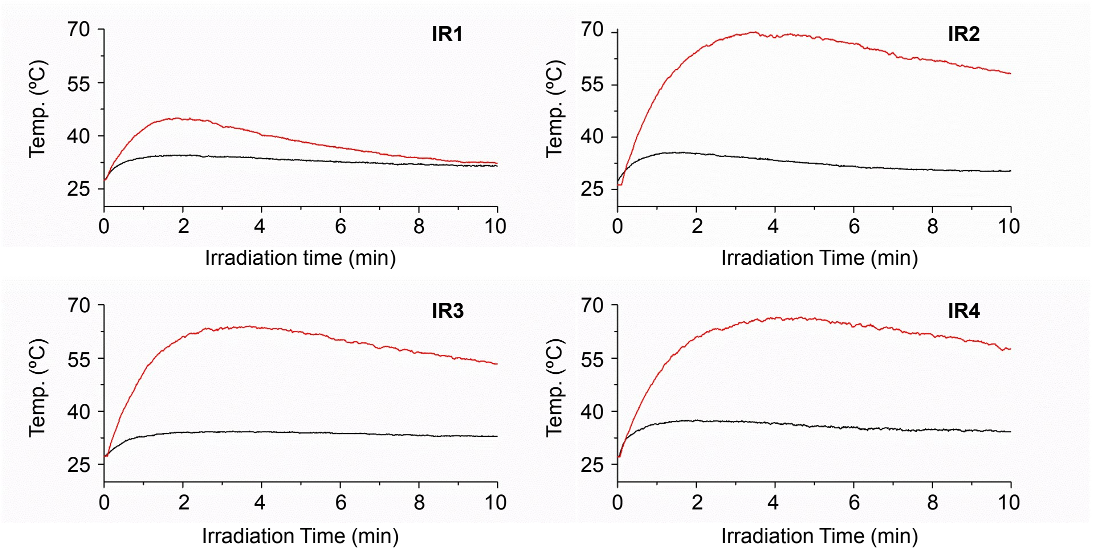


**Figure S1.** Temperature profiles of **IR1–4** (10 μM) as a function of laser irradiation time (750 nm, 6.0 W/cm²) in acidic (pH 4.0, black line) and neutral (pH 7.4, red line) buffer solutions.

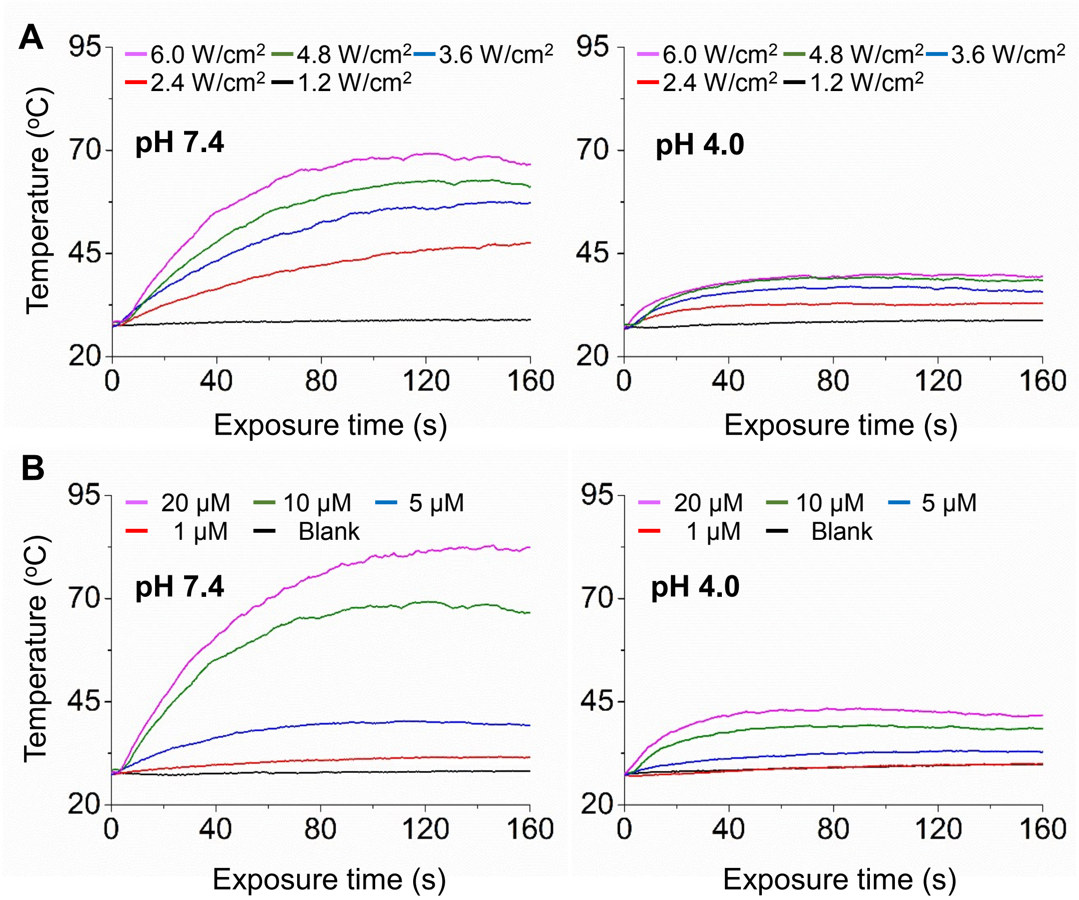

**Figure S2.** **IR2** showed concentration and laser power dependent photothermal efficiencies in both neutral and acidic environment. (A) Laser exposure time dependent temperature increase profiles of **IR2** (10 μM) in buffered solutions (pH 7.4 and 4.0) under selected laser powers (1.2, 2.4, 3.6, 4.8 and 6.0 W/cm$^2$). (B) Laser irradiation time dependent temperature increase profiles of **IR2** in buffered solutions (pH 7.4 and 4.0) as a function of its concentrations (0, 1, 5, 10, 20 μM).

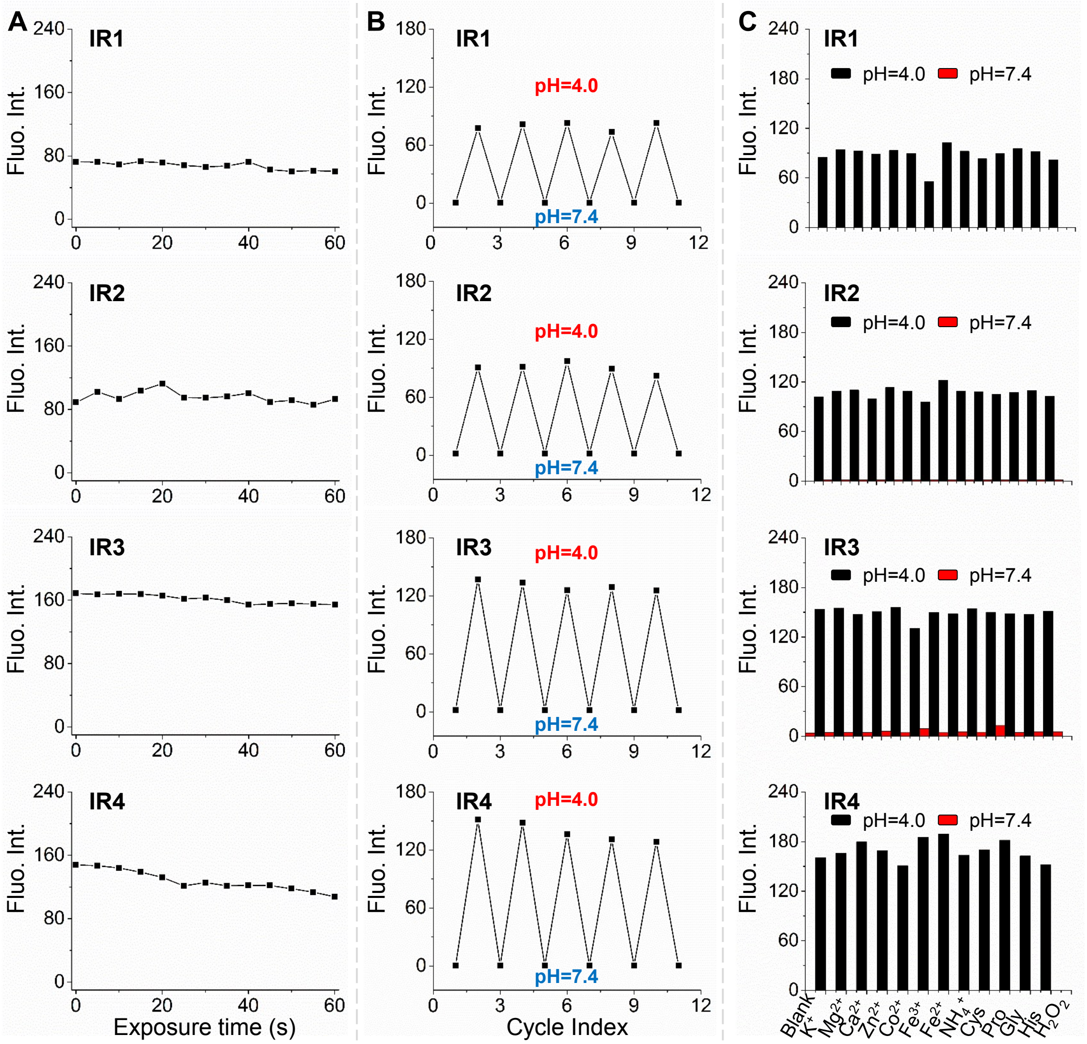


**Figure S3.** Photophysical stability of **IR1–4** (1.0 μM, $\lambda_{ex}$ = 750 nm) in buffered solutions containing 0.1% DMSO. (A) Fluorescence intensities of **IR1–4** under pH 4.0 as a function of incubation time. Fluorescence intensities were measured every 5 min for 1 h. (B) Fluorescence intensities of **IR1–4** with pH reversibly changed between 4.0 and 7.4 for five rounds. (C) Fluorescent intensities of **IR1–4** at pH 4.0 and 7.4 in the presence of different types of metal ions and amino acids (200 mM) respectively.

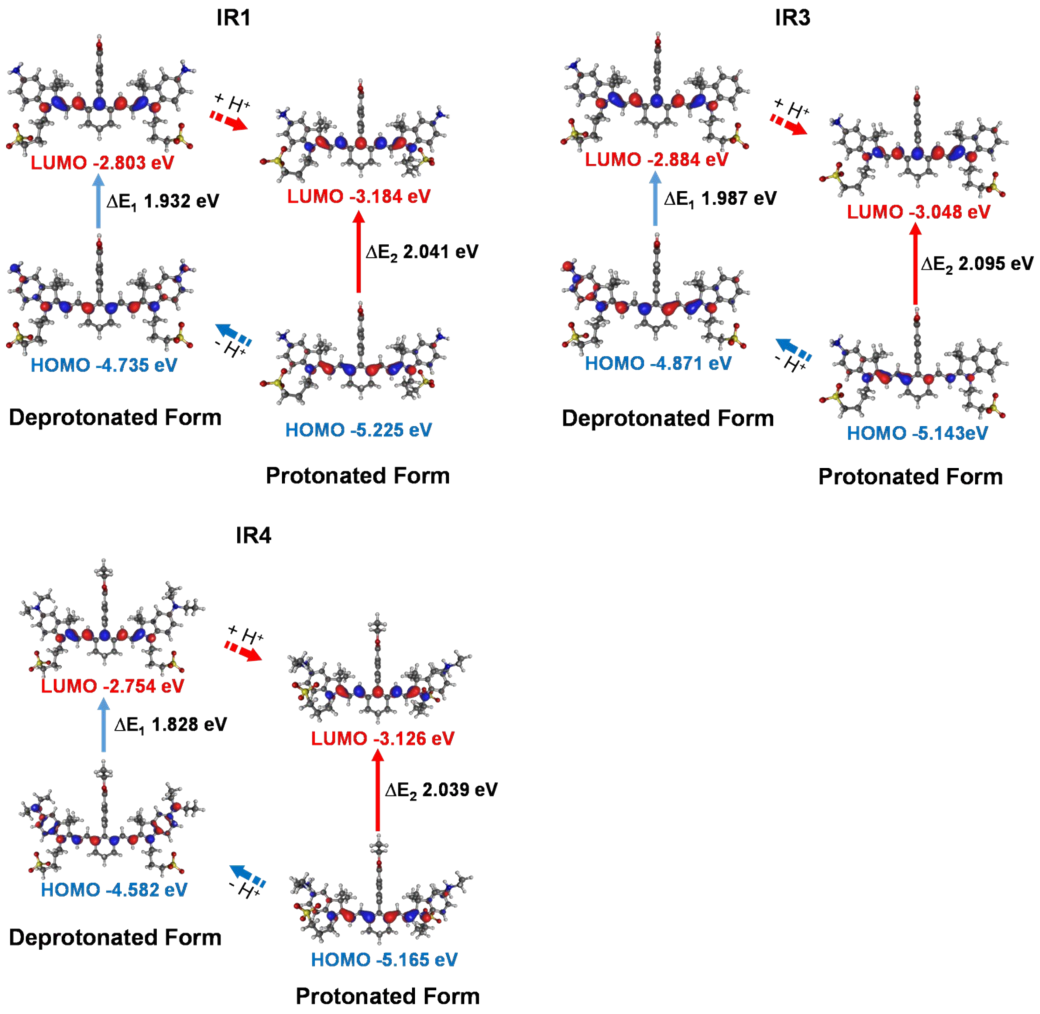


**Figure S4.** Frontier molecular orbital plots of **IR1**, **IR3** and **IR4** in their deprotonated and protonated forms. The corresponding HOMO, LUMO energy levels and HOMO–LUMO energy gaps are indicated.

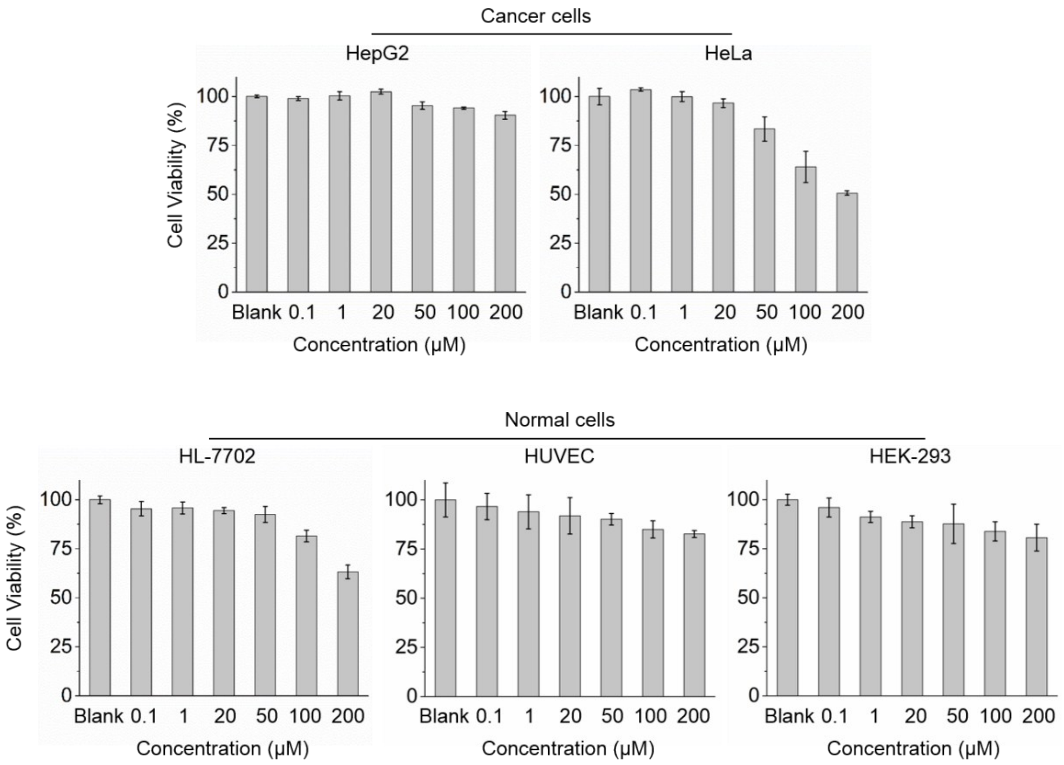


**Figure S5.** **IR2** showed minimized cytotoxicity in both cancer and normal cells. Concentration dependent cytotoxicities of **IR2** in HepG2, HeLa, HL-7702, HUVEC, and HEK-293 cells after incubation for 24 h. The relative cell viabilities were determined by CCK-8 assay. The data were presented as mean ± SD, n = 5 wells of 96-well plates for each group.

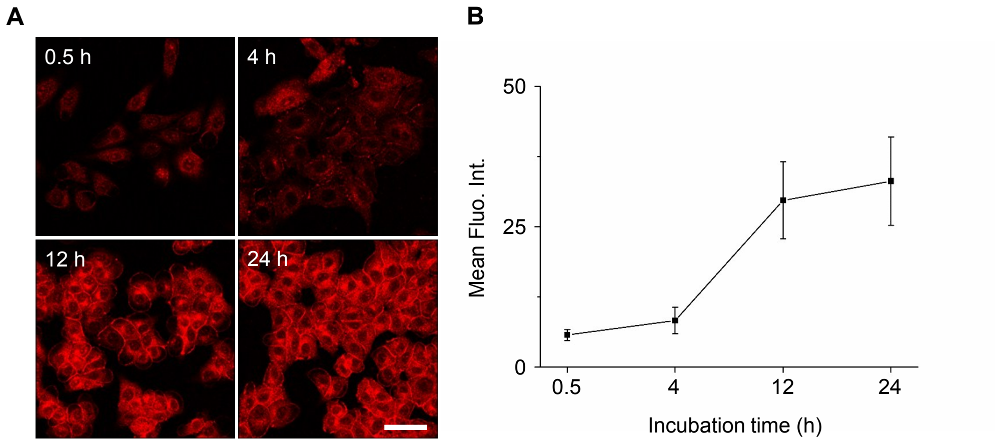


**Figure S6.** **IR2** showed time-dependent cellular uptake. (A) Fluorescent microscopic images of live HepG2 cells at selected time (0.5, 4, 12, 24 h) post incubation of **IR2** (20 μM). The cells were perfused in buffer solution (pH 4.0) for 10 min prior imaging and **IR2** fluorescence was displayed in red. Bar, 50 μm. (B) Mean fluorescence intensities of **IR2**

(20 μM) in HepG2 cells were quantified at selected time point post-incubation (n = 5 microscopic fields). All error bars are s.e.m.

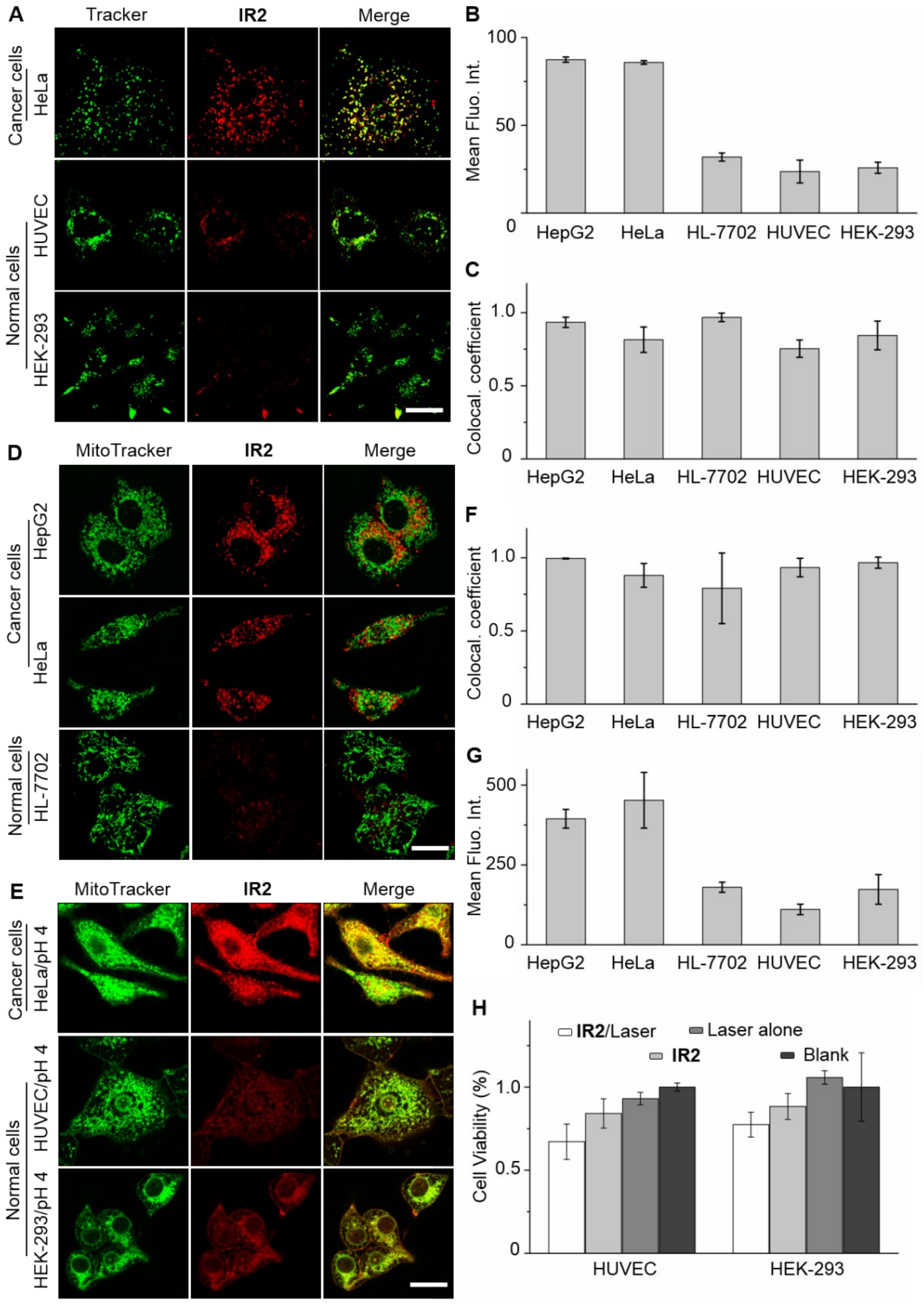


**Figure S7.** **IR2** specifically imaged cancer cells by sensing their acidic lysosomal lumen. (A) Confocal fluorescence microscopic images HeLa, HUVEC and HEK-293 cells at 12 h

post incubation of **IR2** (20 μM). NIR fluorescence were displayed in red and fluorescence of lysosomal or mitochondrial marker was displayed in green. (B) Mean fluorescence intensities of **IR2** (20 μM, 12 h) in live HepG2, HeLa, HL-7702, HUVEC, and HEK-293 cells quantified from microscopic images. The bars were shown as mean ± SD, n = 12 microscopic fields from three independent tests for each value. (C) Mander's coefficients of co-localization for **IR2** with respect to lysotracker quantified from confocal microscopic images. The bars were shown as mean ± SD, n = 5 microscopic fields for each value. (D) Fluorescence microscopic images of live HepG2, HeLa and HL-7702 cells stained with **IR2** (20 μM, 12 h) and mitochondrial marker. NIR fluorescence were displayed in red and mitochondrial fluorescence were displayed in green. Bar, 20 μm. (E) Confocal fluorescence microscopic images of HeLa, HUVEC, and HEK-293 cells at 12 h post incubation of **IR2** (20 μM). NIR fluorescence were displayed in red and fluorescence of mitochondrial marker were displayed in green. Cells were suspended in acidic buffer (pH 4.0) for 10 min before imaging. Bar, 20 μm. (F) The colocalization coefficients between **IR2** and mitochondrial marker under intra-plasmatic acidification (pH 4.0). The bars were shown as mean ± SD, n = 5 microscopic fields for each value. (G) Mean fluorescence intensities of **IR2** (20 μM, 12 h) in HepG2, HeLa, HL-7702, HUVEC, and HEK-293 cells quantified from microscopic images after cytoplasmic acidification. The bars were shown as mean ± SD, n = 12 microscopic fields from three independent tests for each value. (H) Cells were treated with **IR2** (20 μM, 12 h)/laser (750 nm, 6.0 $W/cm^2$, 10 min), **IR2** alone, laser alone or blank. Relative cell viabilities were measured by CCK8 assay at 24 h post treatment. The bars were shown as mean ± standard deviation (SD), n = 5 wells for each group.

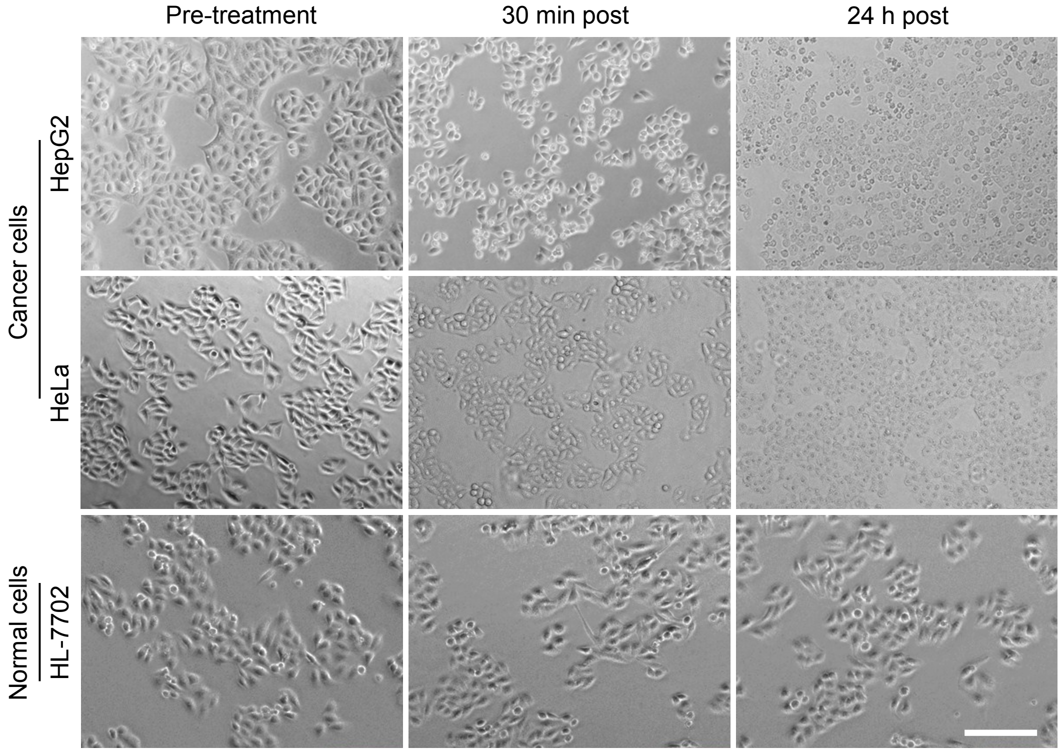


**Figure S8.** **IR2**/laser irradiation led to obvious morphological changes in cancer cells but barely in normal cells. White light microscopic images of cells at selective time points after combined treatment of **IR2** (20 μM, 12 h) and laser irradiation (750 nm, 6.0 W/cm$^2$, 10 min). Bar, 200 μm.

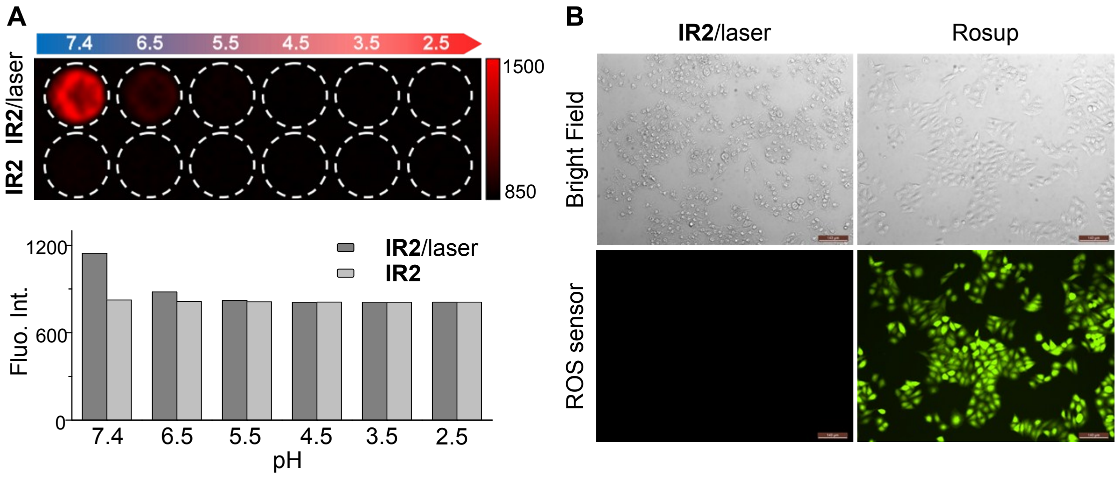


**Figure S9.** **IR2**/laser irradiation increased ROS level slightly. (A) Fluorescent images of ROS sensor (5.0 μM) in buffer solution in the presence or absence of 10 μM **IR2** at 10 min post irradiation of 750 nm laser (6.0 W/cm$^2$). (B) White light and fluorescent microscopic images of HepG2 cancer cells after the treatment of **IR2** (20 μM, 12 h)/laser irradiation (750

nm, 6.0 W/cm$^2$, 10 min) or ROSUP (1.0 μL/mL, 30 min) as a positive control. ROS sensor (10 μM) was loaded 30 min prior imaging and displayed in green. Bar, 140 μm.

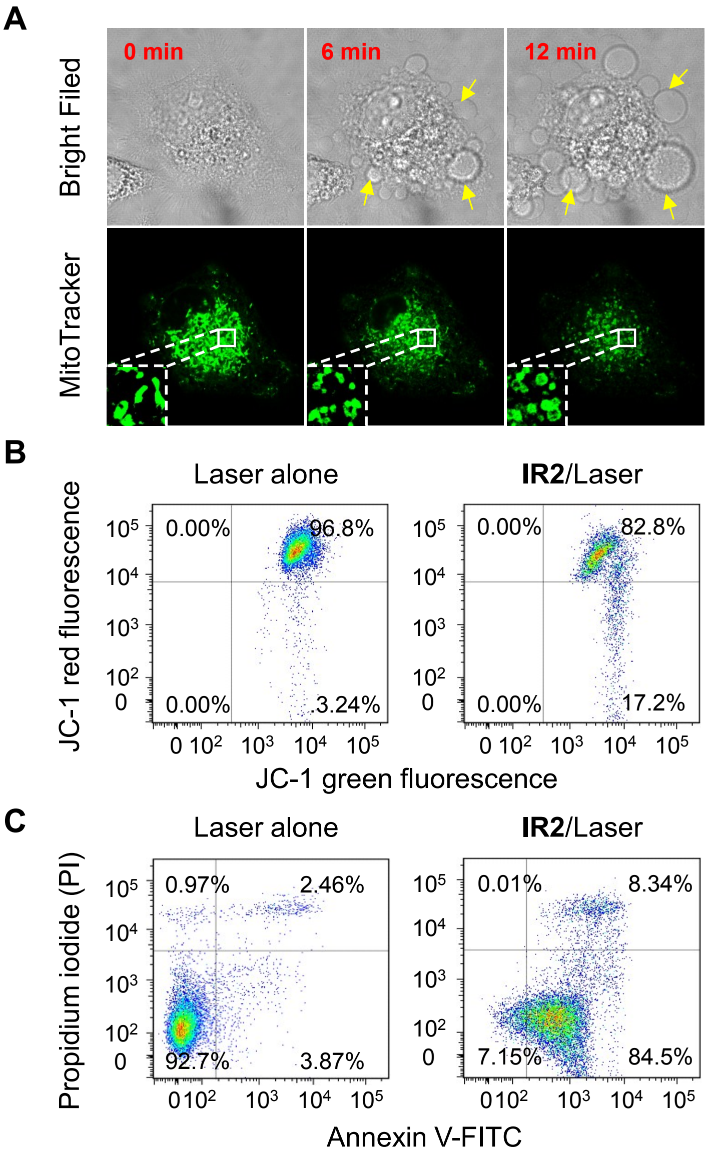


**Figure S10.** **IR2**/laser treatment disrupted mitochondrial membrane and initiated cancer cell death. (A) Time lapse bright field and fluorescence images of HeLa cells upon laser irradiation (633 nm, 5 mW) after **IR2** incubation for 12 h. The fluorescence of mitochondrial marker were displayed in green. Arrows point to membrane blebbing. Inserts: enlarged view of mitochondrial morphology variations. Bar, 20 μm. (B) Flow cytometric analysis of JC-1 dye stained HeLa cells after **IR2** (20 μM, 12 h)/laser irradiation (750 nm, 6.0 W/cm$^2$, 10 min) treatment or laser alone. Changes in the red/green fluorescence ratio indicated the mitochondrial membrane disruption. (C) Flow cytometric analysis of Annexin V/PI double stained HeLa cells after **IR2**/laser treatment. Percentages of live (PI-/Annexin V-), early

apoptotic (PI-/Annexin V+), late apoptotic (PI+/Annexin V+) and dead/necrotic (PI+/Annexin V-) cells were indicated in each quadrant.

## 2. General Materials and Instruments

All chemical reagents and solvents for probe synthesis were obtained from Aladdin Reagent (China) unless otherwise specified. Thin layer chromatography was recorded on alumina based silica gel plates (Merck, Germany). 2,3,3-trimethyl-3H-indolium, 5-chlorine-2,3,3-trimethyl-3H-indolium and 5-Nitro-2,3,3-tetramethyl-3H-indolium were purchased from Beijing Chengyu Specialty Chemical Co. Ltd. (China). $^{1}H$ and $^{13}C$ NMR spectra were recorded on a 400 MHz (Varian, USA) or 600 MHz (Bruker, USA) NMR spectrometer. High resolution electron spray ionization (HR-ESI) mass spectra were obtained on Q-TOF 2 (Micromass, USA) or AB 5600+ Q-TOF mass spectrometer (AB Sciex, USA). High performance liquid chromatography (HPLC) analysis were conducted on Agilent (USA) 1100 HPLC system equipped with G1315B Diode Array Detector (DAD) and DAISO (Japan) SP-300-5-ODS-BIO analytical column (4.6 mm × 200 mm). All pH measurements were performed with a Mettler Toledo (Switzerland) MP220 pH meter. Absorption spectra were performed on a SHIMADZU (Japan) UV-2550 spectrophotometer. Fluorescence spectra were collected on a SHIMAZDU (Japan) RF-5301PC fluorophotometer. In vitro fluorescence images were acquired in IVIS Spectrum In Vivo Imaging System (Caliper Life Sciences, USA). The MDL-III-750nm-2W laser was obtained from Changchun New Industries Optoelectronics Tech. Co. Ltd. (China). The thermal profiles were determined with an A150-15-M infrared thermal camera (IRtech Ltd. China). Fluorescence microscopic images were performed from a Leica (Germany) DMI4000D inverted microscope or a Zeiss (Germany) LSM 710 or 880 confocal microscope. Flow cytometric studies were analyzed on BD (USA) FACSAria II flow cytometer. Dulbecco's Modified Eagle Medium (DMEM), Roswell Park Memorial Institute 1640 (RPMI 1640) and GIBCO fetal bovine serum (FBS) were purchased from Thermo Fisher Scientific (USA). LysoTracker Green DND-26 and MitoTracker Green FM were purchased from Invitrogen (USA). Cell Counting Kit-8 (CCK-8), JC-1 Mitochondrial Membrane Potential Assay Kit, and Annexin V-FITC/PI Apoptosis Detection Kit were supplied by Yeasen (China). Reactive Oxygen Species (ROS) Assay Kit were purchased from Beyotime (China). Human hepatic cancer cell line HepG2, human cervical cancer cell line HeLa, and human embryonic kidney cell line HEK-293 were purchased from ATCC (USA). The normal human hepatic cell line HL-7702 was purchased from the Cell Bank of the Chinese Academy of Sciences (China). Primary human umbilical vein endothelial cell line HUVEC were purchased from Cascade Biologics (Eugene, USA).

## 3. Synthesis and Characterization

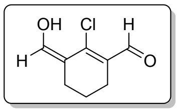


**Compound 1.** A mixture of 40 mL dimethylformamide (0.5 mol) and 40 mL methylene chloride was chilled in an ice bath for 30 min. 37 mL phosphorus oxychloride (0.4 mol) and cyclohexanone (10 g, 0.1 mol) were added dropwise to the mixture with stirring. The solution was refluxed for 3 h, cooled to room temperature, poured onto 200 g of ice, and allowed to stand overnight. The yellow solid was collected and weighted 8.0 g (46.5%).

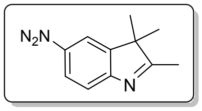


**Compound 2.** 5-nitro-2,3,3-trimethyl-3H-indolium (205 mg, 1.0 mmol) was added to a solution of stannous chloride (1.6 g, 7.0 mmol) in hydrochloric acid (32%, 10 mL) with stirring. The mixture was stirred for 16 hours and allowed to cool to room temperature. The reaction mixture was poured onto crushed ice and the solution was rendered alkaline with sodium hydroxide (5.0 M). The mixture was filtered and dissolved in chloromethane and washed with water (3 x 10 mL). The organic phase was dried over anhydrous sodium sulfate and decolorized with a small amount of active carbon. The solvent was removed by rotary evaporation and the targeted compound was obtained as light yellow solid (128.2 mg 73.2%). $^{1}$H NMR (400 MHz, CD3OD) δ 7.15 (d, J = 8.2 Hz, 1H), 6.74 (d, J = 2.1 Hz, 1H), 6.63 (dd, J = 8.2, 2.2 Hz, 1H), 2.20 (s, 3H), 1.26 (s, 6H). $^{13}$C NMR (101 MHz, $CDCl_3$) δ 186.18 (C), 148.29 (C), 147.30 (C), 145.47 (C), 120.29 (CH), 115.23 (CH), 110.43 (CH), 54.49 (C), 23.55 (2×$CH_3$), 14.86 ($CH_3$). TOF-MS calculated for $C_{11}H_{15}N_2^+$ [MH$^+$]: 175.1235 (100%), found 175.1227.

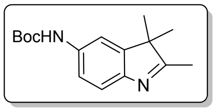


**Compound 3a.** Compound **2** (175.1 mg, 1.0 mmol), di-*tert*-butyl dicarbonate (261.9 mg, 1.2 mmol) and sodium bicarbonate (168.0 mg, 2.0 mmol) were added into a mixture of distilled water and dioxane (V:V = 1:1) in an ice-bath and stirred for 45 min. The reaction bottle was then heated and refluxed under argon for another 12 h and the solvent was removed. The crude product was purified by silica gel based column chromatography with an eluting solvent (petroleum ether : ethyl acetate = 1:1) to offer compound **3a** as a light yellow powder (226.1 mg, 82.2%). $^{1}$H NMR (400 MHz, DMSO) δ 7.56 (s, 1H), 7.31 (d, J = 8.3 Hz, 1H), 7.23 (dd, J = 8.3, 2.0 Hz, 1H), 2.25 (s, 3H), 1.52 (s, 9H), 1.30 (s, 6H). $^{13}$C NMR (101 MHz, CDCl3) δ 189.29 (C), 155.32 (C), 148.78 (C), 147.60 (C), 138.70 (C), 119.97 (CH), 119.09 (CH), 113.84 (CH), 80.85 (C), 55.02 (C), 28.75 (3×$CH_3$), 23.41 (2×$CH_3$), 15.12 ($CH_3$). TOF-MS calculated for $C_{16}H_{23}N_2O_2^+$ [MH$^+$]: 275.1760 (100%), found 275.1746.

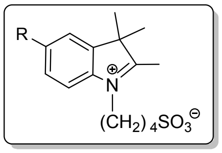


**Compound 4a–4c.** General synthetic procedure of compound **4a**–**4c**. Compound **3a**–**3c** (1.0 mmol), 1,4-Butane sultone (1.1 g, 8.0 mmol) and 2.0 mL 1,2-dichlorobenzene were mixed and heated at 110 ºC with stirring for 12 h under argon. At the end of reaction, the solution was cooled to room temperature and dropped into methyl ether in an ice bath. After precipitation,

the red product was filtered and re-dissolved in 20 mL saturated sodium chloride and was extracted with chloroform (3 x 20 mL). After that, the water extract was evaporated and the product was dried under vacuum without further purification. The yields of **4a**–**4c** were determined in a range of 46-75%.

**Compound 5a**–**5c.** General synthetic procedure of compound **5a**–**5c**. Compound **4a**–**4c** (1.1 mmol), sodium acetate (82.1 mg, 1.0 mmol) and 172 mg (1.0 mmol) of compound **1** were dissolved in 6.0 mL acetic anhydride in a flask. Another portion of compound **4a** (410.1 mg, 1.0 mmol) was dissolved in 3.0 mL acetic anhydride and then added dropwise to the above mixture under argon. The reaction mixture was heated to 70 °C with constant stirring for 45 min. Above mixture was cooled to room temperature and then dropped into the methyl ether in an ice bath and rude green powder was filtrated after precipitation. The crude was purified by column chromatography on silica gel with a mixture of $CH_2Cl_2$ and $CH_3OH$ as eluent. Removal of solvent under vacuum afforded pure deep green powder.

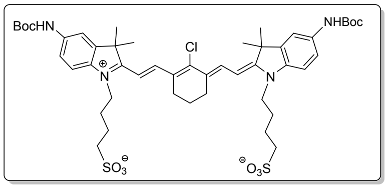


**Characterization of 5a.** (yield: 73.4%) $^{1}$H NMR (400 MHz, MeOD) δ 8.35 (t, J = 16.7 Hz, 2H), 7.71 (s, 2H), 7.38 (d, J = 8.8 Hz, 2H), 7.30 (t, J = 10.4 Hz, 2H), 6.28 (t, J = 21.9 Hz, 2H), 4.18 (s, 4H), 3.00 – 2.81 (m, 4H), 2.67 (d, J = 30.3 Hz, 4H), 1.93 (t, J = 22.9 Hz, 10H), 1.70 (s, 12H), 1.56 (d, J = 18.4 Hz, 18H). $^{13}$C NMR (101 MHz, MeOD) δ 171.68 (2×C), 153.70 (2×C), 148.62 (2×C), 143.02 (2×C), 142.03 (2×C), 137.52 (2×C), 137.13 (2×CH), 126.36 (C), 118.59 (2×CH), 112.67 (2×CH), 111.21 (2×CH), 100.90 (2×CH), 79.76 (2×C), 50.43 (2×$CH_2$), 49.31 (2×$CH_2$), 48.51 (2×$CH_2$), 43.80 (2×C), 27.38 (6×$CH_3$), 27.08 (4×$CH_3$), 25.99 (2×$CH_2$), 22.24 (2×$CH_2$), 20.79 ($CH_2$). TOF-MS calculated for $C_{48}H_{64}ClN_4O_{10}S_2^-$ [$M^-$]: 955.3758 (100%), found 955.3759.

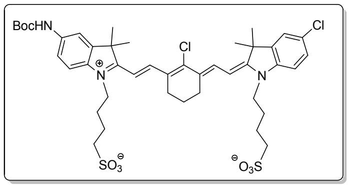


**Characterization of 5b.** (yield: 34.6%) $^{1}$H NMR (400 MHz, $CD_3OD$) δ 8.32 (ddd, J = 71.8, 47.2, 13.7 Hz, 3H), 7.46 – 7.21 (m, 4H), 6.55 – 5.99 (m, 3H), 4.36 – 4.00 (m, 4H), 2.89 (s, 4H), 2.70 (s, 4H), 1.93 (s, 10H), 1.68 (d, J = 15.0 Hz, 12H), 1.51 (s, 9H). $^{13}$C NMR (151 MHz, MeOD) δ 174.83 (C), 171.70 (C), 168.43 (C), 153.57 (C), 148.64 (C), 145.95 (C), 143.06 (CH), 142.28 (C), 141.67 (C), 140.17 (C), 136.42 (C), 128.69 (CH), 128.25 (CH), 127.85 (CH), 126.52 (C), 122.41 (C), 118.67 (CH), 112.75 (CH), 110.78 (CH), 104.42 (CH), 100.92 (CH), 98.41 (CH), 79.96 (C), 50.44 (2×$CH_2$), 49.32 (2×$CH_2$), 44.64 (2×$CH_2$), 43.18 (2×C), 27.39 (3×$CH_3$), 27.12 (4×$CH_3$), 26.01 (2×$CH_2$), 22.27 (2×$CH_2$), 20.77 ($CH_2$). TOF-MS calculated for $C_{43}H_{54}Cl_2N_3O_8S_2^-$ [$M^-$]: 874.2729 (100%), found 874.2730.

**Characterization of 5c.** (yield: 35.3%) $^{1}$H NMR (400 MHz, $CD_3OD$) δ 8.39 (d, J = 13.9 Hz, 1H), 8.34 – 8.23 (m, 1H), 7.71 (d, J = 21.5 Hz, 1H), 7.49 – 7.33 (m, 4H), 7.28 – 7.16 (m, 2H),

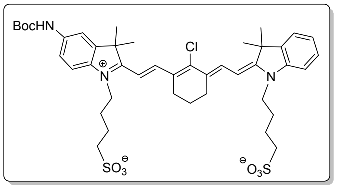


6.40 – 6.15 (m, 2H), 4.30 – 4.06 (m, 4H), 2.90 (d, J = 6.6 Hz, 4H), 2.72 (d, J = 5.4 Hz, 4H), 1.96 (d, J = 7.7 Hz, 10H), 1.69 (d, J = 3.7 Hz, 6H), 1.67 (d, J = 2.4 Hz, 6H), 1.53 (d, J = 17.3 Hz, 9H). $^{13}$C NMR (151 MHz, MeOD) δ 173.33 (C), 172.79 (C), 171.69 (C), 170.72 (C), 153.72 (C), 149.63 (C), 148.95 (CH), 143.99 (C), 142.52 (C), 142.21 (C), 141.23 (C), 138.37 (CH), 137.01 (CH), 128.61 (C), 126.93 (CH), 125.18 (CH), 122.32 (CH), 118.45 (CH), 110.92 (CH), 102.73 (CH), 101.15 (CH), 99.45 (CH), 79.79 (C), 50.46 (2×$CH_2$), 49.86 (2×$CH_2$), 49.27 (2×$CH_2$), 43.77 (2×C), 27.42 (3×$CH_3$), 27.08 (4×$CH_3$), 25.99 (2×$CH_2$), 22.27 (2×$CH_2$), 20.78 ($CH_2$). TOF-MS calculated for $C_{43}H_{55}ClN_3O_8S_2^-$ [$M^-$]: 840.3125 (100%), found 840.3125.

**Compound 6a–6c.** General synthetic procedure of compound **6a–6c**. Compound **5a–5c** (0.4 mmol), desiccant $K_2CO_3$ (118.8 mg, 0.9 mmol), tetrakis (triphenylphosphine) palladium (26.5 mg, 0.02 mmol) and 4-carboxyphenylboronic acid (119.4 mg, 0.7 mmol) were mixed in 2.0 mL distilled water and stirred at 95 °C for 3 h under argon. The crude product was purified by silica gel based column chromatography with an eluting solvent system in which the gradient of $CH_2Cl_2$/$CH_3OH$ changed from 90% to 70%. Removal of solvent and dehydration under vacuum afforded the purified product as green powder.

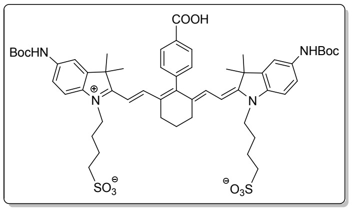


**Characterization of 6a.** (yield: 53.6%) $^1$H NMR (400 MHz, CD3OD) δ 8.32 (s, 2H), 7.42 (s, 2H), 7.21 (d, J = 7.3 Hz, 4H), 7.11 (d, J = 9.3 Hz, 4H), 6.13 (d, J = 13.0 Hz, 2H), 4.05 (s, 4H), 2.90 (s, 4H), 2.68 (s, 4H), 2.02 (s, 2H), 1.91 (s, 8H), 1.51 (d, J = 20.7 Hz, 18H), 1.19 (d, J = 78.2 Hz, 12H). $^{13}$C NMR (151 MHz, MeOD) δ 170.85 (2×C), 160.61 (C), 153.65 (2×C), 146.85 (2×C), 142.13 (2×C), 141.35 (2×C), 137.15 (C), 136.95 (2×CH), 132.89 (C), 130.79 (2×CH), 129.67 (2×CH), 129.06 (C), 127.99 (2×C), 118.42 (2×CH), 112.49 (2×CH), 110.64 (2×CH), 99.48 (2×CH), 79.67 (2×C), 50.48 (2×$CH_2$), 48.50 (2×$CH_2$), 43.56 (2×C), 27.37 (6×$CH_3$), 26.77 (4×$CH_3$), 25.92 (2×$CH_2$), 24.30 (2×$CH_2$), 22.20 (2×$CH_2$), 21.22 ($CH_2$). TOF-MS calculated for $C_{55}H_{69}N_4O_{12}S_2^-$ [$M^-$]: 1041.4359 (100%), found 1041.4349.

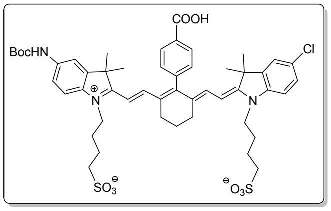


**Characterization of 6b.** (yield: 42.3%) $^1$H NMR (400 MHz, CD3OD) δ 8.23 (d, J = 6.8 Hz, 2H), 7.39 – 7.21 (m, 9H), 7.11 (dd, J = 28.1, 11.2 Hz, 2H), 6.24 (d, J = 13.9 Hz, 1H), 4.03 (d, J = 49.3 Hz, 4H), 2.87 (s, 4H), 2.73 (s, 4H), 1.90 (s, 10H), 1.65 – 1.33 (m, 9H), 1.15 (s, 12H). $^{13}$C NMR (151 MHz, MeOD) δ 173.92 (C), 171.63 (C), 16 7.99 (C), 161.37 (C), 160.00 (C), 153.61 (C), 149.42 (C), 147.85 (C), 144.03 (CH), 142.63 (C), 141.96 (C), 141.02 (C), 132.61 (C), 131.99 (C), 131.65 (2×CH), 131.28 (2×CH), 130.05 (C), 129.70 (CH), 129.31 (CH), 128.41 (CH), 128.05 (C), 122.56 (CH), 111.77 (CH), 110.46 (CH), 102.38 (CH), 100.31 (CH), 97.93 (CH), 79.89 (C), 50.37 ($CH_2$), 49.46 (2×$CH_2$), 48.63 (C), 44.18 ($CH_2$), 43.59 (C), 42.95

($CH_2$), 27.31 (2×$CH_3$), 26.66 ($CH_2$), 26.45 (3×$CH_3$), 26.15 ($CH_2$), 25.70 ($CH_2$), 24.27 (2×$CH_3$), 22.16 ($CH_2$), 21.08 (2×$CH_2$). TOF-MS calculated for $C_{50}H_{59}ClN_3O_{10}S_2^-$ [$M^-$]: 960.3336 (100%), found 960.3334.

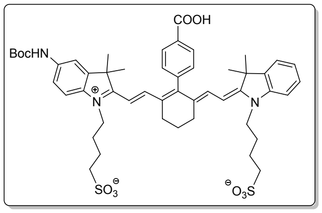


**Characterization of 6c.** (yield: 49.1%) $^1H$ NMR (400 MHz, CD3OD) δ 8.20 (s, 2H), δ 7.40 (d, J = 17.7 Hz, 1H) , 7.33 – 7.21 (m, 6H), 7.11 (dd, J = 20.4, 12.7 Hz, 4H), 6.31 – 6.13 (m, 2H), 4.05 (d, J = 31.0 Hz, 4H), 2.88 (s, 4H), 2.72 (s, 4H), 2.02 (s, 2H), 1.91 (s, 8H), 1.42 (d, J = 50.4 Hz, 9H), 1.09 (d, J = 9.0 Hz, 12H). $^{13}C$ NMR (151 MHz, MeOD) δ 172.77 (C), 171.90 (C), 170.11 (C), 167.93 (C), 160.76 (C), 153.64 (C), 147.52 (C), 145.84 (CH), 144.11 (C), 142.14 (C), 140.73 (C), 137.73 (C), 136.81 (C), 131.09 (C), 129.66 (C), 128.42 (2×CH), 124.71 (2×CH), 123.90 (CH), 122.04 (CH), 118.56 (CH), 112.62 (CH), 111.43 (CH), 110.59 (CH), 110.07 (CH), 101.02 (CH), 99.92 (CH), 98.69 (CH), 79.84 (C), 50.54 (2×$CH_2$), 48.52 (2×$CH_2$), 43.45 (2×C), 27.39 ($CH_2$), 26.60 (4×$CH_3$), 25.88 (2×$CH_2$), 24.29 (3×$CH_3$), 22.22 (2×$CH_2$), 21.18 (2×$CH_2$). TOF-MS calculated for $C_{50}H_{60}N_3O_{10}S_2^{2-}$ [$M^-$]: 926.3726 (100%), found 926.3731.

**Compound IR1–3.** General synthetic procedure of compound **IR1–3**. Deprotection of compound **6a**–**6c** (0.1 mmol) in a mixture of TFA and $CH_2Cl_2$ (V/V = 1:1) offered compound **IR1–3**. The crude product was purified by silica gel based column chromatography with an eluting solvent system in which the gradient of $CH_2Cl_2/CH_3OH$ (containing 0.1% trimethylamine) changed from 90% to 60%. Removal of solvent and dehydration under vacuum afforded the purified product as green powder.

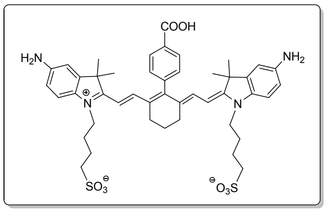


**Characterization of IR1.** (yield: 77.4%) $^1H$ NMR (400 MHz, DMSO) δ 8.09 (d, J = 7.3 Hz, 2H), 7.10 (d, J = 7.4 Hz, 2H), 6.95 (dd, J = 19.7, 11.1 Hz, 4H), 6.54 (s, 2H), 6.48 (s, 2H), 6.00 (d, J = 13.7 Hz, 2H), 5.21 (s, 4H), 3.95 (s, 4H), 2.59 (s, 4H), 2.48 (s, 4H), 1.89 (s, 2H), 1.66 (s, 8H), 1.00 (s, 12H). $^{13}C$ NMR (151 MHz, DMSO) δ 170.36 (2×C), 168.51 (C), 159.11 (2×C), 147.40 (2×C), 144.51 (2×C), 142.57 (2×C), 140.10 (C), 139.64 (2×CH), 132.61 (C), 129.60 (2×CH), 129.24 (2×CH), 128.66 (C), 113.57 (2×CH), 112.11 (2×CH), 108.43 (2×CH), 99.33 (2×CH), 51.26 (2×$CH_2$), 48.39 (2×$CH_2$), 43.84(2×C), 27.71 (4×$CH_3$), 26.62 ($CH_2$), 24.66 (2×$CH_2$), 23.01 (2×$CH_2$), 21.60 (2×$CH_2$). TOF-MS calculated for $C_{45}H_{53}N_4O_8S_2^-$ [$M^-$]: 841.3310 (100%), found 841.3315.

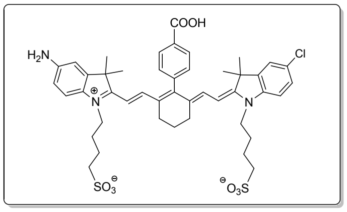


**Characterization of IR2.** (yield: 69.5%) $^1H$ NMR (400 MHz, $CD_3OD$) δ 8.16 (t, J = 13.2 Hz, 2H), 7.32 – 7.04 (m, 6H), 6.90 (d, J = 8.4 Hz, 1H), 6.76 – 6.58 (m, 3H), 6.44 (d, J = 13.5 Hz, 1H), 5.85 (d, J = 11.5 Hz, 1H), 4.22 (s, 2H), 3.81 (s, 2H), 2.88 (d, J = 6.1 Hz, 4H), 2.67 (s, 4H), 1.93 (s, 10H), 1.35 – 0.78 (m,

12H). $^{13}$C NMR (151 MHz, MeOD) δ 174.78 (C), 170.00 (C), 166.27 (C), 159.24 (C), 150.83 (C), 150.40 (C), 145.96 (C), 143.80 (C), 143.17 (CH), 141.86 (C), 133.55 (C), 133.04 (C), 132.53 (2×CH), 131.48 (C), 131.27 (2×CH), 130.13 (C), 129.66 (CH), 128.84 (CH), 124.24 (CH), 123.93 (CH), 116.06 (C), 115.41 (CH), 111.20 (CH), 109.89 (CH), 106.12 (CH), 98.02 (CH), 52.45 ($CH_2$), 52.10 (2×$CH_2$), 51.47 (C), 50.34 ($CH_2$), 48.67 (C), 46.48 ($CH_2$), 44.34 (2×$CH_3$), 28.65 ($CH_2$), 28.11 ($CH_2$), 27.12 ($CH_2$), 26.09 (2×$CH_3$), 24.07 ($CH_2$), 23.05 (2×$CH_2$). TOF-MS calculated for $C_{45}H_{51}ClN_3O_8S_2^-$ [$M^-$]: 860.2812 (100%), found 860.2806.

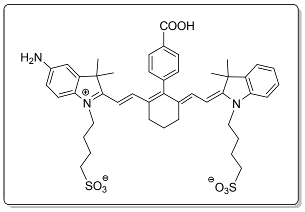


**Characterization of IR3.** (yield:66.9%) $^{1}$H NMR (400 MHz, DMSO) δ 8.14 (d, J = 6.7 Hz, 2H), 7.44 – 7.29 (m, 4H), 7.19 (d, J = 6.8 Hz, 1H), 7.09 (d, J = 15.0 Hz, 2H), 6.93 (s, 1H), 6.74 – 6.55 (m, 3H), 6.46 (d, J = 15.0 Hz, 1H), 5.88 (d, J = 12.9 Hz, 1H), 4.24 (s, 2H), 3.87 (s, 2H), 2.65 (s, 4H), 2.50 (s, 4H), 1.88 (d, J = 38.9 Hz, 2H), 1.73 (d, J = 38.3 Hz, 8H), 0.97 (d, J = 64.3 Hz, 12H). $^{13}$C NMR (151 MHz, DMSO) δ 172.01 (C), 167.77 (C), 165.24 (C), 157.10 (C), 149.70 (2×C), 147.47 (C), 144.39 (CH), 143.99 (C), 143.48 (C), 140.50 (2×C), 139.86 (2×CH), 131.47 (C), 131.37 (2×CH), 130.76 (C), 130.03 (CH), 129.85 (CH), 128.53 (CH), 122.56 (CH), 114.57 (CH), 113.87 (CH), 109.35 (CH), 107.91 (CH), 105.07 (CH), 96.61 (CH), 51.41 ($CH_2$), 51.04 (2×$CH_2$), 49.62 (C), 49.06 ($CH_2$), 47.04 (C), 45.05 ($CH_2$), 42.79 (2×$CH_3$), 27.82 ($CH_2$), 27.21 ($CH_2$), 26.03 ($CH_2$), 24.59 (2×$CH_3$), 22.86 ($CH_2$), 21.48 (2×$CH_2$). TOF-MS calculated for $C_{45}H_{52}N_3O_8S_2^-$ [$M^-$]: 826.3201 (100%), found 826.3195.

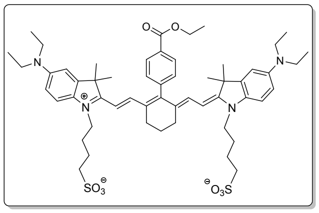


**Compound IR4.** A mixture of compound **IR1** (43.2 mg, 0.05 mmol), bromoethane (5.45 g, 50 mmol) and N,N-Diisopropylethylamine (64.7 mg, 0.5 mmol) dissolved in DMF was stirred at room temperature for 24h under argon. The mixture was dropped into the ice-cold diethyl ether and a brown precipitate was filtrated. The crude product was purified by column chromatography with an eluting solvent system in which the gradient of $CH_2Cl_2/CH_3OH$ changed from 90% to 75%. Removal of solvent afforded pure **IR4** as a brown-yellow powder (20.4 mg，41.6 %). $^{1}$H NMR and $^{13}$C NMR spectrum were not resolved due to the strong H-aggregates in solution.[1] TOF-MS calculated for $C_{55}H_{73}N_4O_8S_2^-$ [$M^-$]: 981.4875 (100%), found 981.4869.

**Chemical purity of IR1–4.** HPLC experiments were conducted to test the chemical purity of **IR1–4**. A reversed-phase C18 analytical column was used as the stationary phase and a mixture of 5.0 mM tetrabutyl ammonium bromide aqueous solution (eluent A) and methanol (eluent B) with a linear gradient elution profile (0 min, 20% B; 4 min, 95% B; 6 min, 95% B; 10 min, 20% B; 18min, 20% B) was used as the mobile phase. The signals were detected at a wavelength of 750 nm in the diode array detection (DAD) of Agilent 1100 series. The temperature of the column was maintained at 25 °C. 10 μL sample solution (20 μM) was injected and the flow rate of the mobile phase was 0.5 mL/min. All probes showed >95%

chemical purity according to the normalization method of peak area. The HPLC spectra of **IR1–4** were listed in the supplemental spectra part.

## 4. Photospectroscopic Studies

**Preparation of the stock solution.** Stock solutions of **IR1–4** were prepared in dimethyl sulfoxide (DMSO) with a concentration of 10 mM. And then, the solutions were sub-packaged and stored at – 40 °C.

**Absorption spectra.** Working solutions (1.0 μM) were prepared by diluting the stock solution in disodium hydrogen phosphate-citric acid buffer solution with pH ranging from 7.4 to 2.4 (pH changed with an interval of 0.2 units). All absorption spectra were recorded in a quartz cuvette (10 × 10 mm) at room temperature (r.t.). The scanning wavelength range is 400–900 nm and scanning speed is 1.0 nm/s (slit width: 5 nm).

**Emission spectra.** Working solutions (1.0 μM) were prepared under the same procedures as absorption test. All emission spectra were performed with a photomultiplier tube at r.t. Fluorescence emission spectra of all samples were collected at a 90-degree angle relative to the excitation light path. All fluorophores were excited at 750 nm and recorded from 760 nm to 900 nm.

**pH dependent temperature profile.** Disodium hydrogen phosphate-citric acid buffer solution with different pH values (2.4, 3.0, 3.5, 4.0, 4.5, 5.0, 5.5, 6.0, 6.5, 7.0, 7.4) were prepared. A final concentration of 10 μM **IR1–4** in buffered solutions (100 μL) were added into centrifuge tubes. The tubes were irradiated under 750 nm laser at 6.0 $W/cm^2$ for 180 s. The temperature curves were obtained with an infrared thermal imaging camera.

**Laser power dependent temperature profile.** Disodium hydrogen phosphate-citric acid buffer solution with pH 7.4 and 4.0 were prepared. A final concentration of 10 μM **IR1–4** were added into centrifuge tubes in the above buffered solutions (100 μL) that mimic neutral and acidic environment. The tubes were irradiated under 750 nm laser at selected laser powers (1.2, 2.4, 3.6, 4.8 and 6.0 $W/cm^2$) for 180 s respectively. The temperature curves were obtained with an infrared thermal imaging camera.

**Concentration dependent temperature profile.** Disodium hydrogen phosphate-citric acid buffer solution with pH 7.4 and 4.0 were prepared. **IR1–4** were dissolved in the two represented buffer solutions with different concentrations (0, 1, 5, 10, 20 μM). Then the solutions were added into centrifuge tubes respectively and irradiated under 750 nm laser at 6.0 $W/cm^2$ for 180 s. The temperature curves were obtained with an infrared thermal imaging camera.

**Quantum yield measurements.** The fluorescence quantum yields of **IR1–4** in disodium hydrogen phosphate-citric acid buffer solution at pH 7.4 and pH 2.4 were determined with reference of that of Indocyanine Green (ICG) in DMSO solution (Q.Y. = 0.12). The

absorption spectra of each compound were measured with a concentration of 1.0 μM and the emission spectra with same concentration were excited at 750 nm wavelength. The fluorescence of each fluorescence spectrum were integrated, and the quantum yields were determined according to Equation 1:

$$\Phi_S=\Phi_R\times\frac{I_S}{I_R}\times\frac{A_R}{A_S}\times\frac{\eta_s^2}{\eta_R^2} \qquad \text{Equation 1}$$

In Equation 1, subscripts **s** and **r** refer to sample and reference respectively; **Φ** represents the fluorescence quantum yield; **I** stands for the measured integrated emission intensity; **η** is the refractive index; **A** is the optical density.

**NIR fluorescence images.** A final concentration of 1.0 μM **IR1**–**4** in buffered solutions (100 μL) with pH increasing from 2.4 to 7.4 (2.4, 3.0, 3.5, 4.0, 4.5, 5.0, 5.5, 6.0, 6.5, 7.0, 7.4) were added to centrifuge tubes. The NIR fluorescence images were collected by an IVIS Spectrum equipped with a 745/800 filter set.

**Measurements of $pKa_{fluo}$.** pH dependent NIR fluorescent intensities of **IR1**–**4** at their maximal emission wavelength were obtained from the emission spectra. All the values are normalized to the values under pH 2.4 respectively. The normalized fluorescent intensities were plotted against pHs in GraphPad Prism software. The plots were fitted into nonlinear regression curves (log(agonist) vs. response – Variable slope) and the pKa values were determined as the $logEC_{50}$ values.

**Thermalgraphic images.** A final concentration of 10 μM **IR1**–**4** in buffered solutions (100 μL) with same pH range as above were added to centrifuge tubes. The solutions were irradiated under 750 nm laser at a power density of 6.0 $W/cm^2$ for 180s. The thermographic maps were captured when the solutions reached their maximal temperature values by an infrared thermal imaging camera.

**Measurements of $pKa_{\Delta T}$.** pH dependent temperature increment ($\Delta T = T - T_0$) were determined as the maximal values (T) minus the starting temperature ($T_0$) at each pH point according to the pH dependent temperature profiles. All the temperature increment values were normalized to the values under pH 7.4 respectively. The data analysis were similar as the procedures of $pKa_{\Delta T}$ measurement.

**ROS levels detection in solutions.** A final concentration of 10 μM **IR2** and 5 μM DCFH-DA in buffered solutions (100 μL) with same pH range as above were added to a 96-well plate. The solutions were irradiated under a 750 nm laser at a power density of 6.0 $W/cm^2$ for 10 min or not. The fluorescence images were collected by an IVIS Spectrum equipped with a 500/540 filter set.

**Photothermal stability evaluation.** Working solutions (10 μM) of **IR1**–**4** were prepared in disodium hydrogen phosphate-citric acid buffer solutions (pH 4.0 and pH 7.4) respectively. All fluorophores were excited at 750 nm (6.0 W/cm$^2$, 10 min) and the temperature curves were obtained with an infrared thermal imaging camera.

**Fluorescent stability evaluation.** Working solutions (1.0 μM) of **IR1**–**4** were prepared in pH 4.0 disodium hydrogen phosphate-citric acid buffer solution. All fluorophores were excited at 750 nm and recorded at maximal emission wavelengths (755 nm, 761 nm, 756 nm, and 760 nm for **IR1–4** respectively). Fluorescence intensities were measured every 5 min for 1 h at r.t.

**Reversibility evaluation.** Working solutions (1.0 μM) of **IR1**–**4** were prepared in disodium hydrogen phosphate-citric acid buffer solution. pH values of working solutions were reversibly adjusted between 7.4 and 4.0 by adding hydrochloric acid (5.0 M) and sodium hydroxide (5.0 M) solutions. All fluorophores were excited at 750 nm and recorded at maximal emission wavelengths (same as mentioned above). Fluorescence signals were measured for five rounds.

**Selectivity evaluation.** A final concentration of 200 mM $K^+$, $Ca^{2+}$, $Mg^{2+}$, $Zn^{2+}$, $Co^{2+}$, $Fe^{3+}$, $Fe^{2+}$, $NH_4^+$, cysteine, proline, glycine, histidine and $H_2O_2$ were prepared in disodium hydrogen phosphate-citric acid buffer solution with pH value determined as 4.0 and 7.4 respectively. Stock solutions of **IR1–4** were diluted to 1.0 μM in the above solutions. Fluorescent intensities were measured at their maximal emission wavelengths (same as mentioned above) upon excitation at 750 nm.

## 5. *In Vitro* Microscopic and Flow Cytometric Studies

**Cell culture.** Human adenocarcinoma cell line HeLa, human hepatocellular liver carcinoma cell line HepG2, human normal liver cell line HL-7702, human embryonic kidney cell line HEK-293, and human umbilical vein endothelial cell line HUVEC were used for *in vitro* studies. Cells were grown in DMEM (for HeLa, HepG2, HUVEC, and HEK-293) or RMPI 1640 (for HL-7702) culture medium containing 10% fetal bovine serum (FBS) and 1% penicillin and streptomycin (PS) at 37 °C in a humidified incubator of 5% $CO_2$. Cells were carefully harvested and split when they reached 80% confluence to maintain exponential growth.

**Cytotoxicity studies.** The CCK-8 cell proliferation assay was applied to determine the viabilities of the cells treated with **IR2**. A cell monolayer in exponential growth was harvested using 0.25% trypsin, and a single-cell suspension was obtained. Cell suspensions with a density of $1.0 \times 10^4$ cells/well in 100 μL cell culture medium were added to 96-well plates. After cell attachment, the cells were treated by **IR2** with different concentrations (0.01–200 μM) that was sterile filtered through MILLEX®-HV 0.22 μm syringe filter in DMEM/RMPI 1640. After another 24 h incubation, cells were washed twice with phosphate buffer saline (PBS) and incubated with fresh medium containing 10 μL CCK-8. The cell viabilities were measured in an iMark Microplate Absorbance Reader according to the protocol. Cell viability (%) = mean of absorbance value of treatment group / mean of absorbance value of control × 100%.

**Time dependent cellular uptake.** HepG2 cells ($2 \times 10^5$ cells/well) were seeded into 35 mm glass bottom cell culture dishes (φ20 mm, NEST Biotechnology) in 2.0 mL culture medium. After cell attachment, the culture medium was replaced by the DMEM containing **IR2** (20 μM) and incubated for another 0.5, 4, 12, and 24 h respectively. The cells were washed twice with PBS ad incubated with PBS (pH 4.0) for another 10 min before cellular imaging. The fluorescent images were obtained by Zeiss LSM 880 confocal laser scanning microscope using a 20X objective lenses. The red fluorescence of **IR2** were obtained by using a 633 nm laser. Filter set: Long pass (LP) 645 nm.

***In vitro* cellular uptake.** HepG2 cells ($2 \times 10^5$ cells/well) were seeded into 35 mm glass bottom cell culture dishes (φ20 mm, NEST Biotechnology) in 2.0 mL culture medium. The culture medium was replaced by the DMEM containing **IR2** (20 μM) after cell attachment and incubated for another 12 h. LysoTracker Green DND-26 (500 nM for 30 min) and MitoTracker Green FM (100 nM for 30 min) were added respectively before cellular imaging. The cells were washed twice with PBS and observed by Zeiss LSM 880 confocal laser scanning microscope using a 63X oil objective lenses. A portion of the cells pre-treated with **IR2** and MitoTracker were incubated with PBS (pH 4.0) for another 10 min before cellular imaging. The green fluorescence of LysoTracker Green DND-26 and MitoTracker

Green FM were collected by using a 488 nm laser. Filter set: Band pass (BP) 495–550 nm. The NIR fluorescence of **IR2** were obtained by using a 633 nm laser. Filter set: Long pass (LP) 645 nm. Similar procedures were taken for HeLa, HL-7702, HUVEC and HEK-293 cell imaging.

**Lysosomal activation of IR2.** HepG2 cells (2 × $10^5$ cells/well) were seeded on 35 mm glass bottom cell culture dishes and cultured for 24 h in 2.0 mL medium. The cells were incubated in DMEM containing **IR2** (20 μM) for 12 h and meanwhile spiked with or without BFA (100 nM) for 2 h, and then Lysotracker green DND-26 (500 nM) for 30 min. A portion of the cells pre-treated with BFA, **IR2** and LysoTracker were incubated with PBS (pH 4.0) for another 10 min before cell imaging. The cells were imaged by a Zeiss LSM 880 confocal fluorescence microscope with filter sets the same as the cellular uptake imaging above.

**Morphology change studies.** Morphology changes of different cells lines after **IR2**/laser treatment were studied under a Leica DMI4000D inverted microscope. HepG2 cells (2 × $10^5$ cells/well) were seeded into 35 mm cell culture dishes in 2.0 mL medium. 24 h after the cell attachment, the medium was replaced by the DMEM containing 20 μM **IR2**. The cells were incubated for another 12 h and washed twice with PBS prior to the application of laser. The dishes were placed on the Labnet AccublockTM digital dry bath incubator so that the cells were remained at 37 °C. The cells were irradiated with a 750 nm laser (6.0 W/$cm^2$) for 10 min. Bright filed pictures of the cells prior to laser, 30 min and 24 h after laser treatment were collected respectively. Similar procedures were taken for HeLa and HL-7702 cell imaging.

**Calcein-AM/PI staining.** The live/dead cells after laser treatment were visualized by calcein-AM/PI double staining kit. HepG2 cells (2 × $10^5$ cells/well) were seeded into 35 mm glass bottom cell culture dishes in 2.0 mL culture medium. 24 h after the cell attachment, the culture medium was replaced by the DMEM containing 20 μM **IR2** or DMEM only as control. As methods mentioned previously, both the groups were irradiated with a 750 nm laser (6.0 W/$cm^2$) for 10 min. After another 24 h incubation, cells were washed twice by PBS and stained with calcein-AM and propidium iodide (PI) for visualization of live/dead cells according to the protocol. The fluorescence images were obtained by Zeiss LSM 710 confocal microscope with proper filter sets (Calcein-AM: Ex: 488 nm; Em: 493–552 nm. PI: Ex: 561 nm Em: 566–697 nm). Similar procedures were taken for HeLa and HL-7702 cell imaging.

**Quantification of hyperthermia induced cell death.** Quantitative evaluation of photothermal efficacy was determined by CCK-8 assay. HepG2, HeLa, HL-7702, HUVEC, and HEK-293 cells (1 × $10^4$ cells/well) were seeded into 96-well plate and incubated with 100 μL media alone or containing 20 μM **IR2** for 12 h after cell adherence. The two groups of cells were then exposed to 6.0 W/$cm^2$ 750 nm laser irradiation for 10 min or not respectively.

After incubation for another 24 h and the cell viabilities of the four groups were quantified by the CCK-8 in an iMark Microplate Absorbance Reader.

**Time lapse imaging.** HepG2 cells were plated in 35 mm glass-bottomed dishes with 2 mL culture medium. The cells were then treated with 20 μM **IR2** for 12 h and 100 nM MitoTracker for 30 min prior to imaging. The bright filed images of cells and green fluorescent images of mitochondrial marker were obtained every 2 min for 12 min. And the cells were undergone photo-bleaching by a 633 laser (5.0 mW) for the rest of time during the whole acquisition process. The fluorescence images were taken with filter sets (Ex: 488 nm, Em: 495–550). Similar procedures were taken for HeLa and HL-7702 cell imaging.

**Detecting ROS levels within cells.** HepG2 cells ($1 \times 10^4$ cells/well) were seeded into a 96-well plate and incubated with 20 μM **IR2** for 12 h and 10 μM DCFH-DA for 30 min. The cells were then exposed to laser irradiation (6.0 W/cm$^2$, 750 nm) for 10 min prior imaging. As a positive control, the cells were treated with a Rosup (1 μL/mL) and DCFH-DA (10 μM) for 30 min prior imaging. Bright filed and fluorescent pictures of the cells were collected under a Leica DMI4000D inverted microscope. Filter sets (Ex: 480±20 nm, Em: 527±15 nm).

**JC-1 flow cytometric analysis.** Mitochondrial membrane potential was determined using JC-1 mitochondrial membrane potential assay kit. Briefly, after treatment with 20 μM **IR2** for 12 h followed by laser irradiation (6 W/cm$^2$, 10 min), the cells were collected and stained with 10 μg/mL JC-1 solution at 37 °C, 5% $CO_2$ for 15 min. The cells were washed with PBS twice and resuspended in 500 μL PBS. Cells were analyzed on BD FACSAria II flow cytometer using a 488-nm excitation light source and a band-pass filters 515–545 nm (green fluorescence) and a band-pass filter 564–606 nm (red fluorescence). The data were analyzed by FlowJo software. The report generated by plotting and gating for cell samples excluding debris. The cell population percentages of each quadrant were displayed.

**Annexin V-FITC/PI flow cytometric analysis.** The cell death pathway upon the **IR2**/laser irradiation was studied by flow cytometry after staining with annexin V-FITC/PI kit. After the same treatment of **IR2**/laser as described above, the cells were collected and washed with PBS twice. The cells were resuspended in 500 μL PBS and stained with Annexin V-FITC and PI solution at r.t. for 15 min. Cells were analyzed on BD FACSAria II flow cytometer using 488 nm excitation, a 515–545 nm band pass filter for FITC detection and a filter 665–685 nm for PI detection. The data report were obtained under similar process as JC-1 analysis above.

## 6. Supplemental Spectra

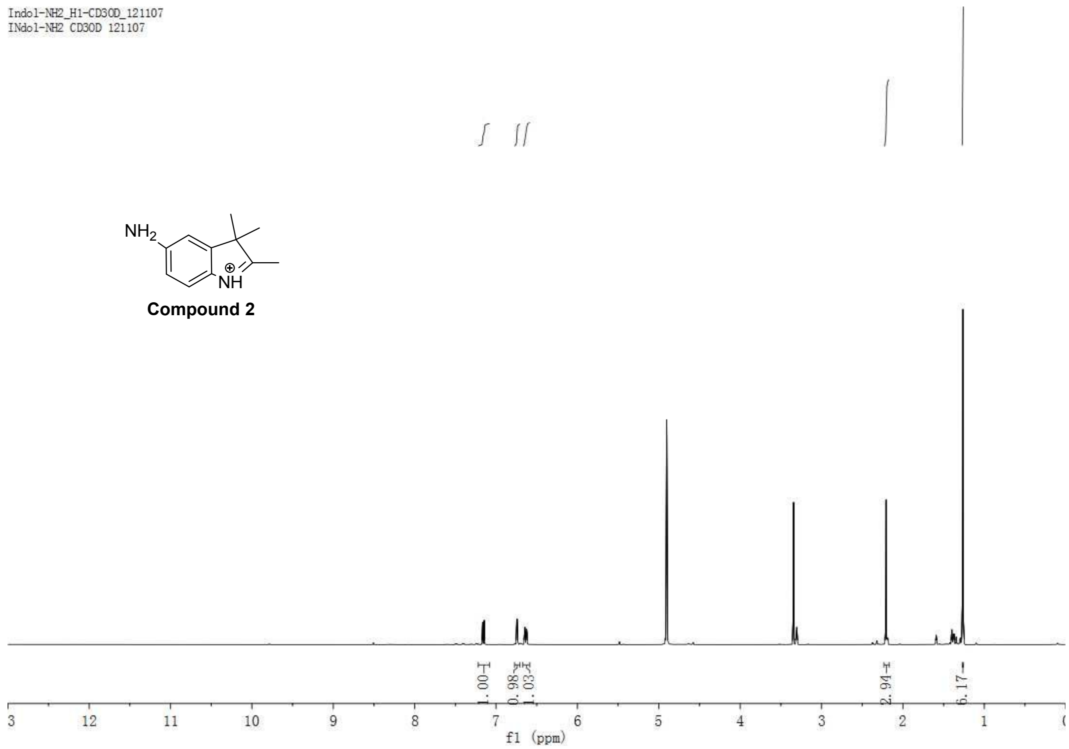


$^{1}H$ NMR Spectrum of **Compound 2** in $CD_3OD$

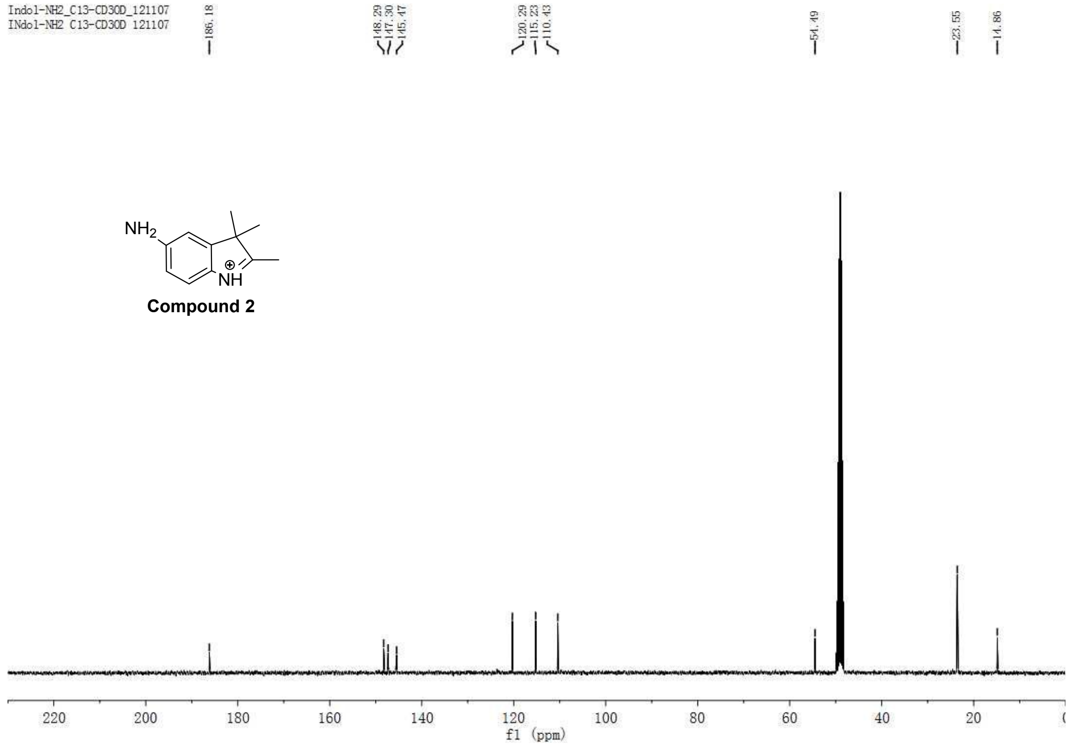


$^{13}C$ NMR Spectrum of **Compound 2** in $CD_3OD$

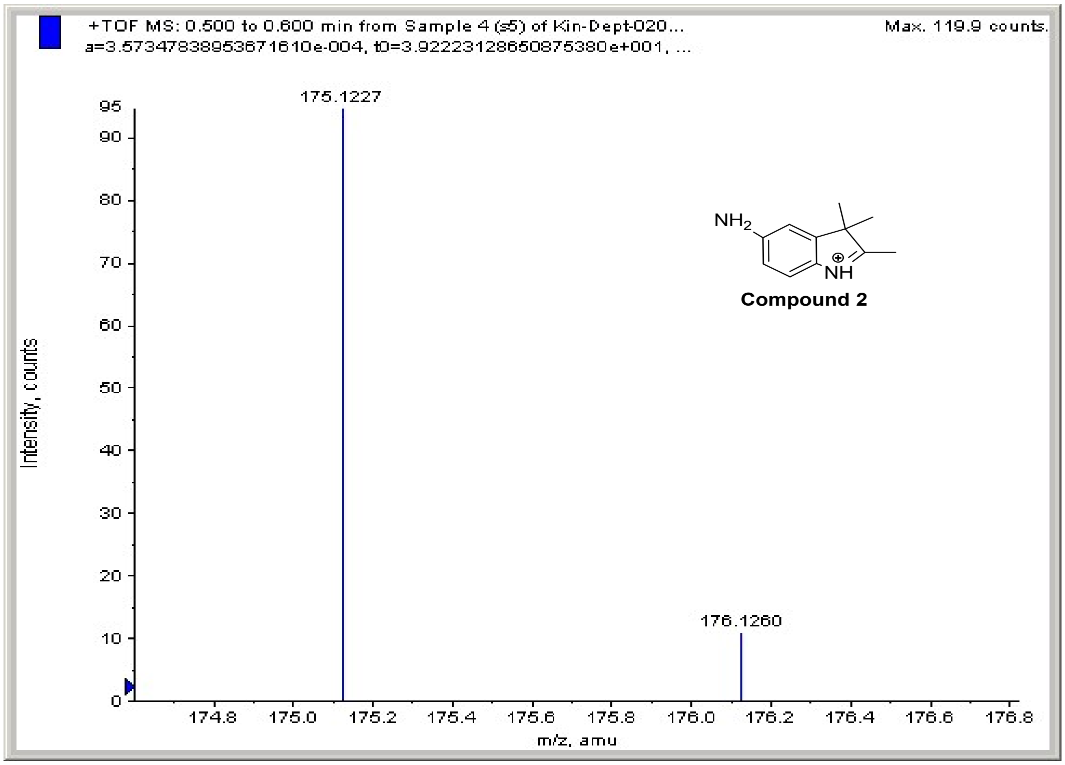


High resolution mass spectrum (ESI-MS) of **Compound 2**

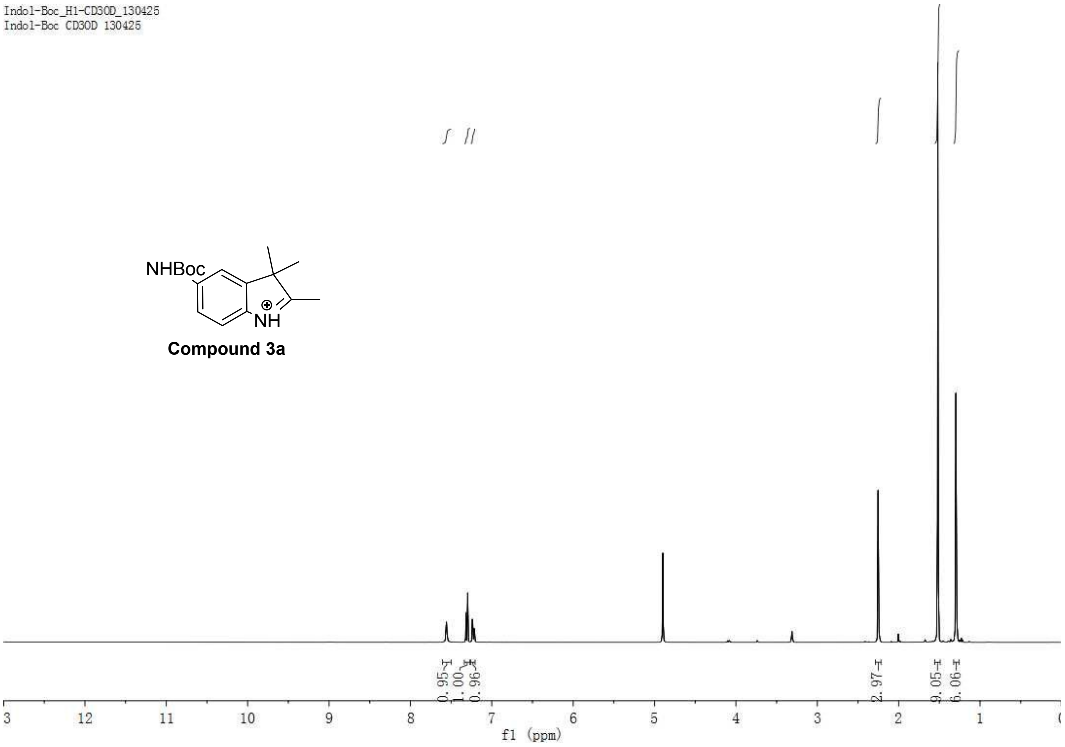


$^{1}H$ NMR Spectrum of **Compound 3a** in $CD_3OD$

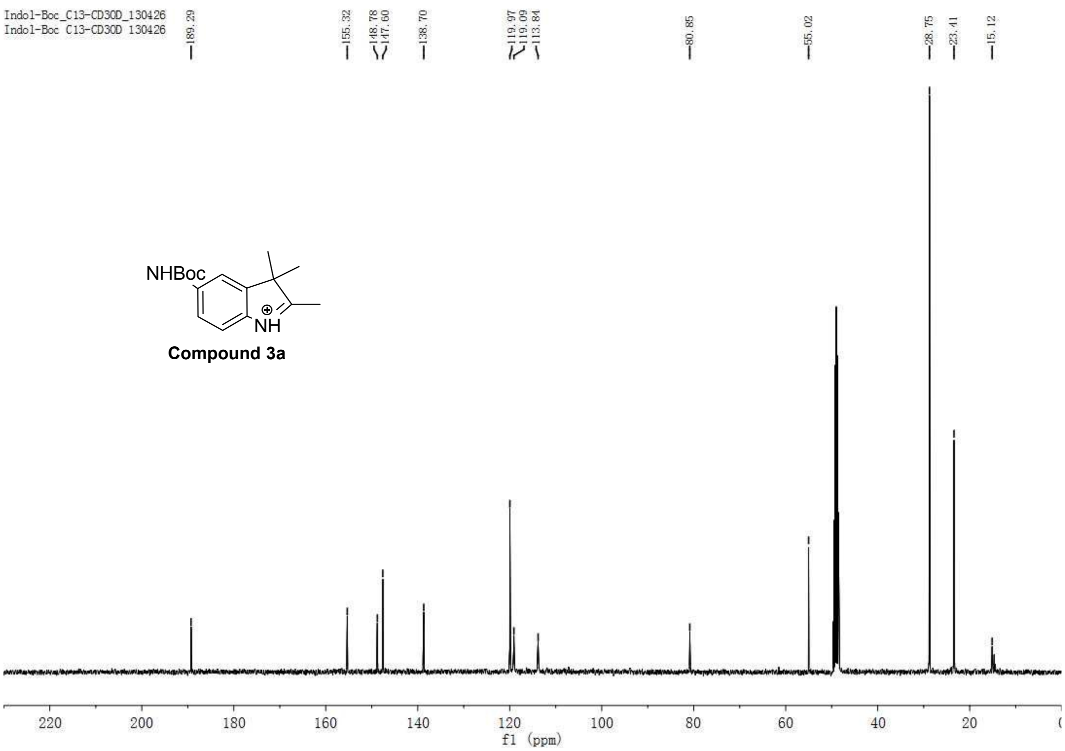


$^{13}C$ NMR Spectrum of **Compound 3a** in $CD_3OD$

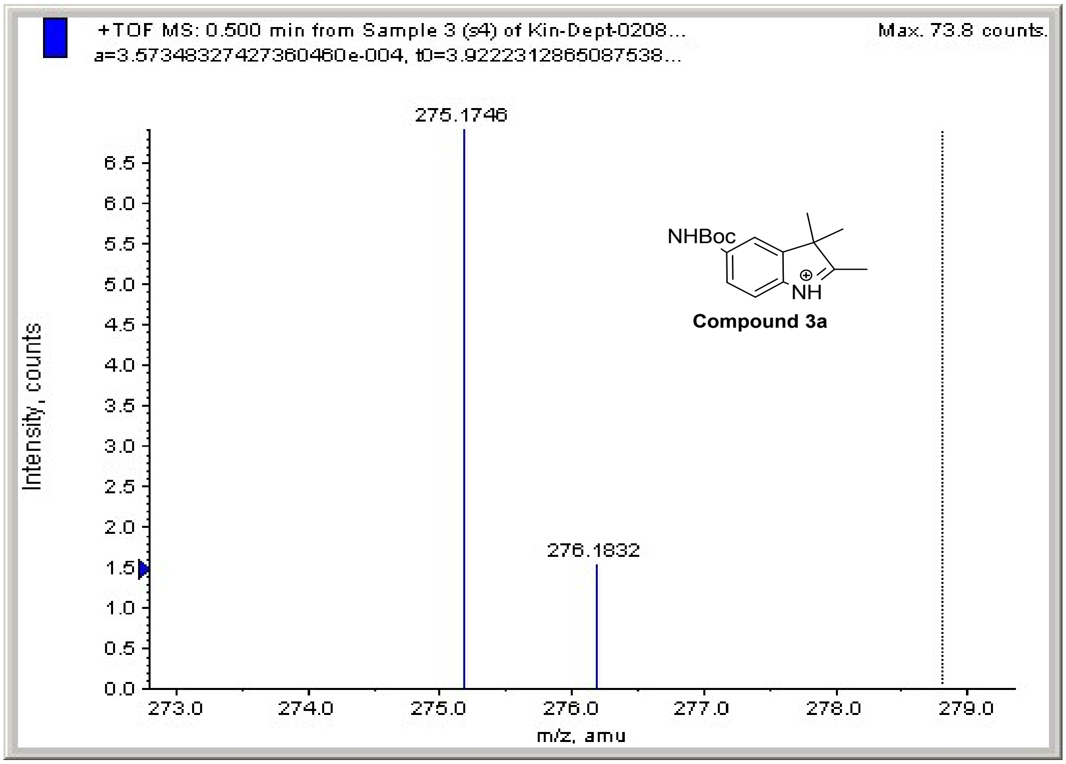


High resolution mass spectrum (ESI-MS) of **Compound 3a**

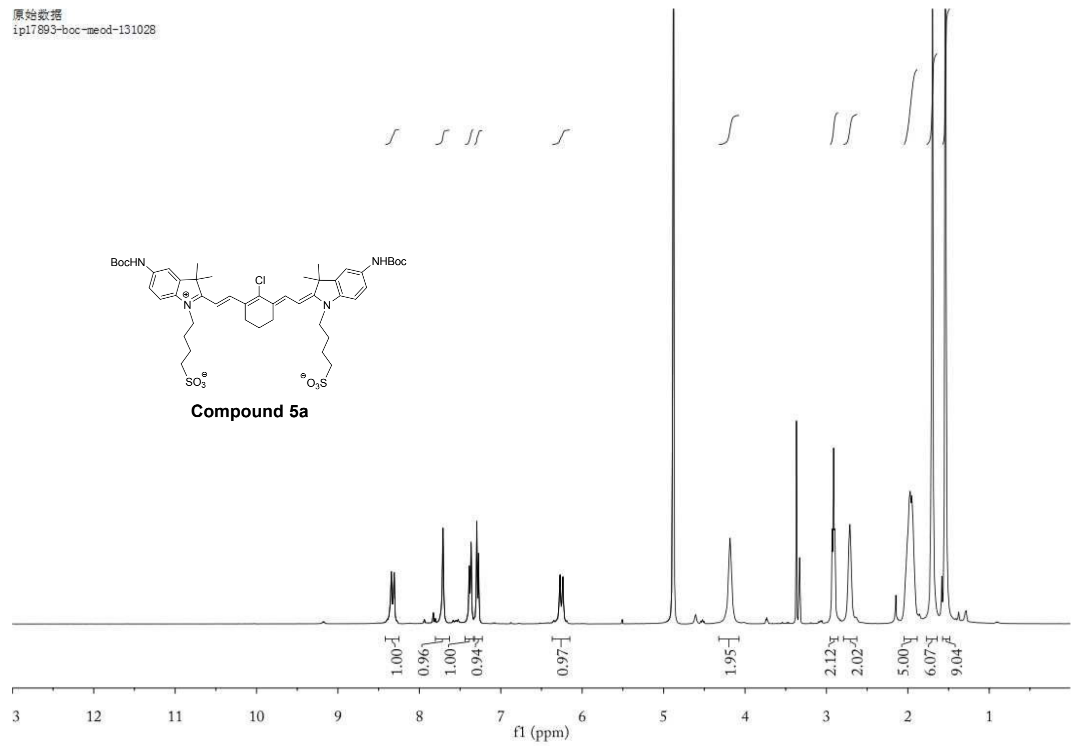


$^{1}H$ NMR Spectrum of **Compound 5a** in $CD_3OD$

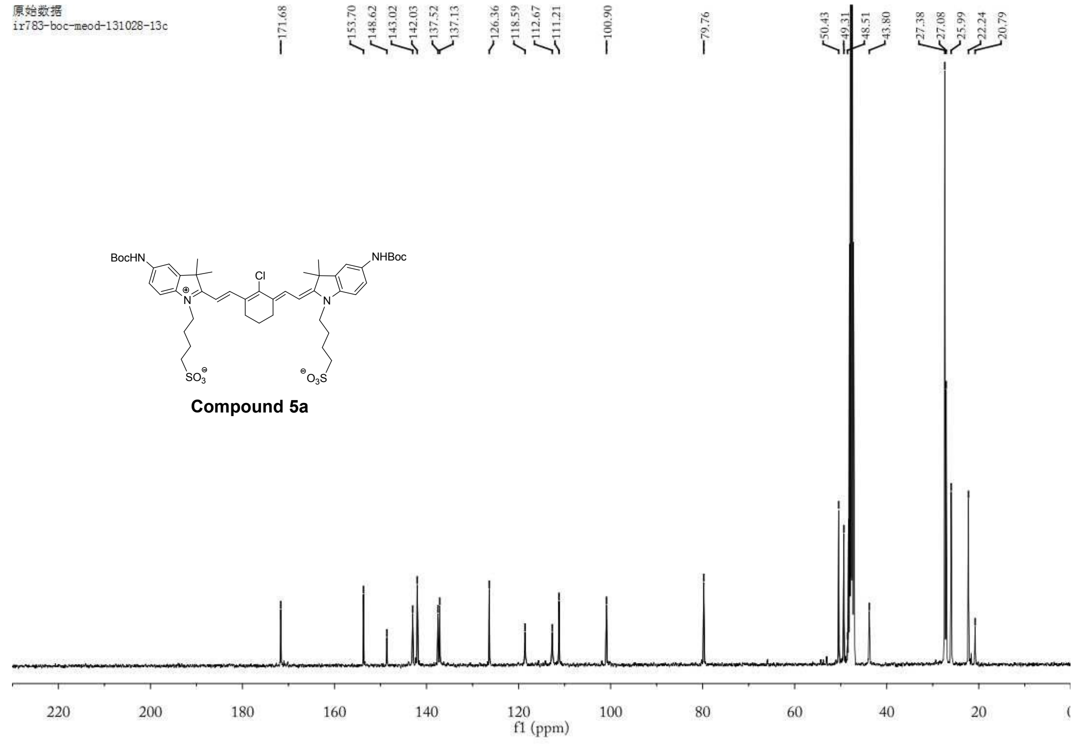


$^{13}C$ NMR Spectrum of **Compound 5a** in $CD_3OD$

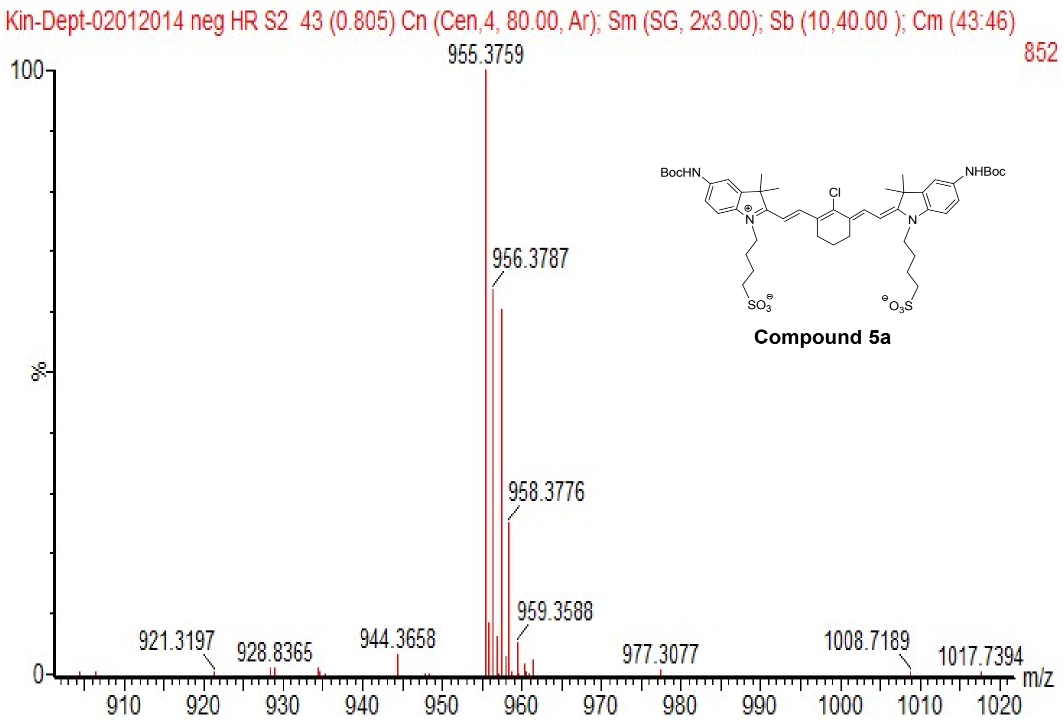


High resolution mass spectrum (ESI-MS) of **Compound 5a**

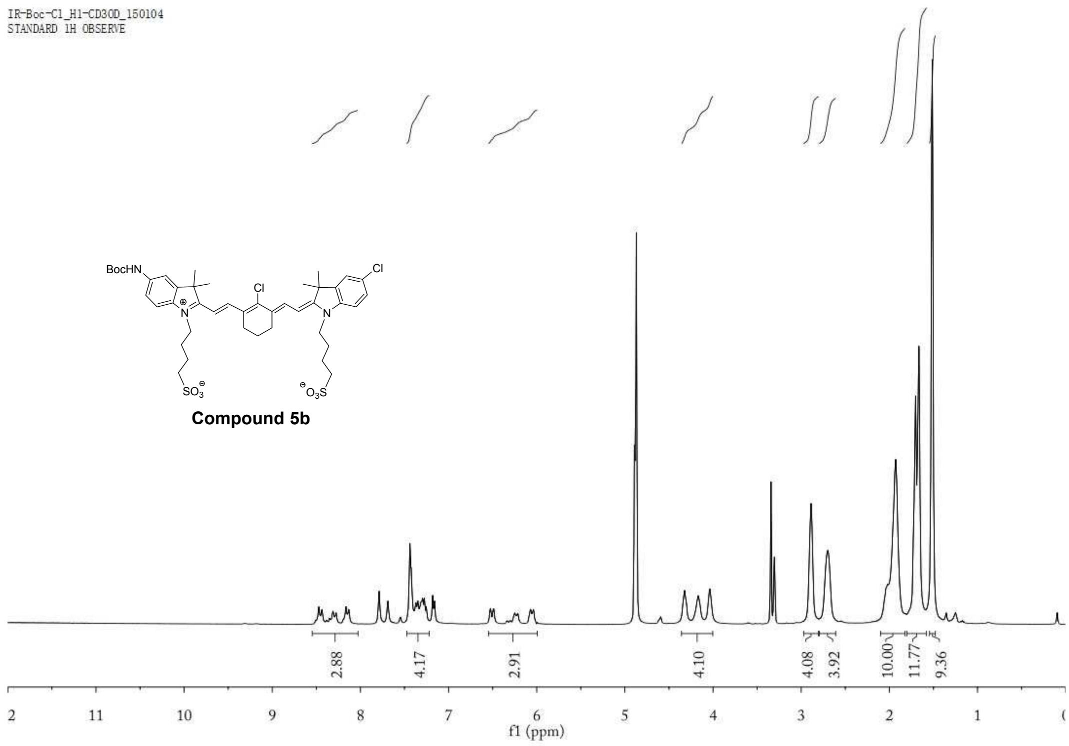


$^{1}H$ NMR Spectrum of **Compound 5b** in $CD_3OD$

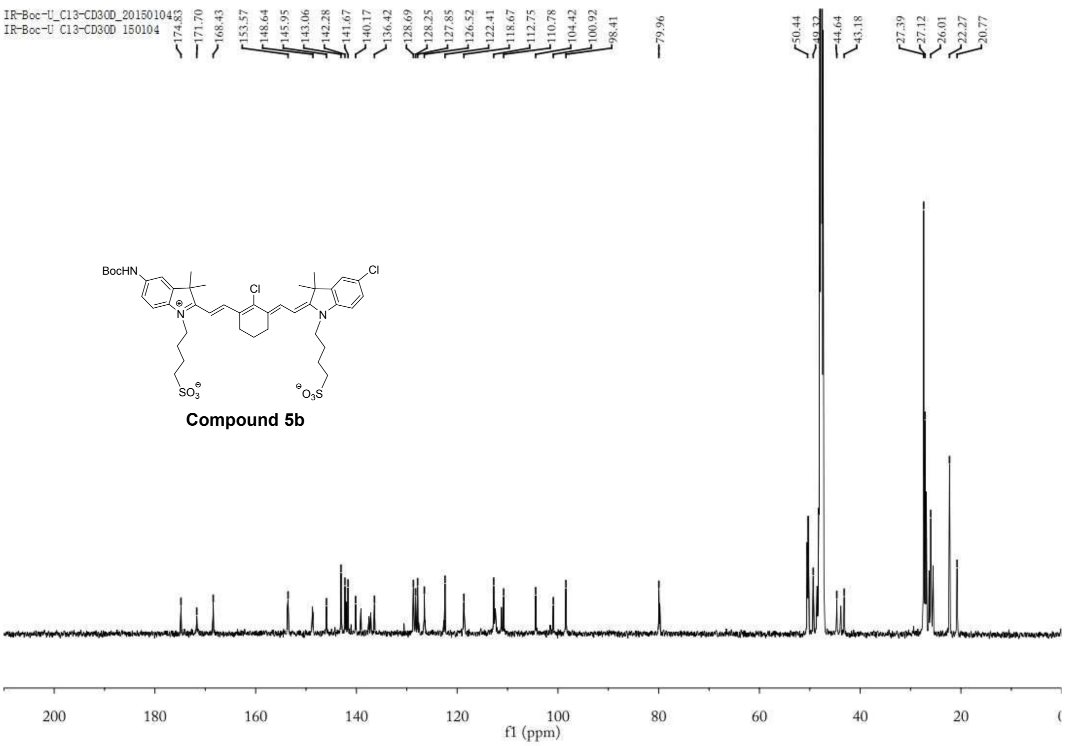


$^{13}$C NMR Spectrum of **Compound 5b** in $CD_3OD$

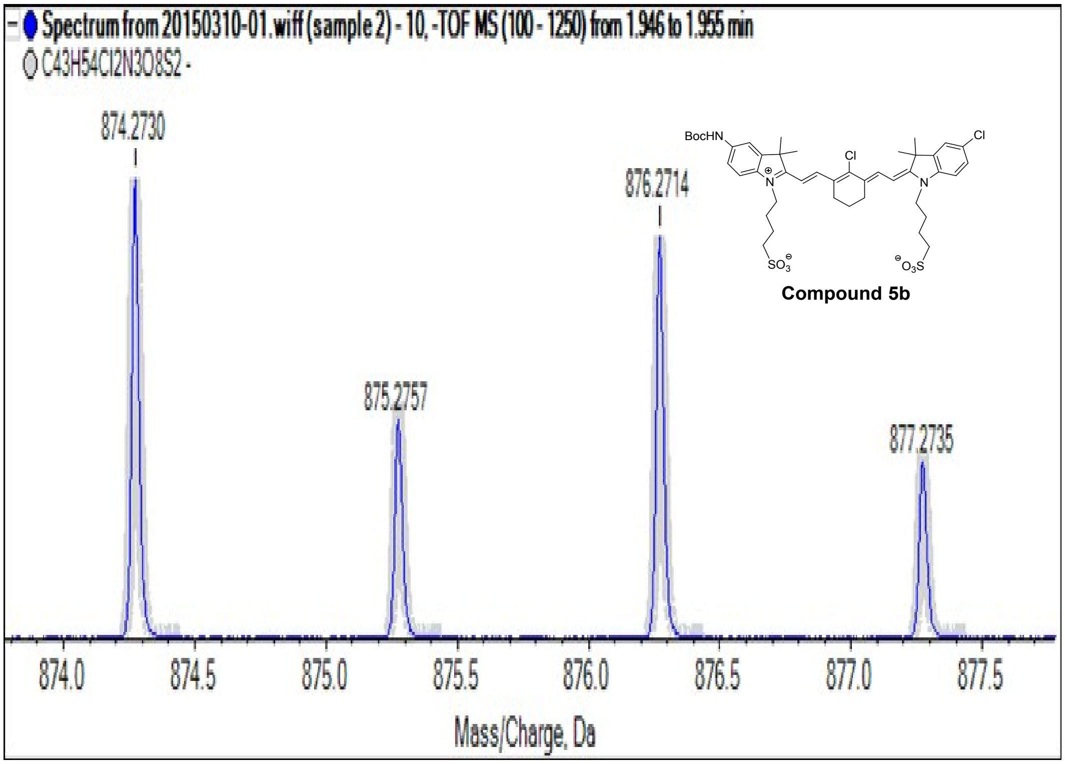


High resolution mass spectrum (ESI-MS) of **Compound 5b**

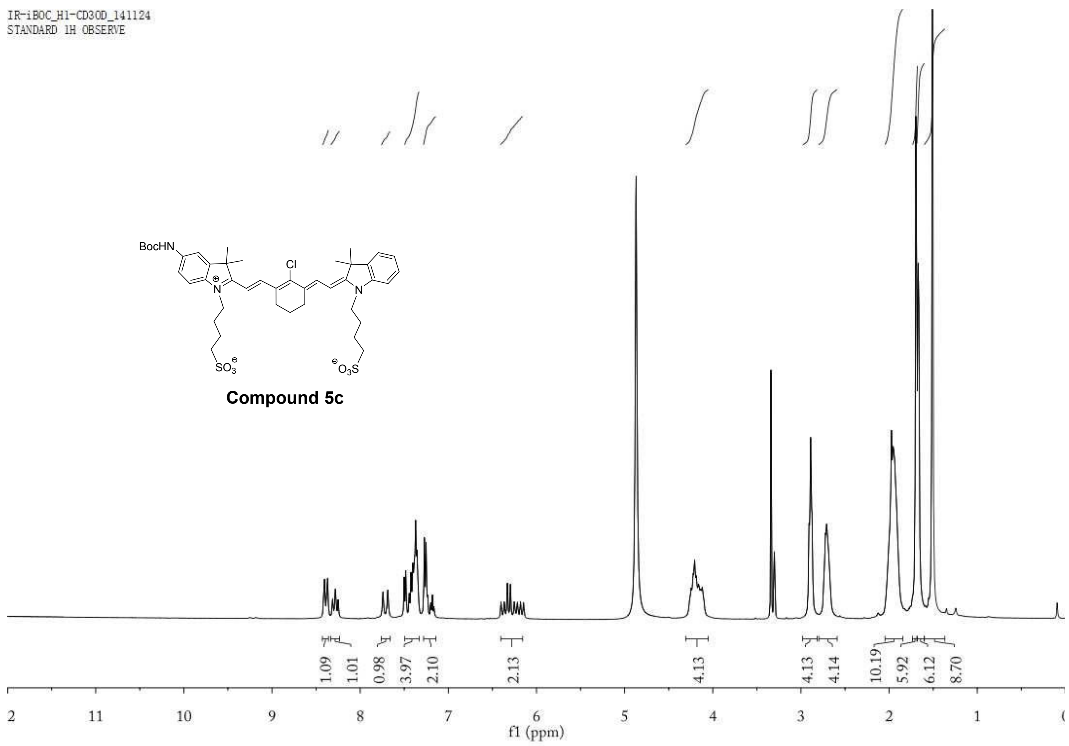

$^{1}$H NMR Spectrum of **Compound 5c** in $CD_3OD$

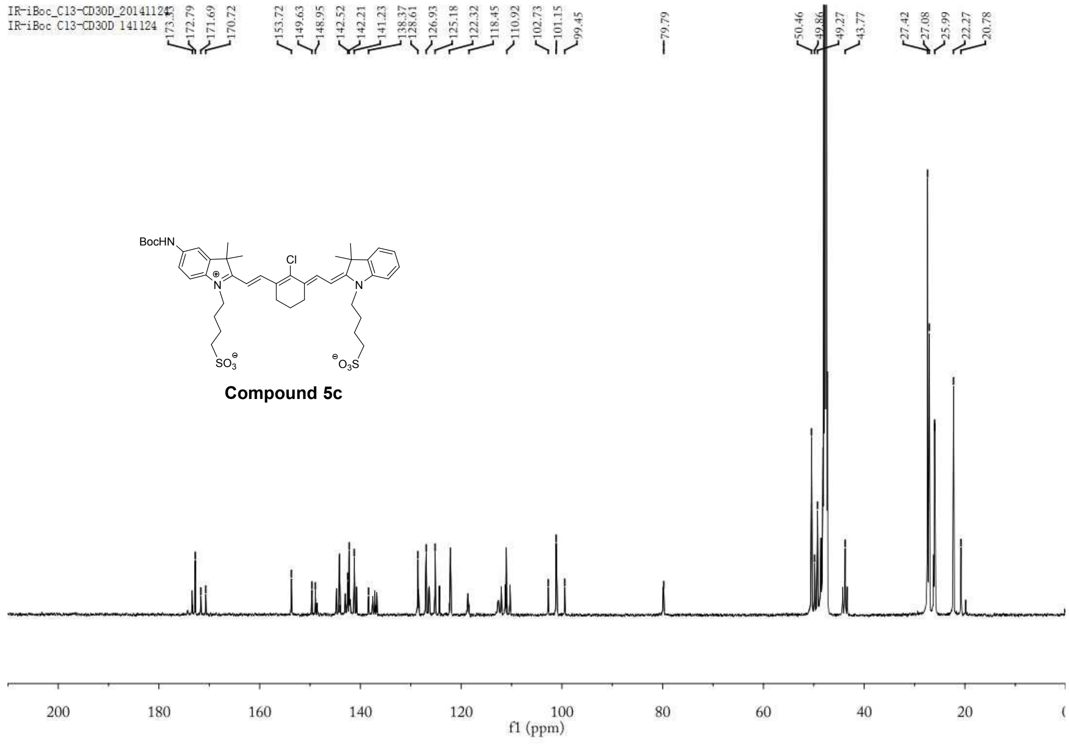

$^{13}$C NMR Spectrum of **Compound 5c** in $CD_3OD$

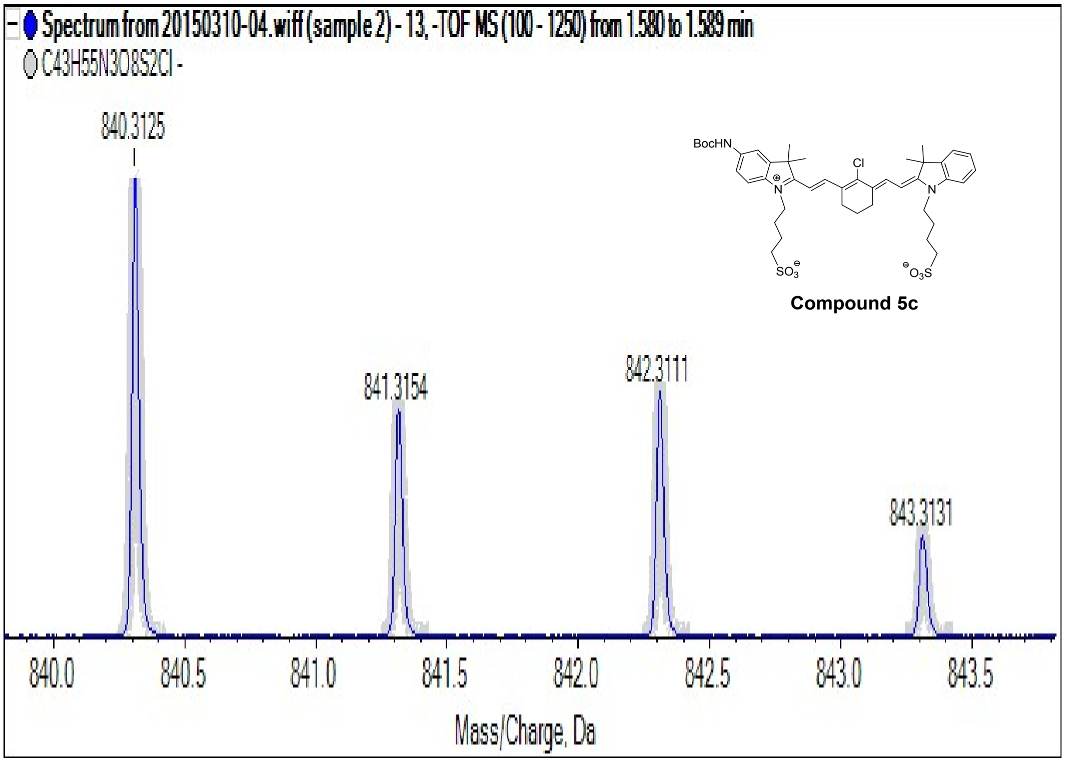


High resolution mass spectrum (ESI-MS) of **Compound 5c**

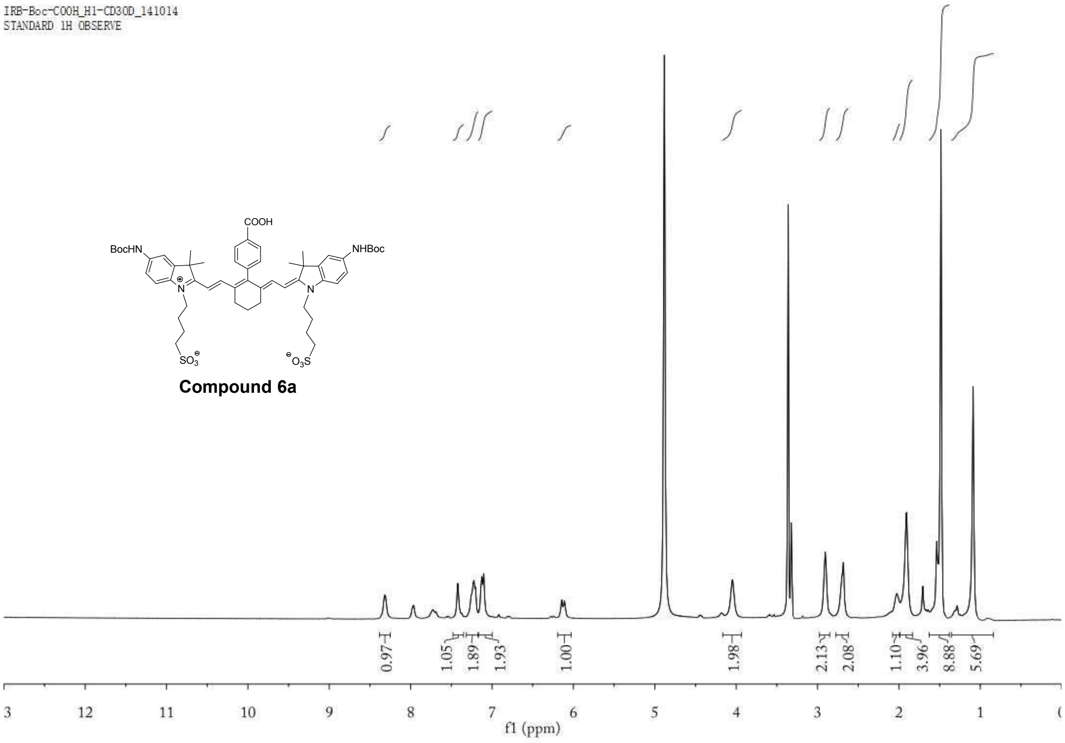


$^{1}$H NMR Spectrum of **Compound 6a** in $CD_3OD$

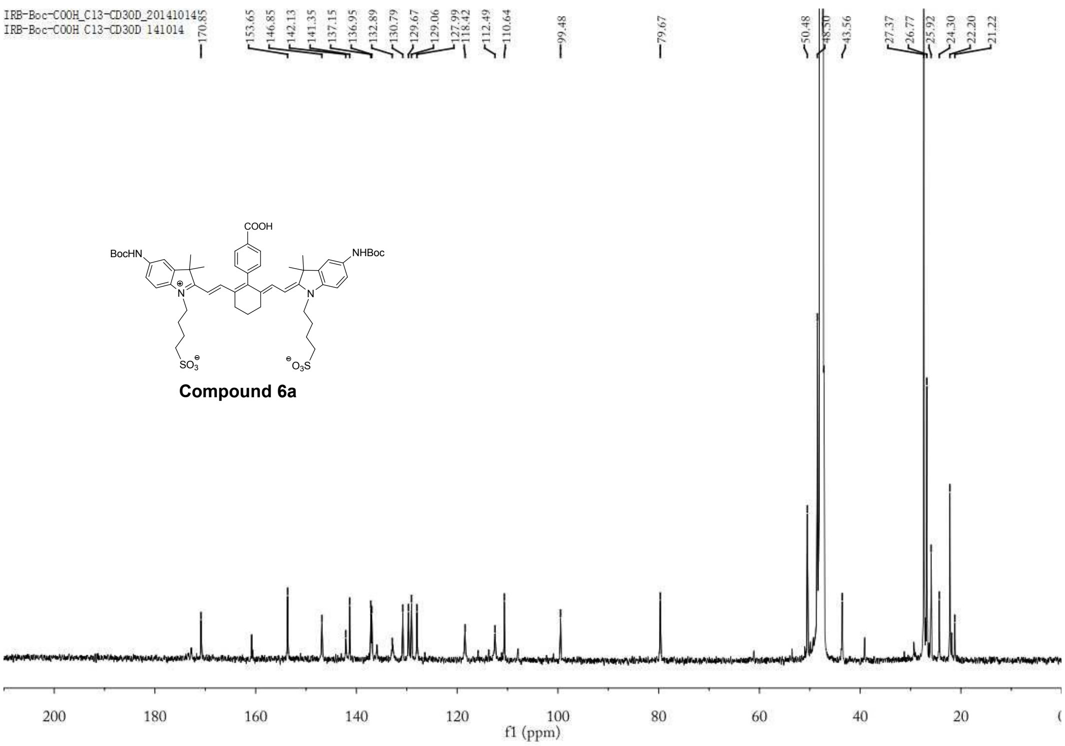


$^{13}$C NMR Spectrum of **Compound 6a** in $CD_3OD$

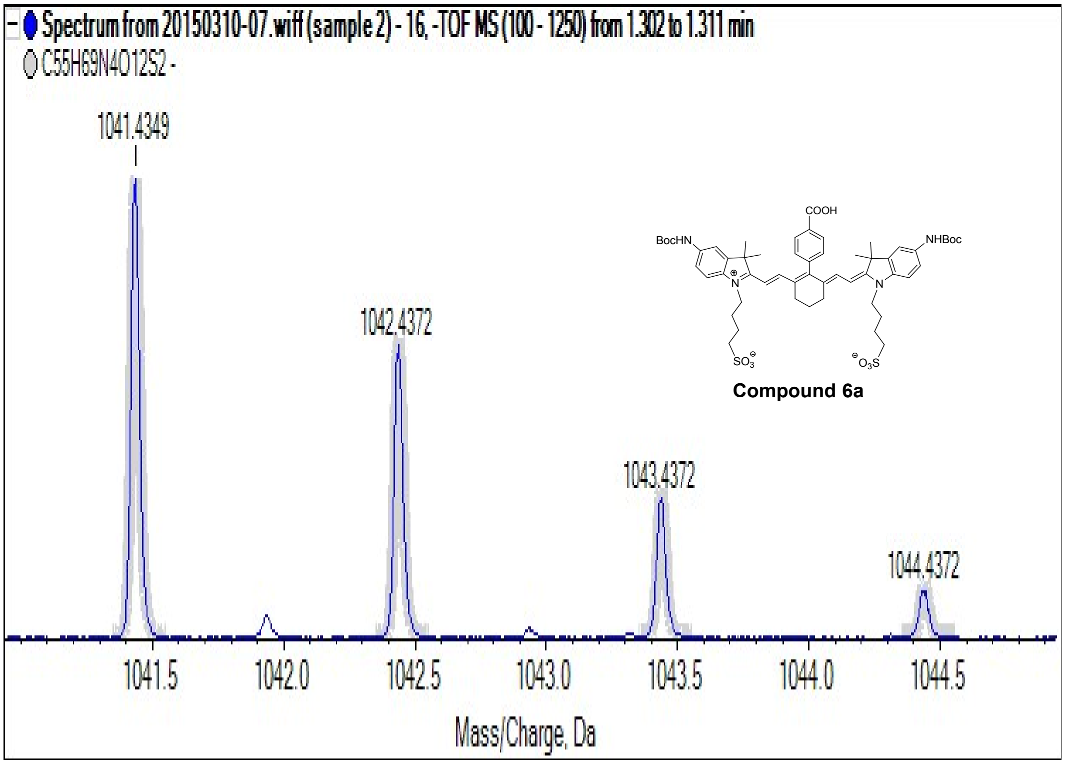


High resolution mass spectrum (ESI-MS) of **Compound 6a**

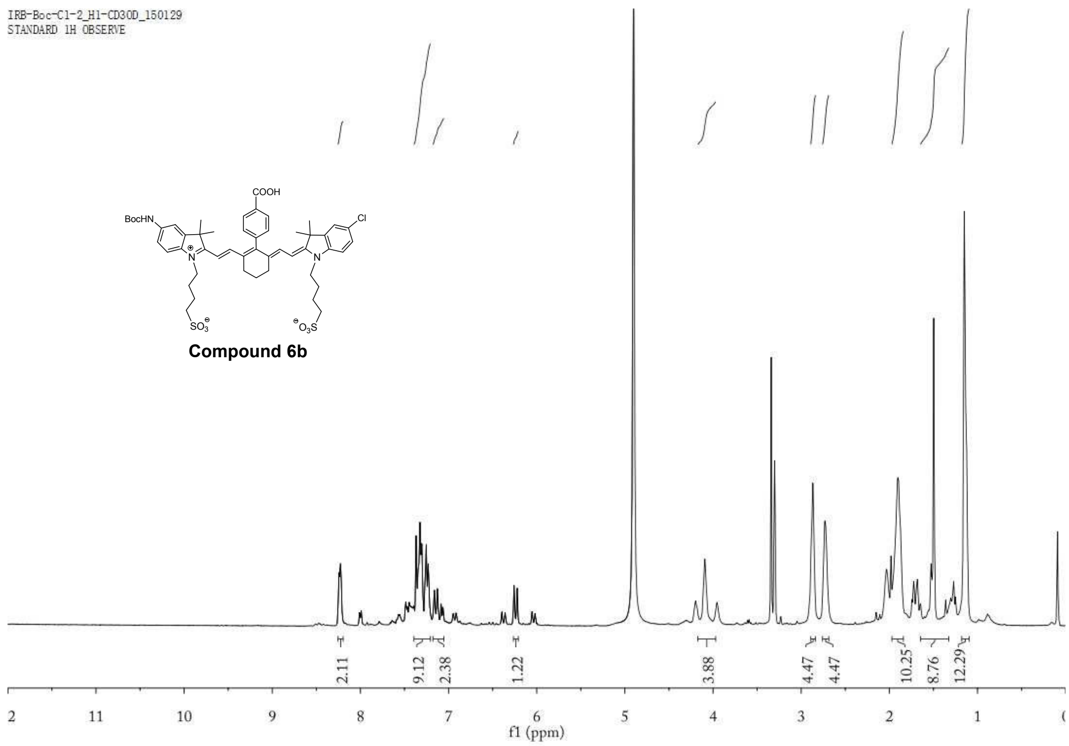


$^{1}H$ NMR Spectrum of **Compound 6b** in $CD_3OD$

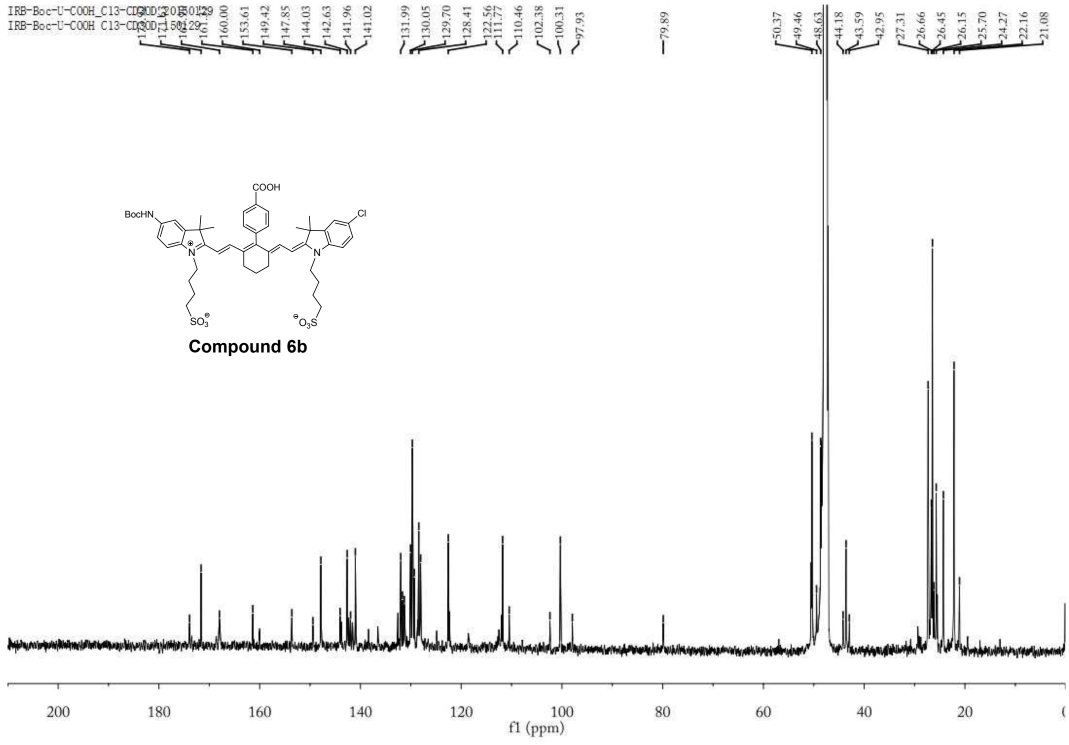


$^{13}C$ NMR Spectrum of **Compound 6b** in $CD_3OD$

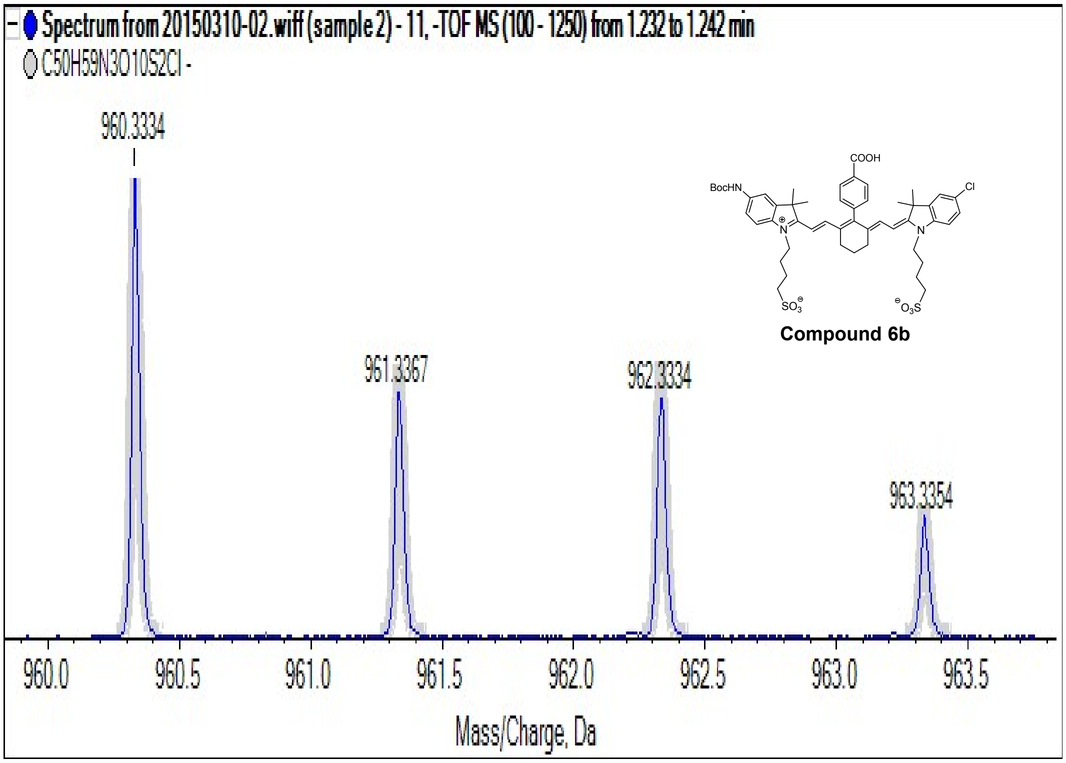


High resolution mass spectrum (ESI-MS) of **Compound 6b**

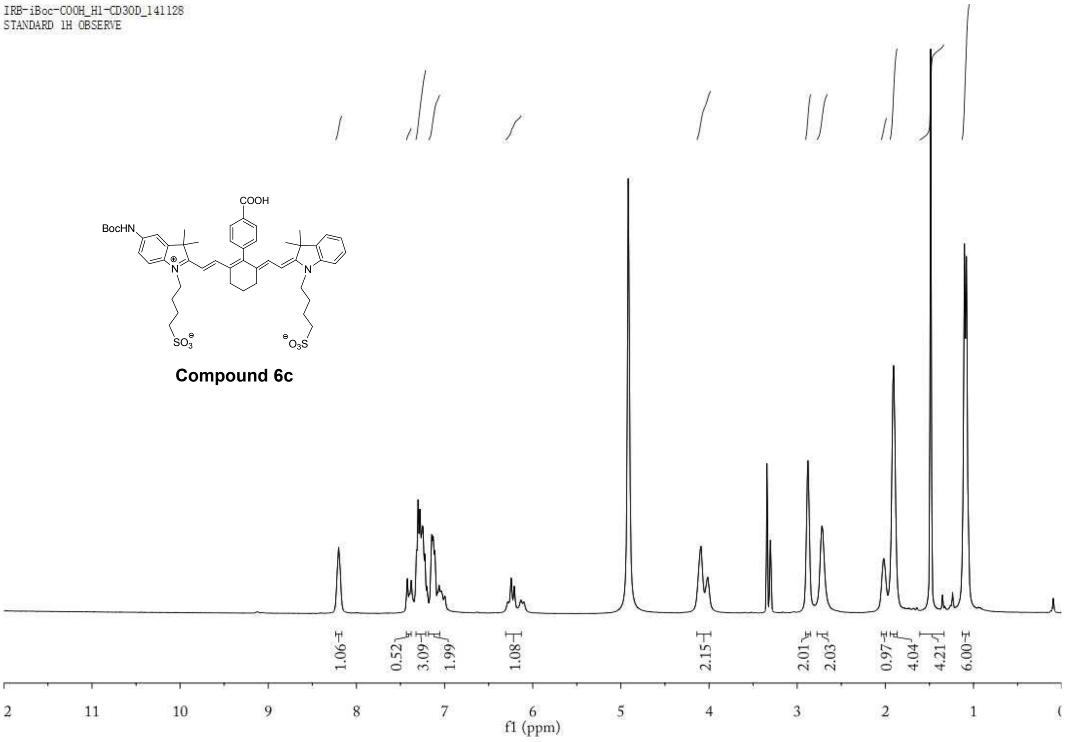


$^1$H NMR Spectrum of **Compound 6c** in $CD_3OD$

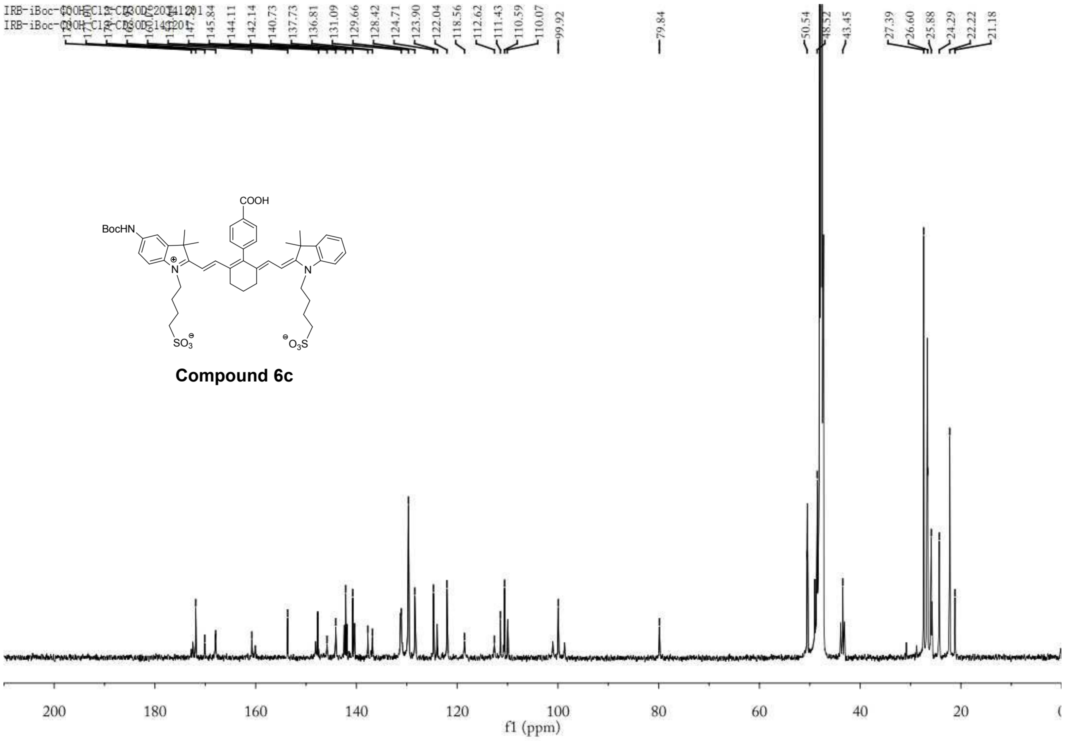


$^{13}$C NMR Spectrum of **Compound 6c** in $CD_3OD$

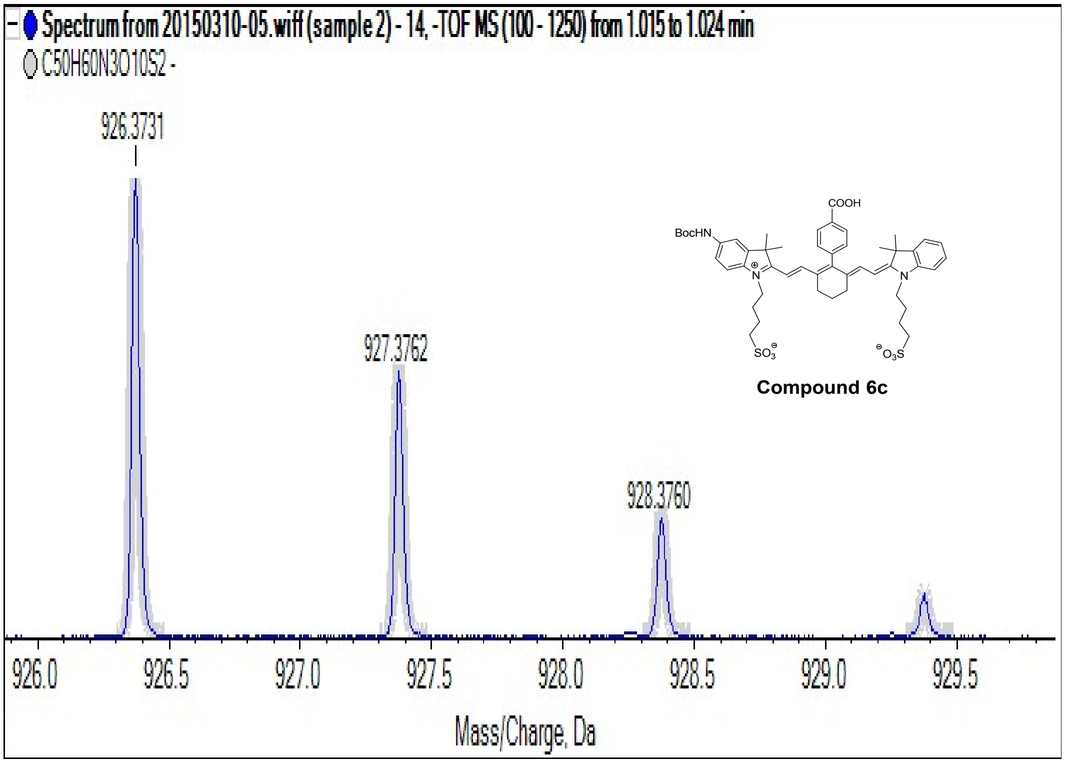


High resolution mass spectrum (ESI-MS) of **Compound 6c**

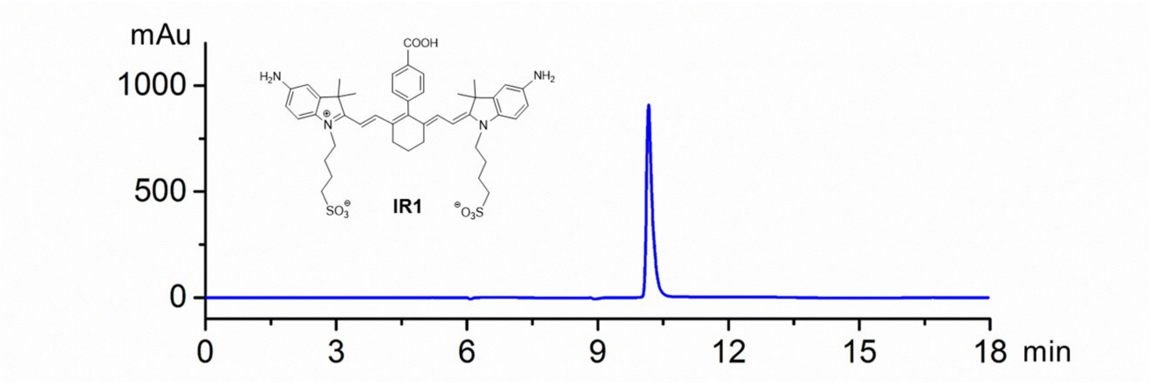


HPLC spectrum of **IR1**

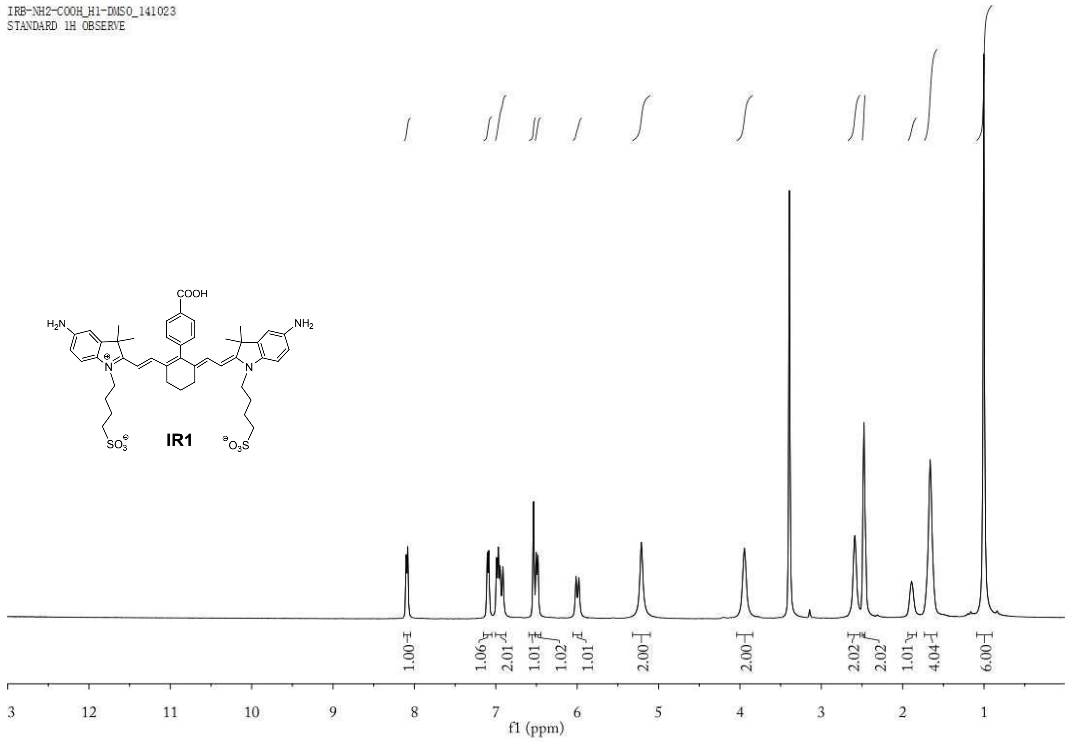


$^1$H NMR Spectrum of **IR1** in DMSO

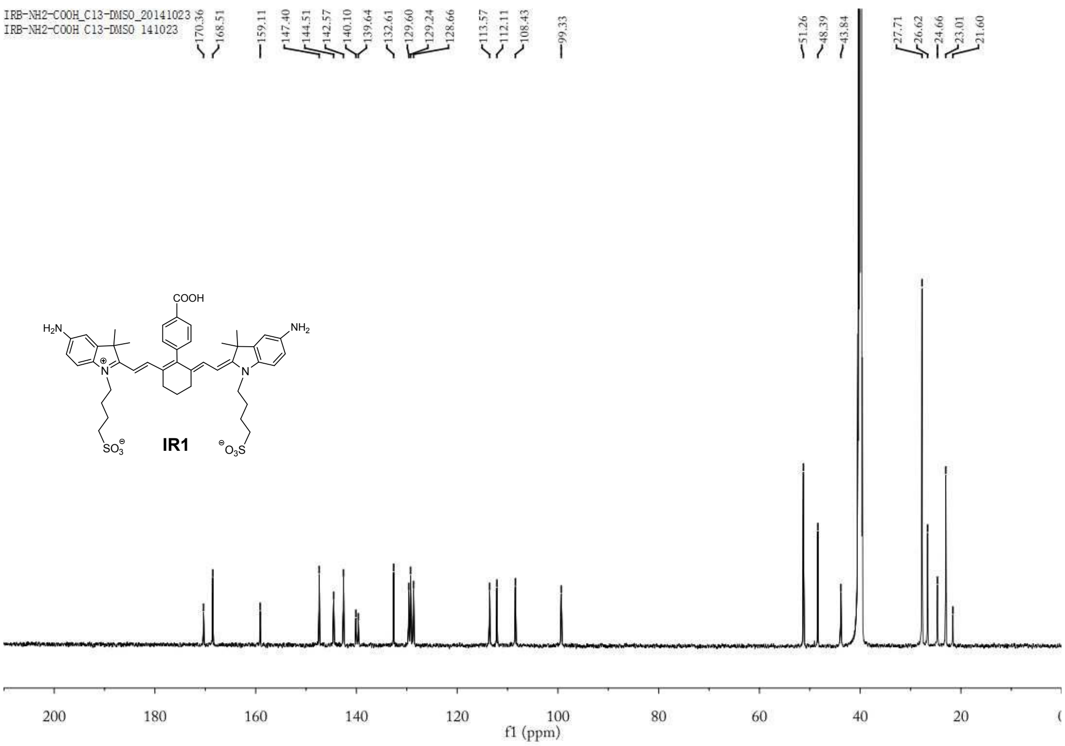


$^{13}$C NMR Spectrum of **IR1** in DMSO

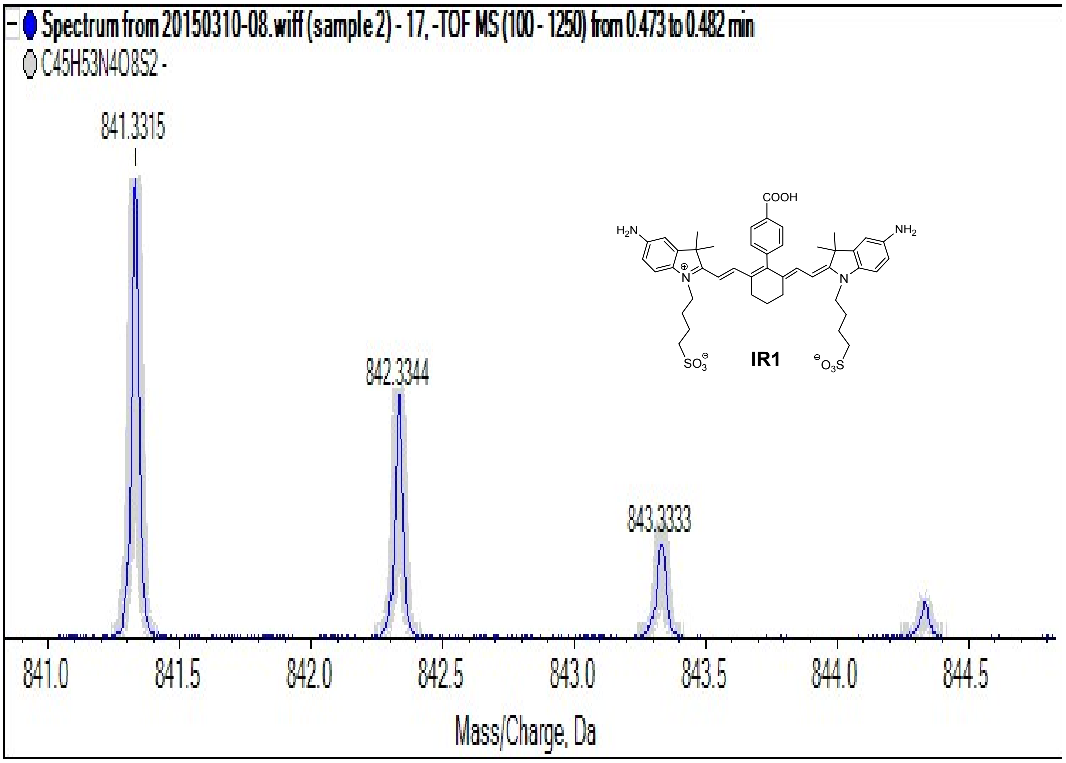


High resolution mass spectrum (ESI-MS) of **IR1**

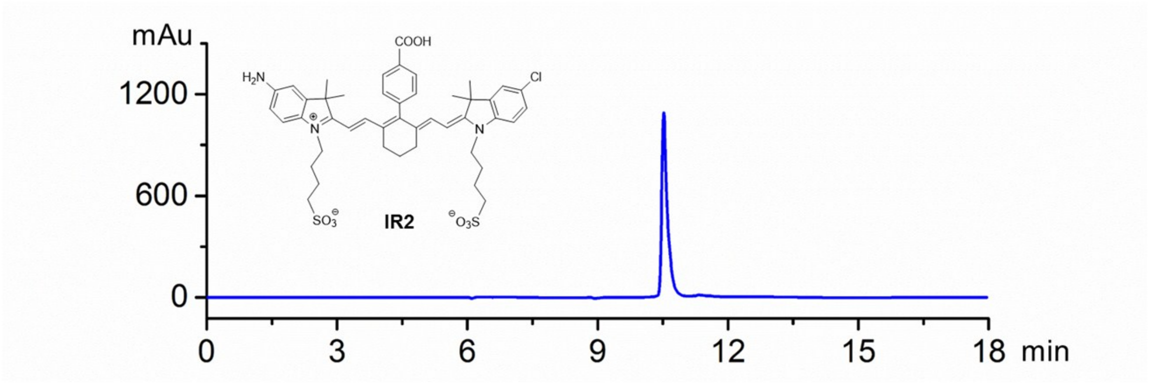


HPLC spectrum of **IR2**

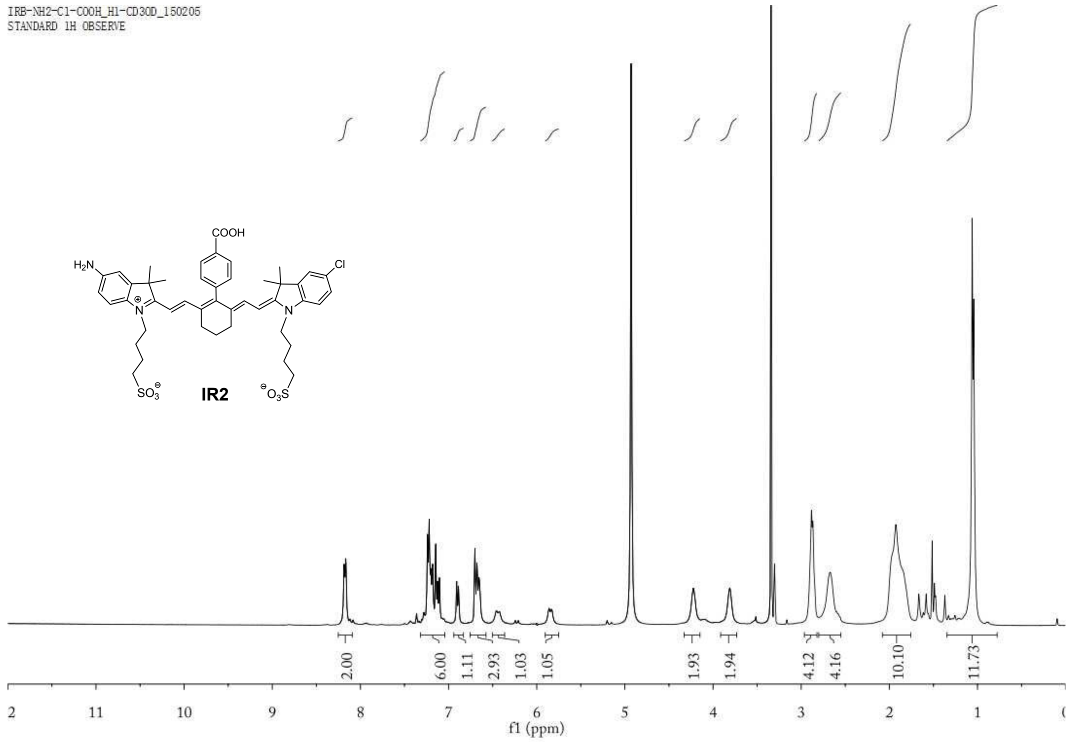


$^{1}$H NMR Spectrum of **IR2** in $CD_3OD$

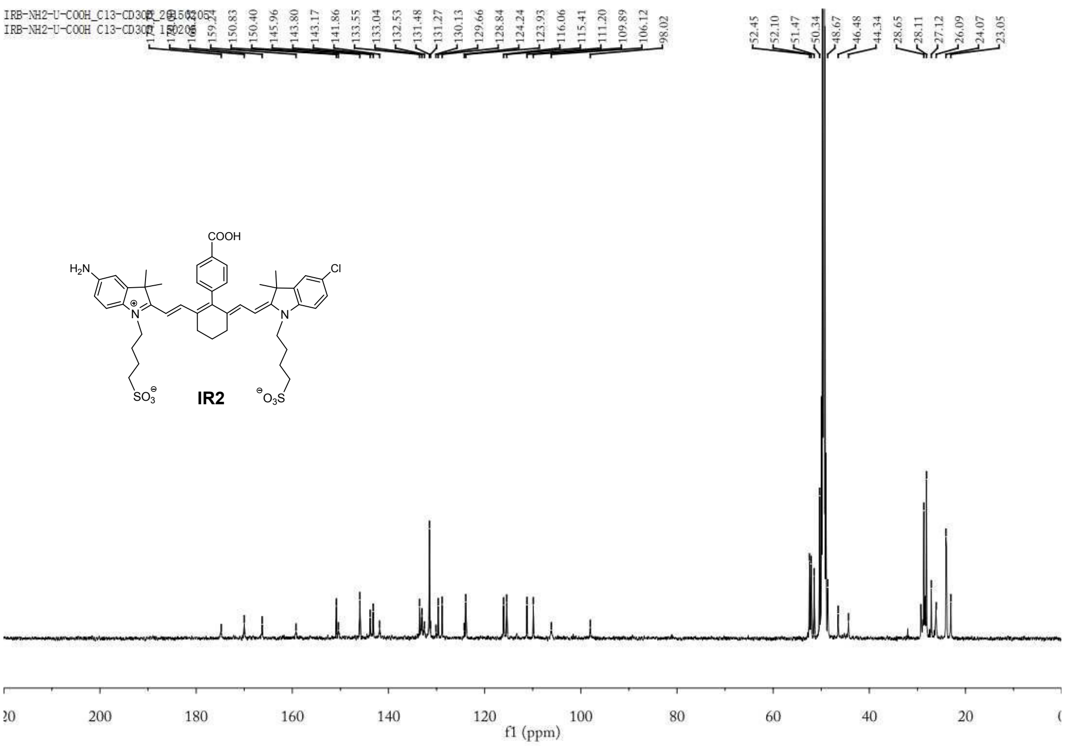


$^{13}C$ NMR Spectrum of **IR2** in $CD_3OD$

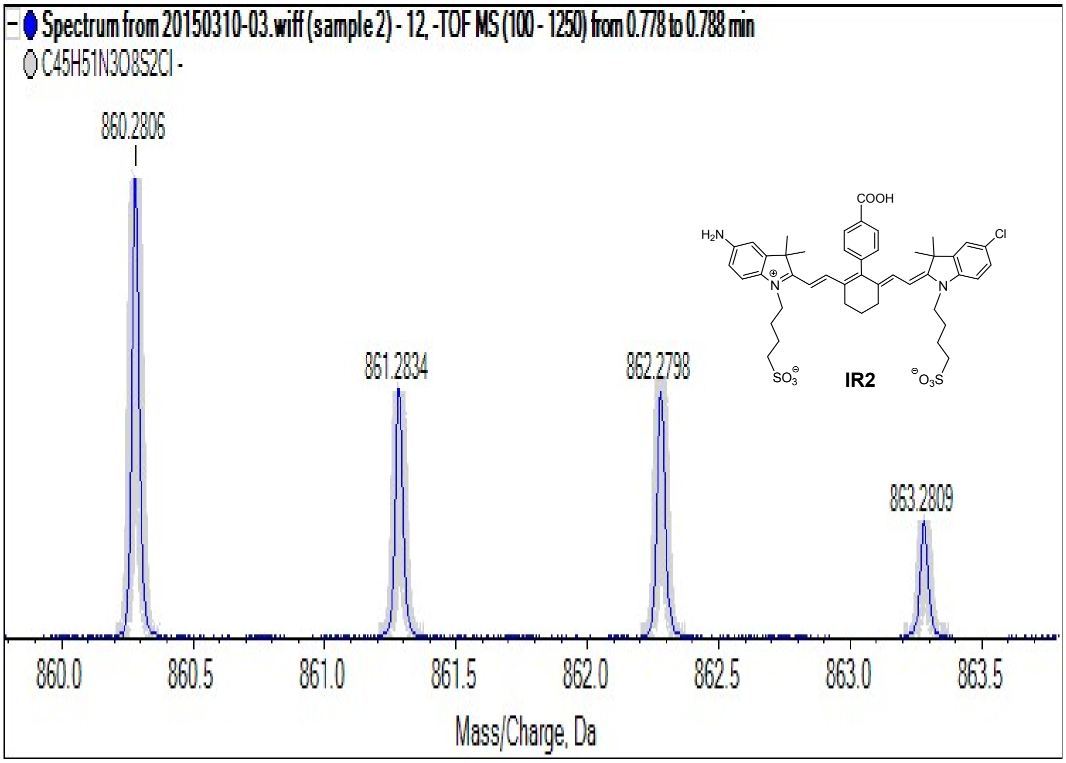


High resolution mass spectrum (ESI-MS) of **IR2**

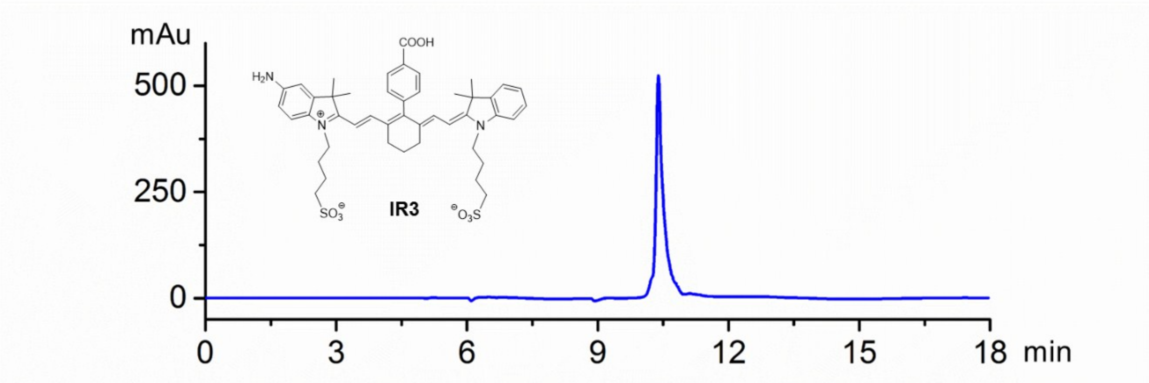


HPLC spectrum of **IR3**

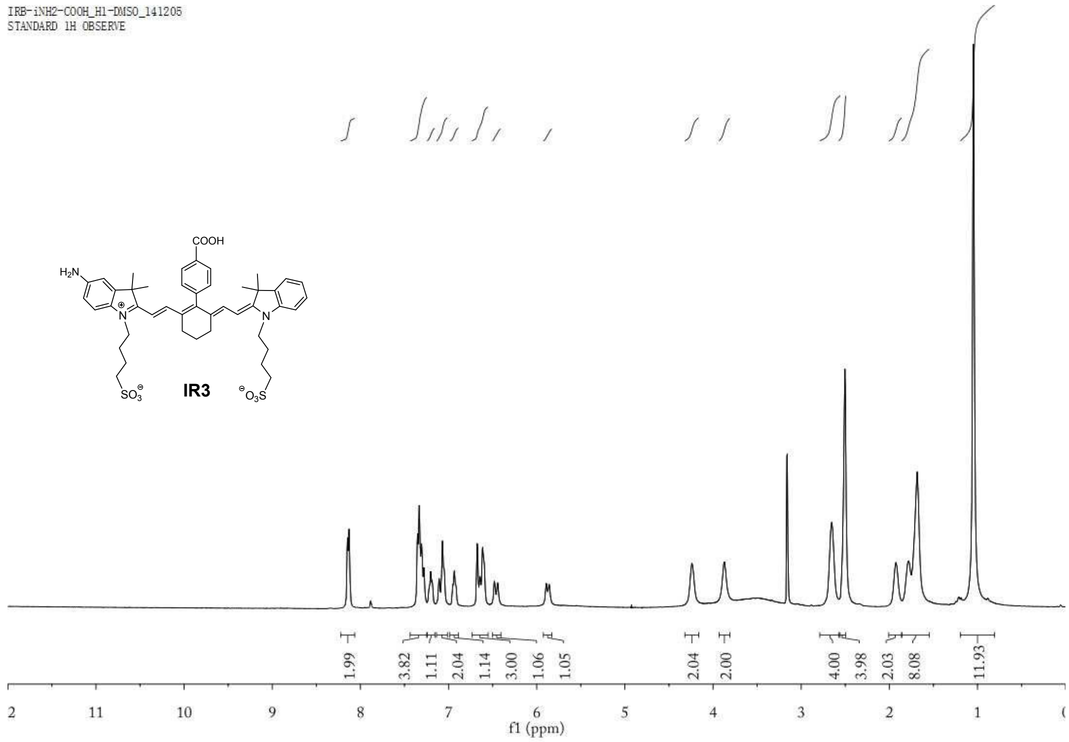


$^1$H NMR Spectrum of **IR3** in DMSO

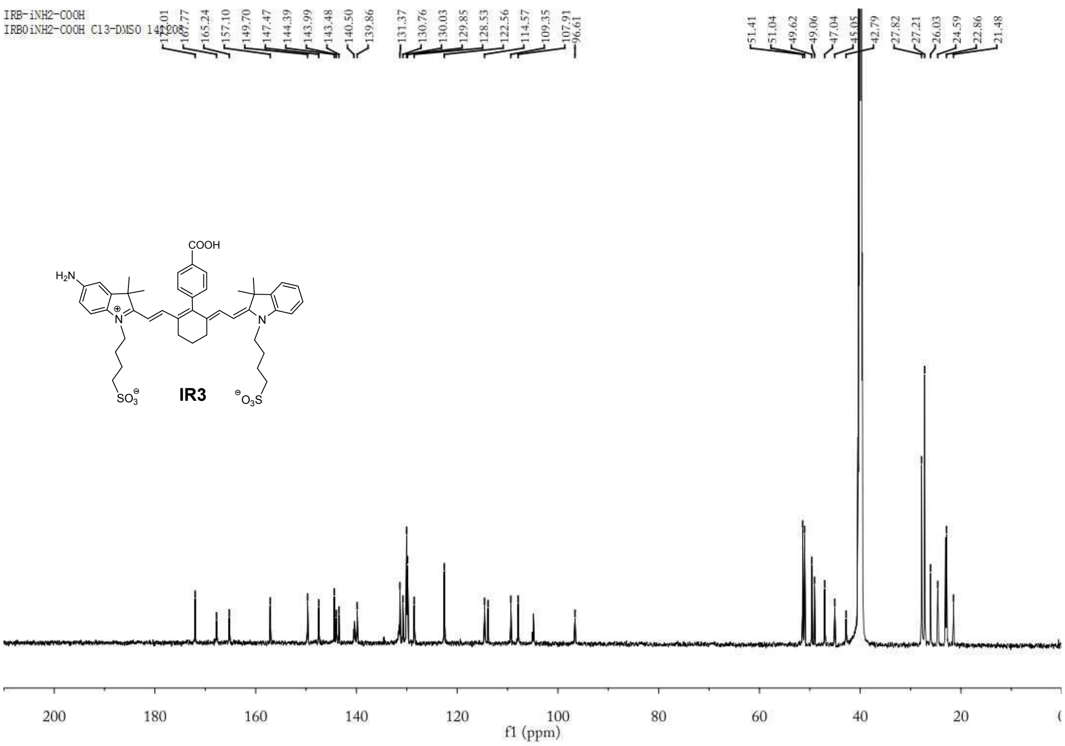


$^{13}C$ NMR Spectrum of **IR3** in DMSO

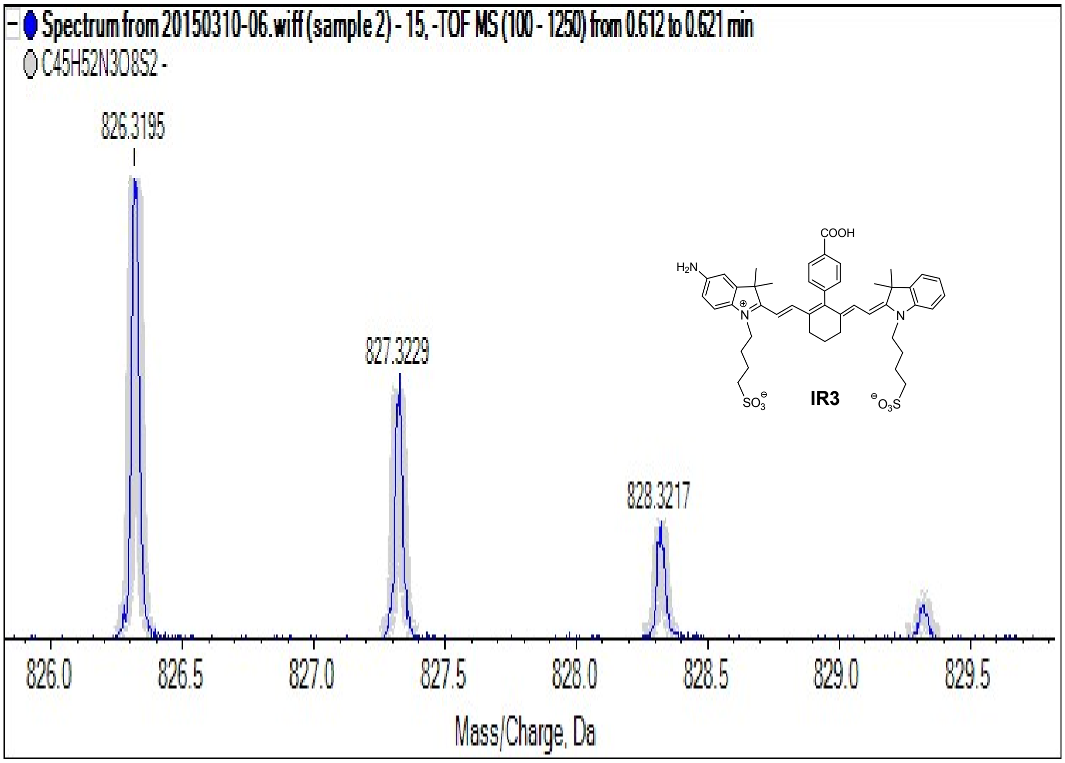


High resolution mass spectrum (ESI-MS) of **IR3**

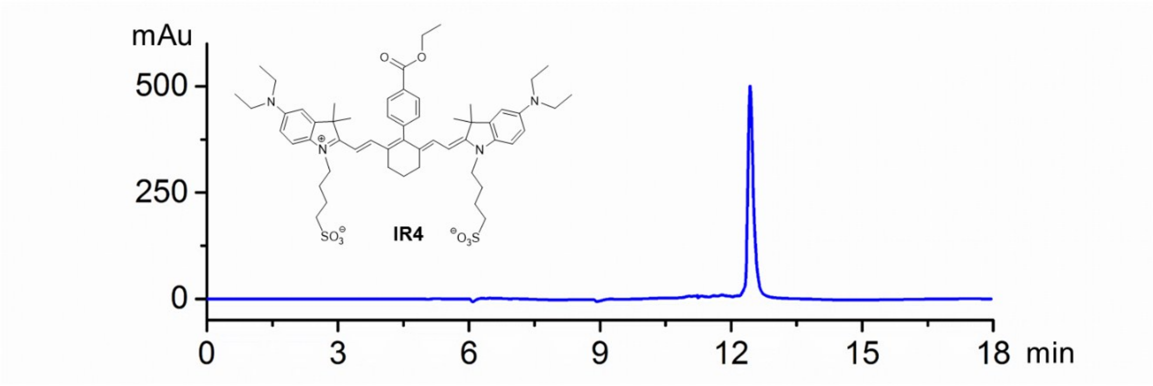


HPLC spectrum of **IR4**

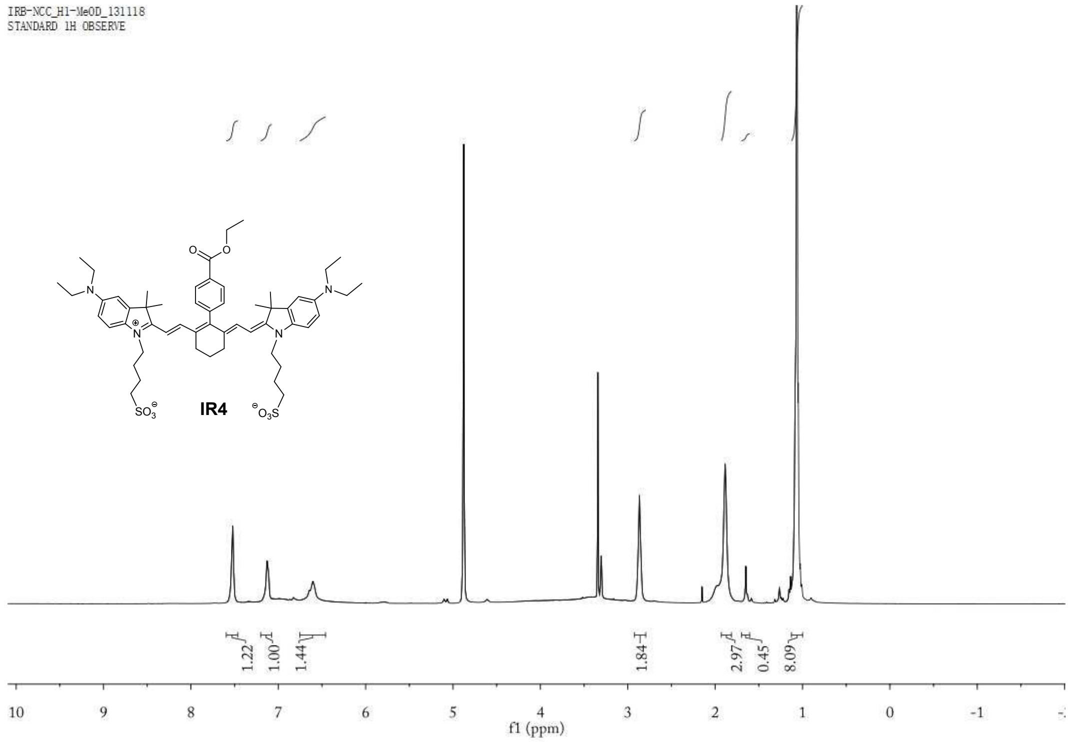


$^1$H NMR Spectrum of **IR3** in $CD_3OD$

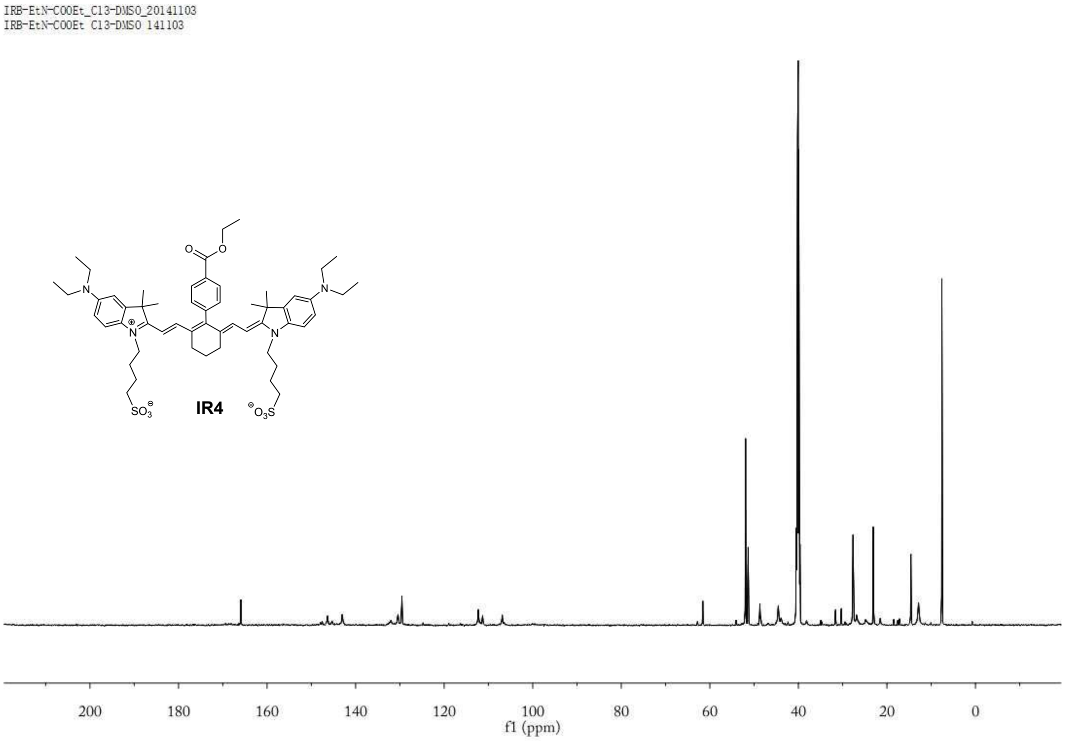


$^{13}$C NMR Spectrum of **IR3** in $CD_3OD$

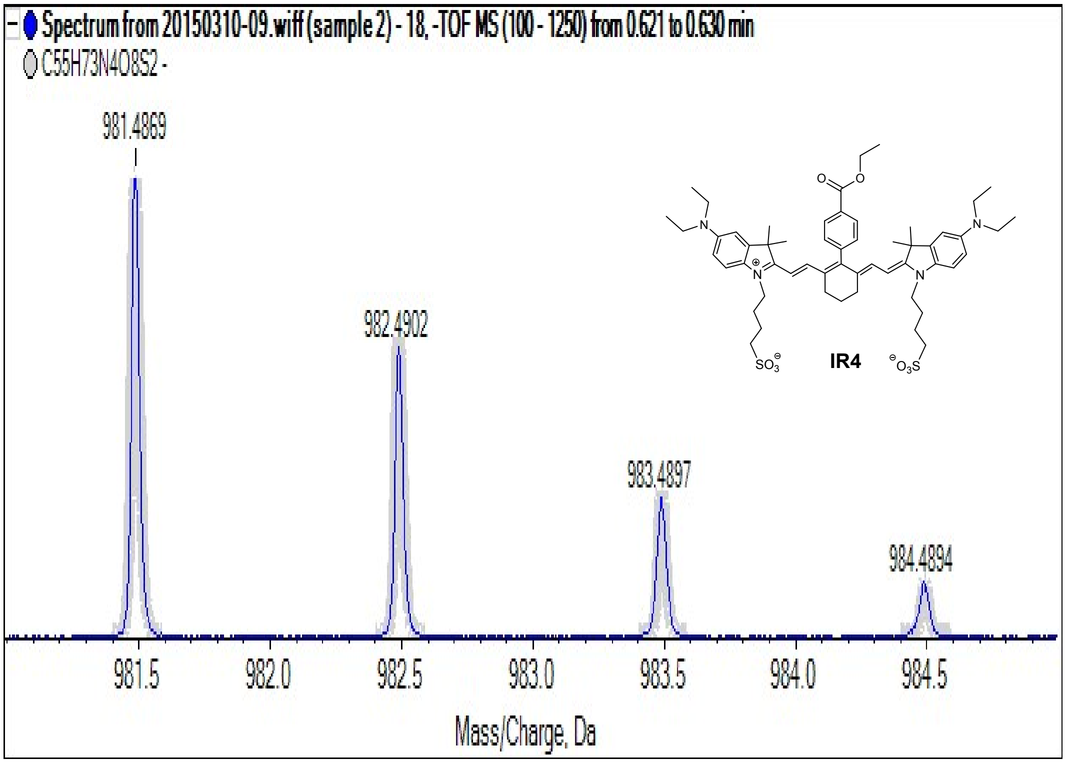


High resolution mass spectrum (ESI-MS) of **IR4**

## 7. Reference

1. Maiti, N. C.; Mazumdar, S.; Periasamy, N., J- and H-aggregates of porphyrins with surfactants: Fluorescence, stopped flow and electron microscopy studies. *J Porphyr Phthalocya* **1998,** *2* (4-5), 369-376.
